# Discovery of autism/intellectual disability somatic mutations in Alzheimer's brains: mutated ADNP cytoskeletal impairments and repair as a case study

**DOI:** 10.1038/s41380-019-0563-5

**Published:** 2019-10-30

**Authors:** Yanina Ivashko-Pachima, Adva Hadar, Iris Grigg, Vlasta Korenková, Oxana Kapitansky, Gidon Karmon, Michael Gershovits, C. Laura Sayas, R. Frank Kooy, Johannes Attems, David Gurwitz, Illana Gozes

**Affiliations:** 1 The First Lily and Avraham Gildor Chair for the Investigation of Growth Factors; The Elton Laboratory for Neuroendocrinology; Department of Human Molecular Genetics and Biochemistry, Sackler Faculty of Medicine, Sagol School of Neuroscience and Adams Super Center for Brain Studies, Tel Aviv University, Tel Aviv, 69978 Israel; 2 BIOCEV, Institute of Biotechnology CAS, Průmyslová 595, 252 50, Vestec, Czech Republic; 3 The Nancy & Stephen Grand Israel National Center for Personalized Medicine, Weizmann Institute of Science, Rehovot, Israel; 4 Instituto de Tecnologías Biomédicas (ITB), Universidad de La Laguna (ULL), Tenerife, Spain; 5 Department of Medical Genetics, University of Antwerp, Antwerp, Belgium; 6 Institute of Neuroscience and Newcastle University Institute of Ageing, Newcastle University, Newcastle upon Tyne, UK

**Keywords:** Neuroscience, Predictive markers

## Abstract

With Alzheimer’s disease (AD) exhibiting reduced ability of neural stem cell renewal, we hypothesized that de novo mutations controlling embryonic development, in the form of brain somatic mutations instigate the disease. A leading gene presenting heterozygous dominant de novo autism-intellectual disabilities (ID) causing mutations is activity-dependent neuroprotective protein (ADNP), with intact ADNP protecting against AD-tauopathy. We discovered a genomic autism ADNP mutation (c.2188C>T) in postmortem AD olfactory bulbs and hippocampi. RNA-Seq of olfactory bulbs also identified a novel ADNP hotspot mutation, c.2187_2188insA. Altogether, 665 mutations in 596 genes with 441 mutations in AD patients (389 genes, 38% AD—exclusive mutations) and 104 genes presenting disease-causing mutations (OMIM) were discovered. OMIM AD mutated genes converged on cytoskeletal mechanisms, autism and ID causing mutations (about 40% each). The number and average frequencies of AD-related mutations per subject were higher in AD subjects compared to controls. RNA-seq datamining (hippocampus, dorsolateral prefrontal cortex, fusiform gyrus and superior frontal gyrus—583 subjects) yielded similar results. Overlapping all tested brain areas identified unique and shared mutations, with ADNP singled out as a gene associated with autism/ID/AD and presenting several unique aging/AD mutations. The large fusiform gyrus library (117 subjects) with high sequencing coverage correlated the c.2187_2188insA ADNP mutation frequency to Braak stage (tauopathy) and showed more ADNP mutations in AD specimens. In cell cultures, the ADNP-derived snippet NAP inhibited mutated-ADNP-microtubule (MT) toxicity and enhanced Tau–MT association. We propose a paradigm-shifting concept in the perception of AD whereby accumulating mosaic somatic mutations promote brain pathology.

## Introduction

Adult neurogenesis in humans takes place exclusively in the dentate gyrus of the hippocampus and in the subventricular zone of the olfactory bulb, while newborn neurons migrate to other brain regions. Impaired adult neurogenesis, implicated in Alzheimer’s disease (AD), is underlined by reduced ability for neural stem cell renewal in these brain tissues [1, 2]. Chromosomal changes usually associated with aberrant developmental processes are also found in the aging brain [3] and we now asked whether rare developmental de novo mutations might also be found in the form of somatic mutations in the aging brain.

In ~0.17% of the autism-intellectual disabilities (ID) cases, activity-dependent neuroprotective protein (ADNP) [4, 5] is mutated de novo [6, 7], representing one of the most prevalent mutated heterozygous dominant genes leading to autism/ID [8–10]. ADNP [4] is critical for neurogenesis [11] essential for brain formation [12] and function [13], controlling hundreds of key regulatory genes in vivo [13, 14]. As such, *ADNP* mRNA content dysregulation has been implicated not only in autism [15–17] but also in schizophrenia [18, 19], Parkinson’s disease [20] and AD [13, 20, 21]. Thus, complete proteomics revealed ADNP as a unique protein decreasing in AD patient serum samples [22], and a positive correlation was found between ADNP serum content and elderly subject performance in IQ tests [20]. Furthermore, ADNP directly regulates the expression of apolipoprotein E (*APOE*) [13], in a sex-dependent manner [21], with *APOE4* being the major risk gene for sporadic AD.

The most devastating outcome of AD is dramatically decreased cognition. The progression of the disease is measured as decreases in cognitive abilities, which correspond to increases amyloid plaque burden, Tau pathology and brain degeneration, with AD being the most prevalent tauopathy [15]. ADNP deficiency has been associated with aging-related increase in tauopathy with *Adnp*^*+/-*^ mice exhibiting age-driven tauopathy, neurodegeneration, and cognitive deficits [13]. An eight-amino-acid peptide, NAP (NAPV**SIP**Q), identified as the smallest active snippet of ADNP, protects against ADNP deficiencies [13], including tauopathy [23] and cognitive deficits [24] and displays potent neuroprotection against multiple toxic insults [4]. NAP (also known as davunetide, or CP201) was identified as a neurotrophic factor, stimulating synapse formation through microtubule (MT) interaction [23, 25].

MTs are the major component of the neuronal cytoskeleton. MT dynamics plays a key regulatory role during axon outgrowth and regeneration. MTs are decorated by MT-associated proteins (MAPs) and MT plus-end tracking proteins (+TIPs), which concentrate and act at growing MT ends. Numerous+TIPs localize to MT plus-ends in MT-end-binding-proteins (EBs)-dependent manner [26]. Together, EBs and the related+TIPs regulate MT dynamics and MT connection to cellular structures, and thus determine the fate of MT growing ends [27].

We discovered direct interactions of ADNP with the EB family of proteins and showed that EB1 and EB3 are binding targets for NAP through the **SIP** motif on ADNP/NAP (NAPV**SIP**Q). In this respect, NAP enhances ADNP–EB3 interaction [25], and EB3 silencing inhibits NAP-driven dendritic spine formation [25]. Furthermore, NAP/ADNP by binding to EB3, which in turn binds Tau, enhance Tau association with the MT shaft, protecting the MTs [23, 28], MT-regulated axonal transport [14] and inhibiting tauopathy [13, 29].

Here, we concentrated on three previous findings. (1) The ADNP syndrome, like AD, is characterized by ID. (2) Neurogenesis/synaptic plasticity is impaired in autism and AD and ADNP is critical for both. (3) Intact ADNP is required for MT dynamics and stability through Tau interactions, while AD is characterized by shorter MTs [30] and tauopathy [15]. We therefore hypothesized de novo somatic rare mutations of *ADNP* as not only occurring during early development but also arising in a mosaic form in the aging brain and affecting AD precipitation and progression. Furthermore, given the protection of NAP against *Adnp* haploinsuffiency [13], we conjectured NAP protection against mutated*-ADNP* associated MT deficiencies.

Using postmortem AD and control brains (olfactory bulbs and hippocampi), we revealed a hotspot for somatic AD *ADNP* mutations including the novel frameshift, c.2187_2188insA, p.Arg730Thrfs*4 with mutation frequency correlated to Braak stage (tauopathy) and aging. Combined with public datamining of RNA-seq results from hippocampi, dorsolateral prefrontal cortex (DLPFC), fusiform gyri, and superior frontal gyrus, we identified pathogenic mutations in >9000 genes, with a third representing brain region unique AD mutations including autism/ID and cytoskeletal functions. These findings present a novel molecular understanding of AD.

## Methods

### RNA and DNA extraction

Postmortem tissue samples from elderly donors (20 controls, 20 AD patients), all exhibiting tauopathy (Braak stage I–IV controls and IV–VI AD, Table S1) [31] were obtained from the Newcastle Brain Tissue Resource (NBTR). All was approved by the joint Ethics Committee of Newcastle/North Tyneside Health Authority (following NBTR brain banking procedures) and Tel Aviv University Ethics Committee. Full written informed consent was obtained from tissue donors or their relatives where appropriate. All AD cases fulfilled the criteria for high AD neuropathological changes according to the National Institute on Aging-Alzheimer's Association (NIA-AA) guidelines. Tissues were homogenized using a Bullet Blender (Next Advance, Inc., NY). RNA and DNA were extracted from the same tissue with a ZR-Duet DNA-RNA MiniPrep Plus kit (Zymo Research, Irvine, CA).

### ADNP ddPCR

20 μl ddPCR reactions were prepared (Bio-Rad Laboratories Ltd. Rishon Le Zion, Israel & Prague Czech Republic): including 10 μl of ddPCR™ Supermix for Probes (No dUTP), 1 μl of PrimePCR™ ddPCR™ Assay, 120 ng DNA, and nuclease-free water. Two targets were detected simultaneously with different fluorescence probes: the FAM probe detected the mutated sequence NM_001282531.2(ADNP):c.2188C>T (p.Arg730*), (unique assay Id: dHsaMDS971559989), or in separate reactions, the NM_015339.3(ADNP):c.2157C>G (p.Tyr719*) (unique assay Id: dHsaMDS423261257, supplemental methods). The HEX probe was used to detect the control sequence (Gene Id: 23394). The annealing temperature for various sets of primers and probes was established by previous gradient runs. After droplet generation, PCR was performed in a C1000 as follows, 95 °C 10 min, 40 cycles of 94 °C 30 s, 54 °C 1 min, 98 °C 10 min. The droplets were analyzed with a QX200 instrument, and the data were processed by the Quantasoft software, with the rare event detection (RED) mode according to the digital MIQE guidelines [32].

### cDNA library preparation

A cDNA library was prepared from 1000 ng RNA samples with a SMARTer® Stranded Total RNA Sample Prep Kit—HI Mammalian (Clontech Laboratories, CA). The SMARTer® kit is suitable for total RNA with a RIN between 3 and 10. The cDNA library was validated using the 4200 Tape Station System (Agilent Technologies, Santa Clara, CA) with High Sensitivity DNA Kit. The cDNA library was quantified using Qubit™ fluorometric (Thermo Fisher Scientific, Waltham, MA). Four pools were prepared from the RNA libraries (10 nM) and were sequenced on four lanes.

### RNA-Seq

cDNA sequencing was performed as a single read 60 on four lanes of an Illumina Hi-Seq. Sequencing depth was ~17 M reads/sample. Raw reads were processed by trimming poly-A/T stretches and Illumina adapters using cutadapt [33] and resulting reads shorter than 30 bp were discarded. The trimmed reads were mapped to the human genome (Ensembl GRCh38) using STAR v2.4.2a [34] with default parameters (supplemental methods).

### Variant calling

Trimmed reads were mapped to the human genome (Ensembl’s GRCh38) using STAR v2.4.2a [34] with default parameters and twopassMode set to basic. Reads were then deduplicated using Picard, except for the olfactory bulb data, where it was not performed due to the short read length (60 bp). Mapped reads were further processed with GATK’s v.3.7 [35]. SplitNCigarReads. Next, variants were called using GATK’s HaplotypeCaller with ploidy set to 10 except for chromosomes 10 and 15 in GSE95587, the fusiform gyrus tissue dataset, where it was set to 8 due to technical limitations that did not allow to use ploidy10. Variants were filtered with the following values for SNPs and Indels respectively: QD < 2.0, FS > 30.0, MQ < 40.0, MQRankSum < −12.5, ReadPosRankSum < −8.0 and QD < 2.0, FS > 30.0 and ReadPosRankSum < −20.0. DbSnp annotation was done against dbSNP build 146 [36] and variant annotation was done with Ensembl’s Variant Effect Predictor v.83 [37] against GRCh38. Expression values were calculated using RSEM with bowtie2. P-values were calculated using Mann–Whitney (one tail) test on the mutations frequencies between the AD subjects and healthy aging subjects.

### Gene expression omnibus (GEO) datamining

**Fig. 1:**
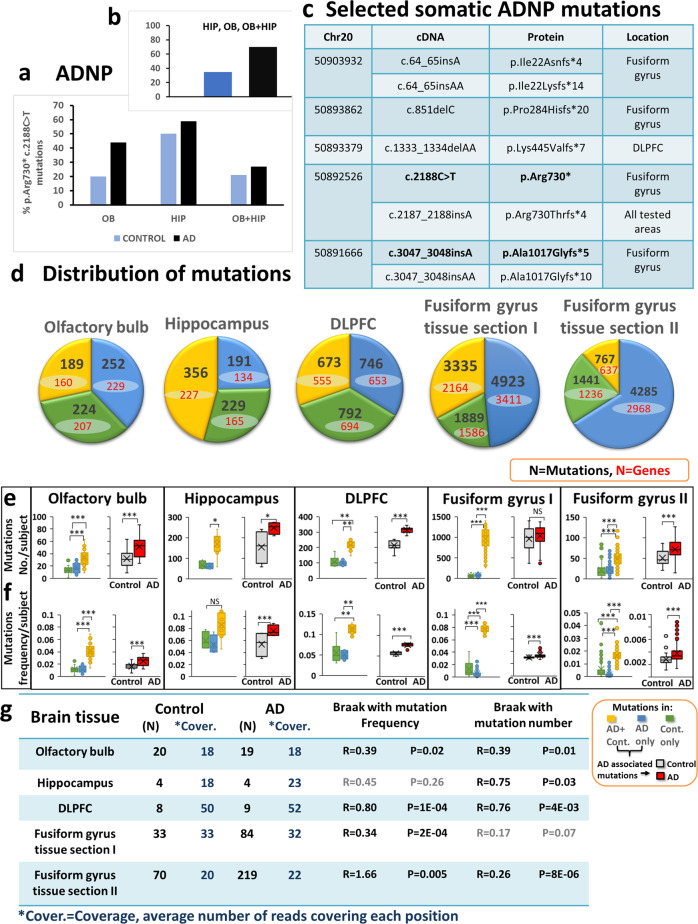
ADNP c.2188C>T, p.Arg730* mutations/AD—multiple somatic mutations in multiple brain areas correlate with tauopathy. **a**, **b** Positive subjects who were found to carry the ADNP c.2188C>T, p.Arg730* mutations and negative cases who were found to be non-carriers. **a** Relative fractions of ADNP c.2188C>T, p.Arg730* mutation positive AD (black) and mutation positive controls (blue) were calculated, in each brain region (Table S1, including 2 borderline positives). OB—olfactory bulb and HIP—Hippocampus, results showed similar distribution in AD and control samples. **b** All mutation positives (OB, HIP, and OB + HIP), excluding borderline positives are shown (14/20; AD; 7/20 controls, Pearson Chi-Square, *P < 0.05). It should be noted that when logistic regression of morbidity as a function of the number of regions with mutations was performed, the result was insignificant. **c** RNA-seq identification of novel, tissue-associated ADNP-AD mutation, autism-related ADNP mutation are marked in bold. **d** Mutation analysis of RNA-seq variant calling of four postmortem brain areas: olfactory bulb (our own primary data), Hippocampus (GSE67333), Dorsolateral prefrontal cortex (DLPFC) (GSE53697) and Fusiform gyrus tissue section I (GSE95587) and Fusiform gyrus tissue section II (GSE GSE125583). The pie charts represent distribution of mutations into three different groups: controls (Cont.) only, AD only and both in AD + controls (AD + Cont.). The Box plots represent (**e**) mutation numbers per subject and (**f**) mutation frequencies per subject for each group in the four brain areas. Statistical analyses (Table S6b) were performed by the nonparametric Kruskal–Wallis test for the hippocampus and DLPFC and ANOVA for the olfactory bulb and the fusiform gyrus with post hoc Bonferroni for multiple comparisons. AD-associated mutations including AD only and AD + Cont. mutations: mutation numbers and mutation frequencies in AD subjects were compered with aging controls. Statistical analyses was done by T-Test for all calculated mutation frequencies and T-Test for olfactory bulb and fusiform gyrus and Mann–Whitney test for hippocampus and DLPFC for number of mutations per subject. Statistical significance is presented by * = *P* < 0.05, ** = *P* < 0.001, *** = *P* < 0.0001. **g** Spearman correlations of mutation numbers per subject and mutation frequencies per subject with Braak stage in control and AD subjects grouped together

Dataset GSE95587 [38] was identified as containing a large cohort (*N* = 117) with a high number of reads covering each position (32) including AD postmortem brain tissues from fusiform gyri section I (supplemental methods), this was accompanied by a larger dataset GSE125583 (*N* = 289) with a lower coverage (20), fusiform gyri tissue section II (Fig. 1). Two additional libraries included GSE67333- hippocampal RNA-seq (late onset AD) [39] and GSE53697-DLPFC (advanced AD) [40]; however, these two libraries should be taken cautiously due to small sample sizes (Fig. 1). For different cell type mutation analysis, the GSE125050 superior frontal gyrus dataset was used including endothelial cells (CD31+), astrocytes (GFAP+) myeloid cells (CD11b+), and neurons (NeuN+) (total sample size 113).

### Plasmid constructions

*ADNP* syndrome mutated cDNA inserts to be cloned into the backbone of the vector pEGFP-C1 were obtained from mRNA extracted from patient-derived lymphoblastoid cell lines carrying the following mutations: c.2188C>T (p.Arg730*), c.2156_2157insA (p.Tyr719*). The insert of full-length human *ADNP* was obtained from a control lymphoblastoid cell line with no mutation [7, 15].

### Cell culture, transfection, plasmid overexpression, and live imaging

Mouse neuroblastoma N1E-115 cells (ATCC, Bethesda, MD) were maintained, differentiated, co-transfection with plasmids expressing EB3-RFP and GFP–ADNP or GFP-mutated-ADNP) and subjected to live imaging [23].

### Fluorescence recovery after photobleaching (FRAP)

Differentiated N1E-115 cells were co-transfected with mCherry-Tau and GFP conjugated to full-length ADNP or mutated ADNP, and imaged 48 h after transfection. An ROI (region of interest) for photobleaching was drawn in the proximal cell branches. mCherry-Tau was bleached with a 587 nm argon laser and fluorescence recovery was at 610–650 nm. Immediately after bleaching, 80 images were collected every 0.74 s. Fluorescence signals were quantified with ImageJ (NIH), obtained data were normalized with easyFRAP [41], and FRAP recovery curves were fitted by a two-phase exponential association function using GraphPad Prism 6 (GraphPad software, Inc., La Jolla, CA). Samples with *R*^2^ < 0.9 were excluded.

### Statistics

Analyses (SPSS 23, Chicago, IL) for postmortem brain tissues employed either ANOVA or Kruskal–Wallis tests (>two groups) followed by nonparametric Mann–Whitney U test. Multiple testing corrections employed Bonferroni. Correlations employed Spearman. Live-cell imaging and FRAP results were analyzed by SigmaPlot 11 (Systat Software, Inc., San Jose, CA) with two-way ANOVA, followed by Fisher LSD.

Additional information can be found in the Supplemental file.

## Results

### Somatic *ADNP* mutations

Given that the prevalence of *ADNP* mutations in autistic children is ~1:500–1:1000 [7], we subjected AD and control postmortem DNA samples to ddPCR and screened for the most abundant ADNP mutations [7, 15]. These included p.Tyr719* (22% of the current ADNP syndrome cases), resulting from different mutations at the cDNA level—e.g. c.2157C>G (7–11%), p.Asn832Lysfs*81 and p.Leu831Ilefs*82 (13–16%), and p.Arg730*—c.2188C>T (6–9%). Surprisingly, mutations were observed only in p.Arg730*, c.2188C>T. The p.Arg730*, c.2188C>T mutation was observed at the DNA level in the postmortem samples from both AD and control subjects, with an insignificantly higher prevalence in AD samples (Fig. 1a, S1-S3, Tables S1, S2). Grouping all tested samples together (including hippocampal and olfactory bulb mutations w/o positive presence of mutations with borderline values) also suggested a 2-fold increase in the AD cases (Fig. 1b).

### Public dataset validation: somatic mutations in multiple genes with AD specificity and enhancement

To extend the single gene findings, postmortem olfactory bulb cDNA samples (19 AD, 20 controls) all presenting tauopathy and ~80% exhibiting amyloid pathology (Table S1) were subjected to RNA-Seq. We then discovered an ADNP c.2187_2188insA, p.Arg730Thrfs*4 mutation (same position as p.Arg730*—c.2188C>T). Results were validated by examination of reads aligned to the area of c.2187_2188insA using Integrative Genomics Viewer (IGV) (Fig. S4).

To extend and validate the findings for ADNP in the olfactory bulbs (Table S3) and the hippocampus to other brain areas we have resorted to datamining of hippocampi, DLPFC and fusiform gyri public RNA-seq databases (Tables S4–S6a,b). As seen in Fig. 1c, the newly discovered mutation appeared in all tested brain regions. Furthermore, a unique mutation appeared in the DLPFC and two additional hot spots were discovered in the fusiform gyrus (c.3047_3048, also seen in ADNP syndrome cases [7] and c.64_65ins). An additional mutation in the fusiform gyri was also seen in c.851delC. Fig. S5 shows predicted hairpin formations by the 100 bp genomic sequence surrounding the prevalent *ADNP* mutations.

In general, in the olfactory bulb we discovered 665 mutations in 596 genes with 441 mutations in AD patients (389 genes, 38% AD—unique mutations, Fig. 1d). When the three groups of mutations (control exclusive, shared and AD unique mutations) were compared the number (Fig. 1e) and frequencies (Fig. 1f) of mutations per subject were significantly higher in the shared group of mutations. Further comparisons of control subjects carrying AD shared mutations to AD subjects carrying shared and unique AD mutations showed highly significant increases in number (Fig. 1e) and frequencies (Fig. 1f) per AD vs. control subjects. Similar results were obtained by RNA-seq datamining of public datasets (hippocampus, DLPFC, and fusiform gyrus I (142 subjects) + an additional cohort of fusiform gyrus II with 89 shared subjects with fusiform gyrus I plus 200 unique subjects, Fig. 1g). (Only the comparison concentrating on AD-mutation number per subject in the fusiform gyrus I were similar in controls and AD subjects). AD mutation numbers and frequencies per subject positively correlated with tauopathy (Braak stage, Fig. 1g and S6 except hippocampi, frequencies and fusiform gyri, numbers). A positive correlation with amyloid beta plaque load was measured in the olfactory bulbs (Fig. S6, *R*~0.4, *P* < 0.05).

**Fig. 2:**
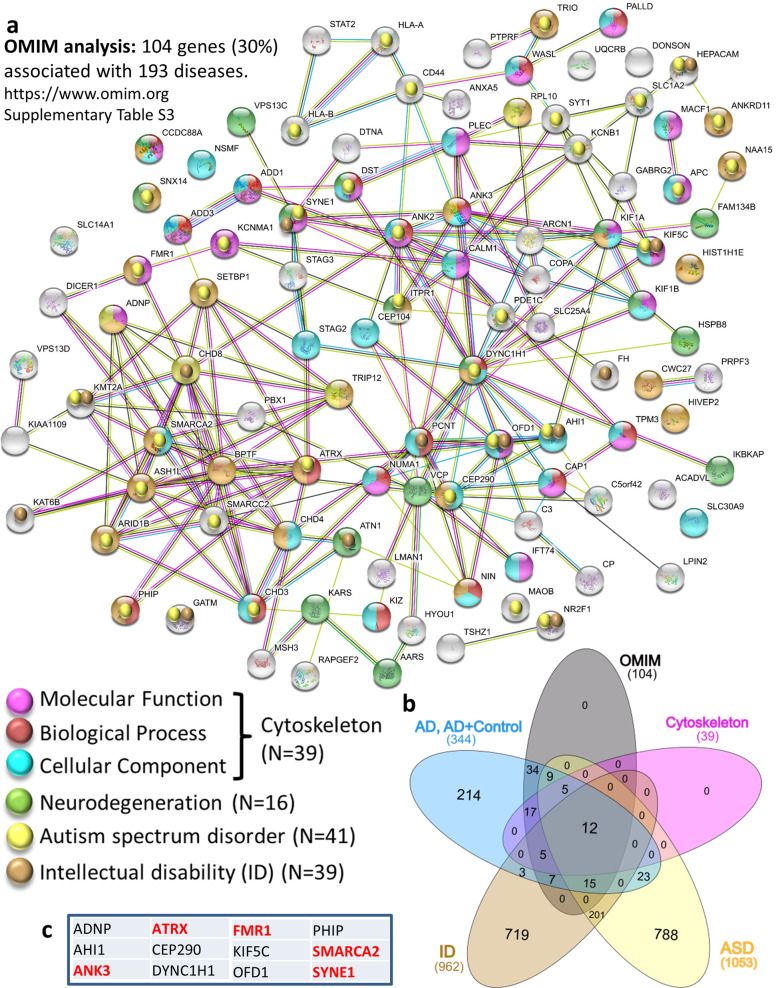
Somatic mutations/AD—networks in the olfactory bulb (OB). **a** Interaction network by the STRING tool for 104 mutated genes with OMIM disease association. The comparisons were made with mutated genes, including AD specific + AD-control shared mutations. Results of variant calling from RNA-Seq from 39 samples of OB indicated GO molecular enrichment-Cytoskeletal protein binding (FDR = 2E-08) colored with light purple, GO Biological process-cytoskeletal enrichment-cytoskeleton organization (FDR = 2.4E-4) colored with red/brown. GO cellular component-cytoskeleton (FDR = 8.9E-8) colored with light blue. A total of 39 genes (38%) related to the cytoskeleton OMIM-identified mutated genes. **b** Venn graph (http://www.interactivenn.net/) identifying genes that are shared with Autism: https://gene.sfari.org/database/human-gene/ and ID: http://www.ccgenomics.cn/IDGenetics/gene.php?dataset=IDGD_gene_detail and http://gfuncpathdb.ucdenver.edu/iddrc/iddrc/data/IDgenelist_gsym.html.**c** Shared genes described in (**b**) are named in table (red, AD—only mutations). Precise description of the genes can be found in Table S10

To correlate mutations to known diseases OMIM analysis (https://www.omim.org) was performed followed by String analysis to identify molecular interactions. Figure 2 shows the olfactory bulb as an example with 104 genes (30%) associated with 193 diseases (Table S3), converging on cytoskeletal mechanisms, autism and ID causing mutations (~40% each, Fig. 2a, b) with 12 gene overlap (Fig. 2b,c, Table S7). Similar enrichments in cytoskeletal mutations were discovered in other tested brain areas specifically in AD-linked mutations (Fig. S7). Interestingly, several DLPFC subjects also showed mutations in genes associated with familial AD (amyloid precursor protein-APP and presenilin 1-PSEN1 (Table S5, Fig. S7).

**Fig. 3:**
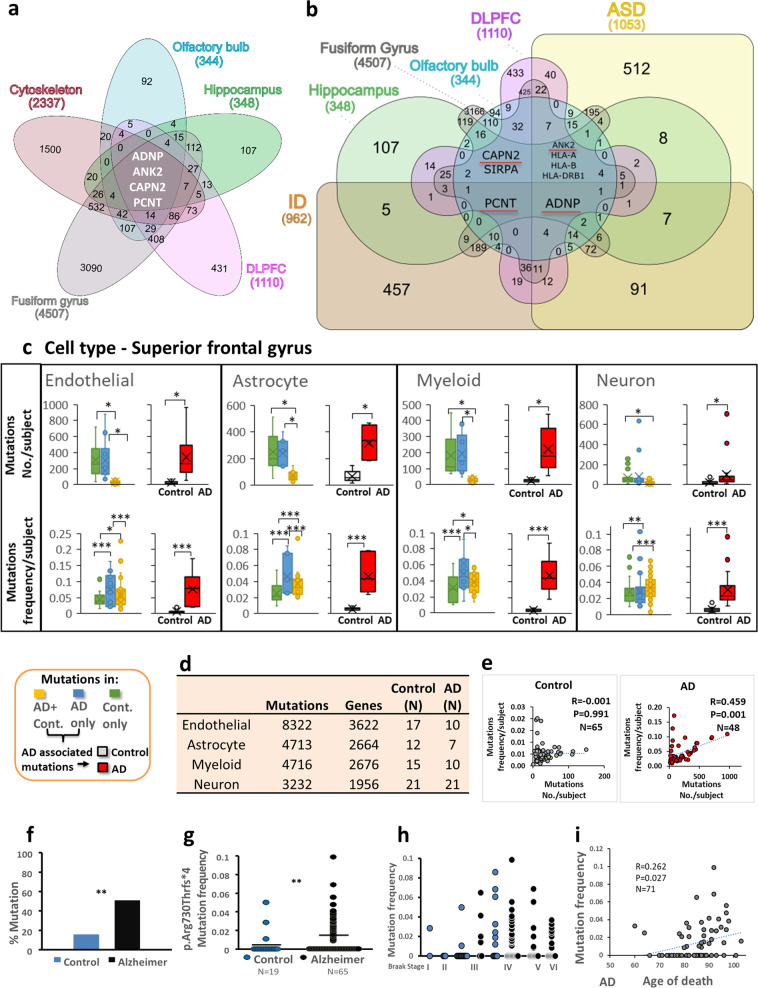
ADNP mutations are shared by all tested brain regions and cytoskeletal proteins/ID/autism, ADNP c.2187_2188insA correlate with AD tauopathy. **a**, **b** Venn graphs (http://www.interactivenn.net/) showing comparisons of mutated genes, including AD specific+ AD-control shared mutations, from four postmortem brain areas: olfactory bulb, Hippocampus (GSE67333), dorsolateral prefrontal cortex (DLPFC) (GSE53697) and fusiform gyrus (GSE95587) shared with **a** cytoskeletal genes (GO:020954 and GO:020801), **b** Autism and ID genes. Note, the numbers of mutated genes are lower than the numbers depicted in Fig. 1d as there are shared genes between AD-specific and AD+ controls shared mutations, however, the mutations/gene are unique in the two different groups. **c** Mutation analysis of RNA-seq variant calling of postmortem superior frontal gyrus from different cell types: Endothelial (CD31+), Astrocyte (GFAP+) Myeloid (CD11B+) and Neuron (NeuN+) (GSE125050). The mutations were devided into three different groups: Controls (Cont.) only, AD only and both in AD+ controls (AD+Cont.). The Box plots represent number of mutations per subject and frequencies of mutations per subject for each group in all cell types. AD-associated mutations including AD only and AD+Cont. mutations: mutation numbers and mutation frequencies in AD subjects were compered with controls. Statistical analyses (Table S12b) were performed by the nonparametric Kruskal–Wallis test for mutation numbers with post hoc Bonferroni for multiple comparisons. For all mutation frequencies ANOVA were performed with post hoc Bonferroni for multiple comparisons. AD-associated mutations including AD only and AD+Cont. mutations: statistical analyses were done by T-Test for all calculated mutation frequencies and for the average mutations number per subject. The Kolmogorov–Smirnov test of normality was used to test normal distribution. Statistical significance is presented by * = *P* < 0.05, ** = *P* < 0.001, *** = *P* < 0.0001. **d** The table represents the distribution of mutations in different cell types. **e** Pearson correlations of mutation numbers per subject with mutation frequencies per subject in control and AD subjects in all cell types grouped together are shown. **f** Percentage of ADNP c.2187_2188insA, p.Arg730Thrfs*4 mutation positive AD (black) and mutation positive controls (blue) in the respective tested populations were calculated showing significantly higher mutation frequency in AD samples (Braak stage IV–VI) compared to age-matched controls (Braak stage I-III) (Pearson Chi-Square, *P* = 0.007). Positive cases are subjects who were found to carry the c.2187_2188insA, p.Arg730Thrfs*4, in the GEO database GSE95587 (postmortem fusiform gyrus tissue sections, see Fig. S8). **g** c.2187_2188insA, p.Arg730Thrfs*4 mutation frequency to the intact ADNP sequence within the cDNA reads, shows a significant differences between controls (blue) and AD (black) samples, one tailed Mann–Whitney Test, *P* = 0.006. **h** ADNP c.2187_2188insA, p.Arg730Thrfs*4 mutation positive showing mutation frequency to intact ADNP (as in g) for all the different Braak stages (control—blue, AD-black) with increased mutation frequency in the control correlated with increased Braak stage, Spearman correlation, *R* = 0.394, *P* = 0.028. **i** Pearson correlation between ADNP mutation ratio and age of death in AD

Overlapping of all identified mutated genes in the four brain areas with cytoskeletal genes (Tables S3–S6a,b S8a) revealed about 25–70% uniquely mutated and four shared genes (Fig. 3a). Overlapping all mutated genes with cytoskeletal genes, while not showing a proportional increase in AD vs. control (Table S8b) did show an AD-specific MT-based processes gene group and a control specific gene group associated with cell projection organization (Fig. S7).

Further overlap with autism spectrum disorder (ASD, autism) database (https://gene.sfari.org/database/human-gene/) and ID database (http://www.ccgenomics.cn/IDGenetics/gene.php?dataset=IDGD_gene_detail) identified unique and shared mutated gene mutations (Tables S9–S11), with ADNP singled out as a gene associated with cytoskeleton/autism/ID/AD (Fig. 3a, b, Table S9).

To better understand the cellular distribution of the mutations, an additional database was mined GSE125050 [42] (Fig. 3c–e, Table S12 a, b). The results per cell mirrored the tissue findings (Fig. 1e, f) with neurons showing the least mutations. Furthermore, mutation frequencies and mutation numbers/subject were significantly correlated only in the AD-derived cells (Fig. 3e).

**Fig. 4:**
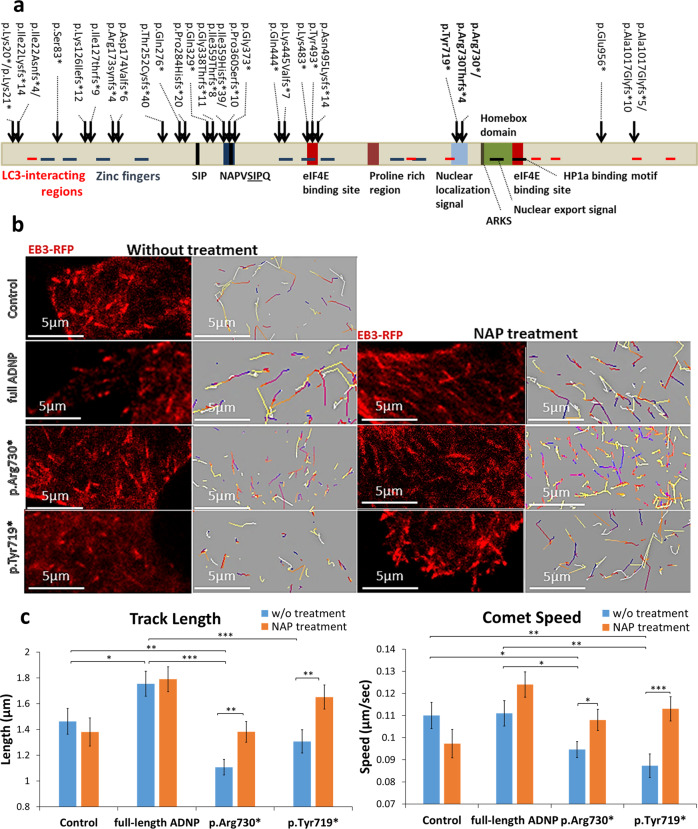
ADNP mutations inhibit MT dynamics, NAP protects. **a** Location of functional protein regions are depicted along full-length human ADNP coding sequence. The arrows point out all ADNP mutations identified in the current study, including the mutations that were examined below. The figure was constructed according previously published data [4–7, 11, 19, 21, 25, 53, 71–75]. **b** Live imaging of N1E-115 cells expressing EB3-RFP with GFP conjugated to full-length ADNP or truncated ADNP proteins with or without NAP treatment (10^−12^M) for 4 h. Transfection with backbone plasmid (pEGFP-C1) expressing non-conjugated GFP, was performed as a control. Time-lapse images were automatically captured every 3 s during a 1 min using the Leica LAS AF software. Tracks of EB3 comet-like structures presented as colored lines and were obtained by the Imaris software. **c** Graphs represent quantification of the average track length and comet speed. Data from three independent experiments were collected in unbiased fashion by the Imaris software, and statistical analysis (Table S14) of the data was performed by Two-Way ANOVA (SigmaPlot11). Statistical significance is presented by *P < 0.05, **P < 0.01, ***P < 0.001. Control *n* = 26; Control + NAP *n* = 11; full-length ADNP *n* = 28; full-length ADNP + NAP *n* = 28; p.Arg730* *n* = 43; p.Arg730* + NAP *n* = 20; p.Tyr719* *n* = 32; p.Tyr719* + NAP *n* = 30

IGV analysis of the large GSE95587 (fusiform gyrus I) cohort with high sequencing coverage (Fig. 1g, Fig. S8) revealed significantly more c.2187_2188insA *ADNP* mutation carriers (~50%) among the AD subjects than controls (15%, Fig. 3f, Table S6a, bold). Figure 3g shows the frequency of the c.2187_2188insA, mutation to the intact *ADNP* sequence within the cDNA reads. The findings indicated up to ~10% mutated forms, suggestive of somatic mutations. Comparison of controls (Braak stage I–III) and AD (Braak stage IV–VI) revealed a significant enrichment of *ADNP* mutations in AD subjects (Fig. 3h). Subjects displaying postmortem Braak stage IV (AD and controls) showed a similar frequency of the *ADNP* c.2187_2188insA, mutation. Furthermore, a significant correlation between the *ADNP* c.2187_2188insA, mutation and the Braak stage was observed in control subjects (*R* = 0.394, Spearman correlation, *p* = 0.028, *n* = 31, Braak stage I–IV), Fig. 3h. Correlation was also discovered between *ADNP* mutation frequency and the age of death (Fig. 3i). Furthermore, ADNP mutations were more prevalent in AD specimens compared to controls (Table S6a, ADNP). Interestingly, our study revealed multiple *ADNP* somatic mutations as outlined in Fig. 1c and extended to include additional cellular mutations (Fig. 3c–e, Table S13, Fig. 4a).

### ADNP mutation induced tauopathy corrected by NAP

Given the prevalence of *ADNP* somatic mutations in postmortem AD brains and the potential convergence of identified gene mutations on the cytoskeleton, we hypothesized cytoskeletal damage as a consequence of ADNP mutations. To test our hypothesis, we imaged MT dynamics in living N1E-115 neuroblastoma cells, while tracking the growth of individual MTs with RFP-tagged EB3 proteins that bind to MT plus-ends. Two parameters of EB3 mobility were used to assess MT dynamics: growth track length, and growth track speed of EB3 comet-like structures. These parameters reflect the lengths of the MT growing events and the speed of MT assembly, respectively [23]. In addition, we examined whether incubation with the MT-interacting ADNP snippet, NAP (10^−12^M, 4 h) could protect against the deleterious ADNP mutations.

Figure 4a–c (Figs. S9–S11, movies S1–S2) show that overexpression of full-length ADNP significantly increased EB3 comet track length and that NAP treatment did not further influence this increase. Expression of the ADNP p.Arg730*, or p.Tyr719* mutations (Fig. 4a–c, Table S14, Movies S3–S4) significantly decreased EB3 comet speeds and track lengths, compared to either full-length ADNP or control plasmid (except for ADNP p.Tyr719*, where the reduction in EB3 track length was significant compared only to full-length ADNP). NAP treatment significantly augmented the growth speed and track length of the EB3 comets with both mutations.

**Fig. 5:**
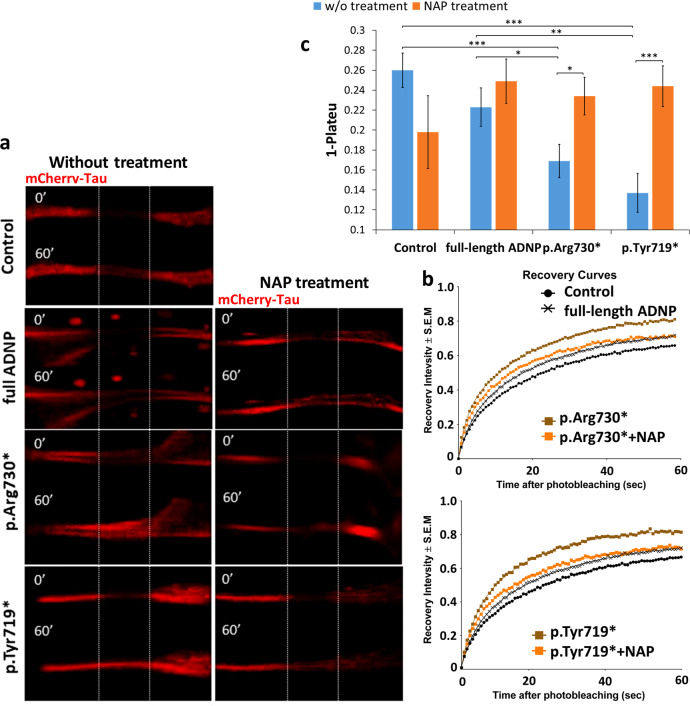
ADNP mutations destabilize Tau–MT interactions, NAP protects. **a** Representative images of photobleaching and fluorescence recovery of mCherry-Tau in differentiated N1E-115 cells co-transfected with plasmids expressing mCherry-Tau and GFP conjugated to full-length ADNP or truncated ADNP proteins with or without NAP treatment (10^−12^M) for 4 h. Transfection with backbone plasmid (pEGFP-C1) expressing non-conjugated GFP, was performed as a control. **b** FRAP recovery curves of normalized data. **c** Graph represents averages of the fitted data of immobile fractions (from three independent experiments). Normalized FRAP data were fitted with two-exponential functions (GraphPad Prism 6) and statistical analyses (Table S14) were done by Two-Way ANOVA (SigmaPlot 11). Statistical significance is presented by *P < 0.05; **P < 0.01;*** *P* < 0.001. Control *n* = 45; Control + NAP *n* = 10; full-length ADNP *n* = 36; full-length ADNP + NAP *n* = 47; p.Arg730* *n* = 47; p.Arg730* + NAP *n* = 37; p.Tyr719* *n* = 35; p.Tyr719* + NAP *n* = 42

To examine the effect of full-length/truncated ADNP forms and NAP on Tau–MT interactions, we used FRAP (Fig. 5). The intensity of mCherry-tagged Tau protein fluorescence recovery within a photo-bleached region of interest (ROI) reflects the immobile fractions of bleached molecules, which do not release binding sites on MTs for incoming un-bleached mCherry-Tau proteins and thus do not contribute to the fluorescence recovery. Consequently, the immobile mCherry-Tau fraction reflects the rate of Tau association with MTs [43].

We observed that overexpression of full-length ADNP resulted in a minor insignificant reduction in the immobile mCherry-Tau fraction compared to control (Fig. 5a–c), which was not affected by NAP treatment (Fig. 5a, c, Table S14). Importantly, both truncated forms of ADNP significantly attenuated Tau association with MTs, compared to the GFP-control, or full-length ADNP (Fig. 5a–c). NAP treatment restored the Tau–MT interaction to control values (GFP and full-length ADNP controls, Fig. 5a–c). Finally, the ability of NAP to protect MTs against degradation by promoting Tau–MT interaction has been previously shown in the murine cell lines [23, 25] and confirmed here (Supplemental results, Figs. S12–S13), in a human neuroblastoma SH-SY5Y cell model.

## Discussion

The amyloid hypothesis domineers the framework for AD research and therapies, despite continuous clinical failures (https://www.alzforum.org/news/research-news/biogeneisai-halt-phase-3-aducanumab-trials). Tau-clearance therapies [15] are also being developed, however, Tau monotherapy may not suffice [44, 45]. Importantly, it is apparent that the various tauopathies constitute different diseases, which may require versatile treatment modalities [15]. For example, NAP (davunetide, AL-108) failed in the pure 4R tauopathy progressive supranuclear palsy (PSP) [46], but showed efficacy in increasing cognitive scores in patients with amnestic mild cognitive impairment (mixed 3R + 4R tauopathy) [15, 47] involving differential interaction with Tau 3R/4R splice variants [28].

Mutations in cytoskeletal proteins may contribute only partly to drug targeting in AD precipitation, as the plethora of somatic pathogenic mutations may have cumulative effects. An intriguing question is how these mutations arise. One possibility is dysregulation of DNA proofreading, excision or single strand repair in the face of environmental stress coupled to random mutation accumulation during cell divisions. While DNA repair protein mutations (http://www.informatics.jax.org/go/term/GO:0006281) did not appear as a major group of gene mutations in our OMIM analyses, further tests discovered multiple DNA repair protein mutations with shared and unique brain distribution (Table S15, fusiform gyrus showing 2-fold higher AD mutation number vs. control, paralleling doubled number of AD subjects, Fig. 1). Mutations included excision associated repair genes (ERCC1, XPA = ERCC2, ERCC3, and ERCC5) only in the AD-associated fusiform gyrus (Table S15, and Supplemental Discussion), rendering higher sensitivity to radiation hypersensitive neurodegeneration [48, 49]. Similarly, lymphoblastoid cells from AD patients are more sensitive to irradiation damage [49]. Although central and peripheral mutated genes may differ, we have found correlations between lymphoblastoid/blood and AD brain gene expression [50, 51]. Furthermore, nucleotide excision repair is modulated by the mammalian SWI/SNF chromatin-remodeling complex [52], ADNP constitutes a part of this complex [53], and blood ADNP levels correlate with AD [20].

Regarding cell division and potential accumulation of mutations (paralleling with de novo mutations in autism), adult neurogenesis drops sharply in the AD hippocampus [2]; however, glial cells divide and may contribute to the neuroprotective/neurodegenerative process. We now show increased mutations in non-neuronal brain cells compared to neurons. We singled out ADNP (a neuro-glial protein) [4] as a case study of cytoskeleton/autism/ID/AD shared gene. In searching for leading gene candidates for autism, others ranked ADNP as a lead, paralleling our findings regarding AD potentially mutated genes [8]. Our results suggest that with aging and AD, the most significant finding in ADNP is the p.Arg730*/p.Arg730Thrfs*4 pathogenic mutations, which occur presumably somatically in selected cells, with ADNP tightly linked to cytoskeletal regulation and dendritic spine plasticity [14, 23, 25, 29].

Regarding the cytoskeletal mutations and disease propagation, injection of human soluble Tau into the mouse dentate gyrus resulted in markedly reduced synapse numbers in the hippocampal molecular layer. Soluble tau damages the morphology and connectivity of newborn granule cells [54] and NAP/ADNP enhance Tau–MT association and dendritic spine formation in an EB1/EB3-dependent manner [23, 25]. Furthermore, Tau aggregation is inhibited by augmented autophagy [55], and ADNP/NAP accelerate autophagy [14, 15, 19, 56–59]. As tauopathy is suggested to propagate in a prion-like manner [60–62], it is hypothesized that even rare occurrences of cellular tauopathy will propagate, making prevention therapy with NAP treatment prior to disease onset a desired possibility. More generally, recent findings show that the retrovirus-like Gag protein Arc1 binds RNA and traffics across synaptic buttons, suggesting the possibility of wide transfer of mutated disease driving RNAs [63]. The discovery of somatic mutations in genes causing familial, amyloid-driven AD [64–66] also in our cohorts (DLPFC, Table S5, Fig. S7) provides increased relevance to our findings.

Our study mostly relies on RNA-seq data (partially backed by sensitive ddPCR). A recent RNA-seq publication elegantly supports our findings revealing macroscopic somatic clonal expansion across normal tissues. Furthermore, this cited study finds age-dependent increases in clonal expansion, in agreement with our multiple mutation discoveries in the elderly control cohorts [67]. Together, our studies corroborate RNA-seq results in terms of mutation analysis [67] and further imply that accumulating mutations with aging may contribute to the increased risk of AD, with aging being the highest risk factor for AD.

Regarding prevention by NAP and related molecules [14], our data suggested that ADNP enhanced MT dynamics with a saturation effect, explaining previously observed in vitro bell-shape dose dependence for NAP, albeit, over a very broad concentrations range [4]. Our model here for AD protection included overexpression of a mutated ADNP in a cell expressing intact ADNP, suggesting antagonistic function and competition with the endogenous ADNP over MT association and enhancement of MT dynamics, which is ameliorated by NAP treatment. Indeed, our previous studies showed that NAP enhances intact ADNP association with MTs [25] and protects against *Adnp*^*-*^ haploinsuffiency in mice [13]. These results pave the path to NAP/ADNP enhancing therapy in the ADNP syndrome (CP201, Coronis Neurosciences) [68] and for NAP and related molecules as preventive treatment in prodromal AD [69].

In conclusion, we revealed somatic aging/AD-linked mutations converging on tauopathy [70], including NAP/ADNP [25]. We further showed a significant correlation between the frequency of the *ADNP* c.2187_2188insA mutation and aging, suggesting accumulation with aging and increasing Braak stages, implicating either a parallel or a causal relation, possibly linked to Tau-like prion-like propagation [60–62]. Together, our results represent a paradigm-shifting concept in the perception of AD, whereby accumulating somatic gene mutations promote brain pathology and cognitive loss, and open new horizons for research and development.

## Supplementary information

Supplemental Materials

Supplemental Figure S7

Movie S1

Movie S2A

Movie S2B

Movie S3A

Movie S3B

Movie S4A

Movie S4B

Supplemental Table S2

Supplemental Table S3

Supplemental Table S4

Supplemental Table S5

Supplemental Table S6a

Supplemental Table S7

Supplemental Table S8a

Supplemental Table S8b

Supplemental Table S9

Supplemental Table S10

Supplemental Table S12a

Supplemental Table S15

## Data Availability

The datasets generated and data analyzed during the current study have been deposited in the NCBI Gene Expression Omnibus and are accessible through GEO Series accession number GSE113524.
