# Supplementary material for: Discovery of autism/intellectual disability somatic mutations in Alzheimer's brains: mutated ADNP cytoskeletal impairments and repair as a case study
Source: Mol Psychiatry. 2019 Oct 30;26(5):1619–33. doi: 10.1038/s41380-019-0563-5 (PMC8159740; doi:10.1038/s41380-019-0563-5)
Supplement: Supplementary file 1 — Supplemental Materials [file 41380_2019_563_MOESM1_ESM.pdf]

Supplemental Material:

Discovery of Autism/Intellectual Disability Somatic Mutations in Alzheimer's Brains: Mutated ADNP Cytoskeletal Impairments and Repair as a Case Study

Yanina Ivashko-Pachima[1*], Adva Hadar[1*], Iris Grigg[1], Vlasta Korenková[2], Oxana Kapitansky[1], Gidon Karmon[1], Michael Gershovits[3], C. Laura Sayas[4], R. Frank Kooy[5], Johannes Attems[6], David Gurwitz[1], Illana Gozes[1]**

*Contributed equally

[1]The Lily and Avraham Gildor Chair for the Investigation of Growth Factors; The Elton Laboratory for Neuroendocrinology; Department of Human Molecular Genetics and Biochemistry, Sackler Faculty of Medicine, Sagol School of Neuroscience and Adams Super Center for Brain Studies, Tel Aviv University, Tel Aviv 69978, Israel; [2] BIOCEV, Institute of Biotechnology CAS, Průmyslová 595, 252 50 Vestec, Czech Republic; [2]The Nancy & Stephen Grand Israel National Center for Personalized Medicine, Weizmann Institute of Science, Rehovot, Israel; [4]Centre for Biomedical Research of the Canary Islands (CIBICAN), Institute for Biomedical Technologies (ITB), Universidad de La Laguna (ULL), Tenerife, Spain; [5]Department of Medical Genetics, University of Antwerp, Antwerp, Belgium; [6]Institute of Neuroscience and Newcastle University Institute of Ageing, Newcastle University, Newcastle upon Tyne, UK.

**Corresponding author:

Illana Gozes, Ph.D.; Professor of Clinical Biochemistry

The Lily and Avraham Gildor Chair for the Investigation of Growth Factors

Head, the Dr. Diana and Zelman Elton (Elbaum) Laboratory for Molecular Neuroendocrinology

Sackler Faculty of Medicine, Tel Aviv University

Tel Aviv 69978, Israel, Phone: 972-3-640-7240, Fax: 972-3-640-8541

E-mail: igozes@post.tau.ac.il

Supplemental Methods

Droplet digital PCR

Droplet digital PCR (ddPCR) enables precise, highly sensitive quantification of nucleic acids. The DNA sample is fractionated into about 20,000 droplets, and PCR amplification of the template molecules occurs in each individual droplet. After amplification, droplets containing target sequence are detected by fluorescence and scored as positive, while droplets without fluorescence are scored as negative. Two targets can be detected simultaneously. Poisson statistical analysis of the numbers of positive and negative droplets yields an absolute quantification of the target sequence.

To estimate the rate of false positives (FPR), control samples were analyzed as olfactory bulb (OB) and hippocampus (H) samples, respectively. The FPR of OB control samples was measured in samples 1, 2, 3, 4, 6, 8, 9, 10, 22, 24, 26, 27, 28, 30, 38, 39, and 40, with the FPR (OB) estimated as 0.019%. The FPR of H control samples was measured in samples 1, 3, 4, 5, 22, 24, 26, 27, 28, 30, 36, 38, 39, and 40, with the FPR (H) estimated as 0.019%. NTC controls (no template control) contained no DNA. A pool of NTC derived from 5 ddPCR reactions, did not contain any positive droplets.

The sensitivity was validated by preparing three-fold serial dilutions from positive controls (serial dilutions of gBlocks® Gene Fragments for c.2188C>T mutation, purchased from Integrated DNA Technologies (IDT), Inc. Skokie, IL, USA) and ADNP in control 7 DNA sample that was used as a diluent (Supplemental Table S1). Preparations were designed to achieve theoretical mutant/control percentages of 1% to 0.01%, although the experimentally corrected percentages, (termed fractional abundance (FA)), actually started from 0.75% (Supplemental Fig. S1). The limit of detection (LOD) was established as the reciprocal of the highest dilution at which all replicates were still positive[2]. A positive measurement was defined as a reaction containing at least three positive droplets for the sequence of interest (mutation) in at least 7000 WT positive background droplets in a single ddPCR reaction.

Gene expression omnibus (GEO) data mining (IGV) Software

The GEO NCBI website was screened for RNA-seq datasets derived from human AD postmortem brain tissues. GEO files were downloaded using the Galaxy platform[3] "Download and Extract Reads in FASTQ format from NCBI SRA" (Galaxy Version 2.8.1.3)[4]. ADNP mutations were analyzed by mapping reads to the human genome (Ensembl GRCh38) using "RNA STAR Gapped-read mapper for RNA-seq data" (Galaxy Version 2.5.2b-0)[5]. Reads aligned to ADNP were visualized and examined by Integrative Genomics Viewer (IGV) Software. The mutation frequency for ADNP for each subject was defined as the number of reads with a mutation compared to the total number of reads. Genome wide analysis of the public dataset was done as follows: reads were preprocessed and mapped to the human genome (GRCh38) as already described in the variant calling methods, with STAR in two Pass mode, using Ensembl annotation v.83. The reads were then deduplicated using Picard Tools v. 1.102. Further processing steps, variant calling, filtration, and annotation were as described above. GO-term analysis with the STRING database[6] network was performed on all datasets.

Plasmid constructions

Unique *ADNP* mutated forms [c.2188C>T (p.Arg730*), c.2156_2157insA (p.Tyr719*)[7]] and full-length *ADNP* were cloned into the backbone of the vector pEGFP-C1. After cloning, the insert regions were sequenced to validate the in-frame insertion, and the presence of expected mutations that form premature stop codon. Expression of the full-length ADNP and ADNP truncated proteins was verified by western blot analysis and fluorescence imaging (please see supplemental result section).

Statistical analysis

SigmaPlot 11 (Systat Software, Inc., San Jose, CA, USA) was used to analyze the data by two-way ANOVA, followed by the Fisher LSD *post hoc* test.

Human cell culture and differentiation

Human neuroblastoma SH-SYS5 cells (ECACC, Public Health England, Porton Down, Salisbury, UK; passage numbers from 14 to 16, mycoplasma clean) were maintained in

Ham's F12: minimum essential media (MEM) Eagle (1:1), 2mM Glutamine, 1% non-essential amino acids, 15% fetal bovine serum (FBS) and 100 U/ml penicillin, 100 mg/ml streptomycin (Biological Industries, Beit Haemek, Israel). SH-SY5Y cells were plated in 6-well plates or 10cm dishes at a concentration of $25*10^3$ cells/well or $0.5*10^6$ cells/dish, accordingly, and differentiated during seven days with retinoic acid at a concentration of 10 µM. The cells were incubated in 95% air/5% CO_2 in a humidified incubator at 37°C.

Polymerized vs. soluble tubulin assay

To quantify tubulin polymerization, a polymerized vs. soluble tubulin assay was used as described previously[8-10]. On the day of experiment, differentiated SH-SY5Y cells were treated for 4 hrs with zinc chloride (ZnCl2; final concentration, 400 µM, Sigma, Rehovot, Israel) with or without NAP addition (10^{-12}M). Soluble (S) tubulin was harvested with MT TritonX-100-buffer (80 mM PIPES pH 6.8, 1 mM MgCl2, 2 mM EGTA, 5% Glycerol, 0.5% TritonX-100) through centrifugation (800rcf) at room temperature for 5 min. Pelleted cells were rinsed once again with equal volume of modified RIPA buffer (50 mM Tris-HCL pH 7.4, 150 mM NaCl, 2 mM EGTA, 1% Triton X-100, 0.1% SDS, 0.1% sodium Deoxycholate) in order to collect the polymerized (P) tubulin. The soluble and polymerized fractions were each mixed with sample buffer (final concentrations10mMTris–HCl, pH6.8, 1.5% SDS, 0.6% DTT and 6% (v/v) glycerol) and heated at 95°C for 5min. An equal volume of each fraction was analyzed by immunoblotting with Tau (Tau5, MBL, Woburn, MA, USA), tubulin (T6199, Sigma, Rehovot, Israel) and actin (A2228, Sigma, Rehovot, Israel) antibodies. Secondary antibodies were goat anti-mouse-horseradish peroxidase - HRP (Jackson ImmunoResearch, West Grove, PA, USA).

Immunoprecipitation assay (IP)

Proteins were extracted from differentiated SH-SY5Y cells with lysis buffer (Pierce, Rockford, IL) with added protease inhibitor (11255500, Roche, Mannheim, Germany). Immunoprecipitation (IP) was performed with tubulin antibodies (Tub2.1 identifying neuronal-enriched tubulin[11]). Protein lysate was incubated with tubulin antibodies for 24 hrs at +4°C; 2.3µg of NAP, diluted into lysis buffer (NAP 2.3µg/sample), or the equal volume of lysis buffer w/o NAP were added together with agarose beads for additional 4

hrs at +4°C under constant mixing. Flow-through, wash 1 and 3, and elution fractions were collected and analyzed by immunoblotting with tubulin (T6199, Sigma, Rehovot, Israel) and Tau (Tau5, MBL, Woburn, MA, USA) antibodies. Secondary antibodies were goat anti-mouse-horseradish peroxidase - HRP (Jackson ImmunoResearch, West Grove, PA, USA). Please see Supplemental Results.

Supplemental Results:

<u>**List of Supplemental Tables:**</u>

Table S1: Demographics, Alzheimer's disease (AD) pathology, ADNP c.2188 C>T (p.Arg730*) (ddPCR) and c.2187_2188insA, p.Arg730Thrfs*4 (RNA-Seq) mutation rates in postmortem brains of elderly subjects.

Table S2: Raw data for the ddPCR studies.

Table S3: Raw data for the RNA-seq, olfactory bulb, experimental cohort.

Table S4: Hippocampus RNA-seq mutations GSE67333.

Table S5: Dorsolateral prefrontal cortex (DLPFC) RNA-seq mutations GSE53697.

Table S6a: Fusiform gyrus tissue section I RNA-seq mutations GSE95587 and Fusiform gyrus tissue section II GSE125583.

Table S6b: Statistical analysis of mutation frequencies and number per subject in the different brain areas.

Table S7: Autism (ASD), ID, cytoskeleton, disease-driving genes are mutated in the olfactory bulb of elderly tauopathy controls and AD subjects.

Table S8a: Shared disease-driving gene mutations with cytoskeleton genes (GO:020954 and GO:020801) in all tested brain areas, including olfactory bulb, hippocampus, dorsolateral prefrontal cortex (DLPFC) and fusiform gyrus.

Table S8b: All shared gene mutations with cytoskeleton genes (GO:020954 and GO:020801) in all tested brain areas, including olfactory bulb, hippocampus, dorsolateral prefrontal cortex (DLPFC) and fusiform gyrus.

Table S9: Shared disease-driving genes mutations with Autism (ASD) and ID genes in all tested brain areas, including olfactory bulb, hippocampus, dorsolateral prefrontal cortex (DLPFC) and fusiform gyrus.

Table S10: In depth mutation description of shared disease-driving genes with Autism (ASD) and ID genes in all tested brain areas, including olfactory bulb, hippocampus, dorsolateral prefrontal cortex (DLPFC) and fusiform gyrus.

Table S11: OMIM in depth description: Autism (ASD), ID, cytoskeleton, disease-driving genes are mutated in the olfactory bulb of elderly tauopathy controls and AD subjects.

Table S12a: RNA-seq from different cell types in the superior frontal gyrus GSE125050.

Table S12b: Statistical analysis of different mutation frequencies and number per subject in different cell types**.**

Table S13: Specific cell type enriched ADNP mutations.

Table S14: Statistical analysis of cell culture results.

Table S15: Shared potential disease-driving genes mutations with DNA repair genes: GO:0006281.

List of Supplemental Figures:

Figure S1: Serial dilution of the c.2188C>T mutation control (gBlock), (ADNP p.Arg730*) in a constant control 7 (Supplemental Table S1) background.

Figure S2 (a-c): 1D amplitude & 2D amplitude dot plots for the ADNP c.2188C>T mutation (positive sample gBlock) in a constant WT background.

Figure S3 (a-c): 1D amplitude & 2D amplitude dot plots for the ADNP c.2157C>G mutation (positive sample gBlock) in a constant WT background.

Figure S4: RNA-seq results for p.Arg730Thrfs*4 ADNP mutation visualized in Integrative Genomics Viewer (IGV).

Figure S5: Predicted hairpin formation by 100bp genomic sequence surrounding ADNP mutations.

Figure S6: Spearman correlations of mutations number per subject and mutations frequency per subject with Braak stage or amyloid beta load in control and AD subjects.

Figure S7: String analysis of interacting mutated proteins.

Figure S8: Data mining of RNA-Seq results from postmortem fusiform gyrus tissue sections. Numbers of individual subjects are depicted**.**

Figure S9: Plasmid maps.

Figure S10: Verification of ADNP truncated protein expression by Western blotting.

Figure S11: Verification of plasmid expression by fluorescent imaging.

Figure S12: NAP increases Tau-MT interactions and MT polymerization upon zinc intoxication in the human neuroblastoma cells.

Figure S13: NAP enhances Tau-tubulin association in human neuroblastoma cell model.

Table S1: A. Control subjects

#	Age	Sex	Last MMSE	APOE	Braak	Aβ phase[12]	ddPCR		RNA- Seq
							Hippocampus	Olfactory bulb	Olfactory bulb
**1**	86	M	28		4	4	✗	✗	++
**2**	84	M	-		2	3	-	✗	++
**3**	71	M	-		1	3	✗	✗	++
**4**	89	F	-	E3E3	3	2	✗	✗	++
**5**	85	M	30		1	2	✓	✓	✗
**6**	88	M	30		1	1	-	✗	++
**7**	96	F	-	E3E4	3	0	-	✓	++
**8**	99	F	27		2	0	-	✗	++
**9**	80	M	29		2	0	-	✗	✗
**10**	64	M	-		2	-	-	✗	++
**22**	65	F	-		1	-	✗	✗	✗
**24**	101	F	28		4	5	✓	✓	✗
**26**	96	M	28	E3E4	2	4	✓	✗	++
**27**	81	F	-		2	4	✗	✗	++
**28**	88	M	-		2	3	✓	✗	++

**30**	97	F	-	E3E4	2	2	✓*	✗	++
**36**	93	F	-		3	1	✓	✓	++
**38**	94	M	-		2	1	✓	✗	++
**39**	91	M	-		3	0	✗	✗	++
**40**	81	F	-		1	0	✗	✗	++

B. AD subjects

#	Age	Sex	Last MMSE	APOE	Braak	Aβ phase[12]	Disease Onset	ddPCR		RNAseq
								Hippocampus	Olfactory bulb	Olfactory bulb
**11**	86	F	0		6	5	71	✗	✓	✗
**12**	92	F	0		6	5	89	✗	✗	++
**13**	81	M	0		6	5	69	✗	✗	++
**14**	76	M	5	E3E4	6	5	69	✓	✗	++
**15**	87	M	10		6	5	78	✓	✗	✗
**16**	95	F	0		6	5	81	✓	✓	✗
**17**	82	M	9		6	5	72	✓	✗	++
**18**	90	M	16		6	5	87	-	✗	++
**19**	80	M	12	E3E3	6	4	73	-	✓	++

**20**	88	M	15		4	4	80	-	✗	++
**21**	68	M	12	E2E3	6	5	62	✗	✗	✗
**23**	78	F	2	E3E4	6	5	58	✗	-	-
**25**	76	M	-	E4E4	6	5	71	✗	✓	++
**29**	96	F	0		6	5	91	✓	✓	++
**31**	58	F	7		6	5	53	✓	✓*	✗
**32**	71	M	0		6	5	60	✓	-	✗
**33**	96	M	13		5	5	93	✓	✗	++
**34**	85	M	19		6	4	83	✓	✓	✗
**35**	87	M	6		4	4	80	✗	✓	++
**37**	95	M	20	E3E3	3	3	92	✓	✗	++

Table S1: Demographics, Alzheimer's disease (AD) pathology, ADNP c.2188 C>T (p.Arg730*) (ddPCR) and c.2187_2188insA, p.Arg730Thrfs*4 (RNA-Seq) mutation rates in postmortem brains of elderly subjects**.** The table shows mutational rates and demographic data for controls and Alzheimer's postmortem brain tissue (olfactory bulb and hippocampus). The ddPCR results are indicated as positive with symbol ✓. The ✓* indicates positive presence of mutation with borderline values. The minus symbol (-) indicates "not tested". The (✗) indicates a negative result. RNAseq results are indicated as positive symbol ++. The minus symbol (-) indicates "not tested". The (✗) indicates a negative result. Please see below details assay conditions. For more details, please see Supplemental Tables S2 and S3.

ddPCR™ Assays and sequences (Bio-Rad)[13]:

1. c.2188C>T unique assay ID: dHsaMDS971559989 (ADNP p.Arg730*T)

MIQE Context Sequences:

hg19|chr20:49509002-49509124:

TCCAAGTCTGGCACCTGTGAAGCGCACTTACGAGCAAATGGAATTTCCCTTAC
TGAAAAAA[C/T]GAAAGTTAGATGATGATAGTGATTCACCCAGCTTCTTTGAA
GAGAAGCCTGAAGAGCCTGT

2. c.2157C>G unique assay ID: dHsaMDS423261257 (ADNP p.Tyr719*)

MIQE Context Sequences:

hg19|chr20:49509033-49509155:-

AGACAAATGCACCCTCTCGGCTTAATCAGTCTCCAAGTCTGGCACCTGTGAA
GCGCACTTA[C/G]GAGCAAATGGAATTTCCCTTACTGAAAAAACGAAAGTTAG
ATGATGATAGTGATTCACCCA

3. gBlock for c.2188C>T:

CCCATGAGACATACAAAAAGGTAATGCCGCCTCGCTAGGTGAGCTACAGCTC
GATTGTCACGTTAAGCTGGCCTATACCAGCAACATGACCGCCTCAACTATCA
CTCTGCATCTAGTTCACTGCAGGGGCGTTGGAAAGACCCAAAATGGCCAGGA
TAAGACAAATGCACCCTCTCGGCTTAATCAGTCTCCAAGTCTGGCACCTGTGA
AGCGCACTTACGAGCAAATGGAATTTCCCTTACTGAAAAAA[T]GAAAGTTAG
ATGATGATAGTGATTCACCCAGCTTCTTTGAAGAGAAGCCTGAAGAGCCTGT
TGTTTTAGCTTTAGACCCCAAGGGTCATGAAGATGATTCCTATGAAGCCAGG
AAAAGCTTTCTAACAAAGTATTTCAACAAACAGCCCTATCCCACCAGGAGAG
AAATTGAGAAGCTAGCAGCCAGTTTATGGTTATGGAAGAGTGACATCGCTTC
CCAGGTCTCGACTATACGCCCGTTTTCGGATC

4. gBlock for c.2157C>G:

CCCATGAGACATACAAAAAGGTAATGCCGCCTCGCTAGGTGAGCTACAGCTC
GATTGTCACGTTAAGCTGGCCCACCTACAAATGTATCCATTGCCTTGGTGTGT
ATACCAGCAACATGACCGCCTCAACTATCACTCTGCATCTAGTTCACTGCAGG
GGCGTTGGAAAGACCCAAAATGGCCAGGATAAGACAAATGCACCCTCTCGG
CTTAATCAGTCTCCAAGTCTGGCACCTGTGAAGCGCACTTA[G]GAGCAAATG
GAATTTCCCTTACTGAAAAAACGAAAGTTAGATGATGATAGTGATTCACCCA

GCTTCTTTGAAGAGAAGCCTGAAGAGCCTGTTGTTTTAGCTTTAGACCCCAAG
GGTCATGAAGATGATTCCTATGAAGCCAGGAAAAGCTTTCTAACAAAGTATT
TCAACAAACAGCCCTATCCCACCAGGAGAGAAATTGAGAAGCTAGCAGCCA
GTTGGTCTCGACTATACGCCCGTTTTCGGATC

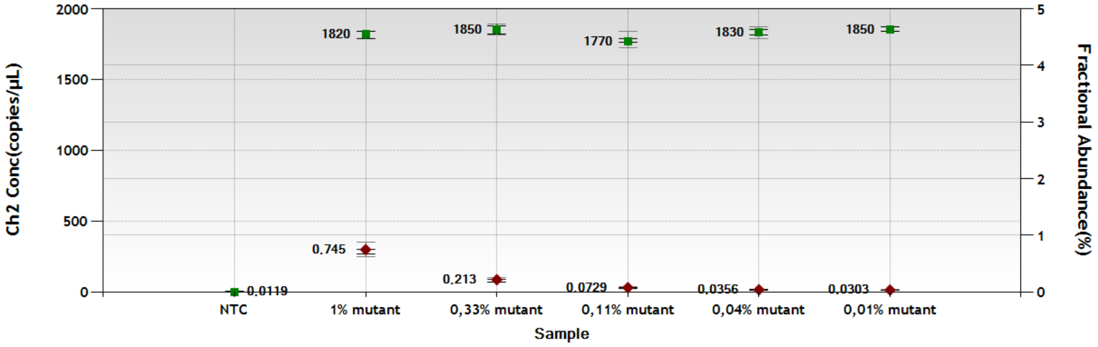

Supplemental Figure S1: Serial dilution of the c.2188C>T mutation control (gBlock), (ADNP p.Arg730*) in a constant control 7 (Supplemental Table S1) background.

Multiplex ddPCR reaction detected both control copies (C) and mutant copies (T). Green squares = number of copies of C in 1 µl of the 20 µl ddPCR reaction. Brown squares, representing % of positive copies in the known background of control copies = fractional abundance. NTC = no template control, blank.

a

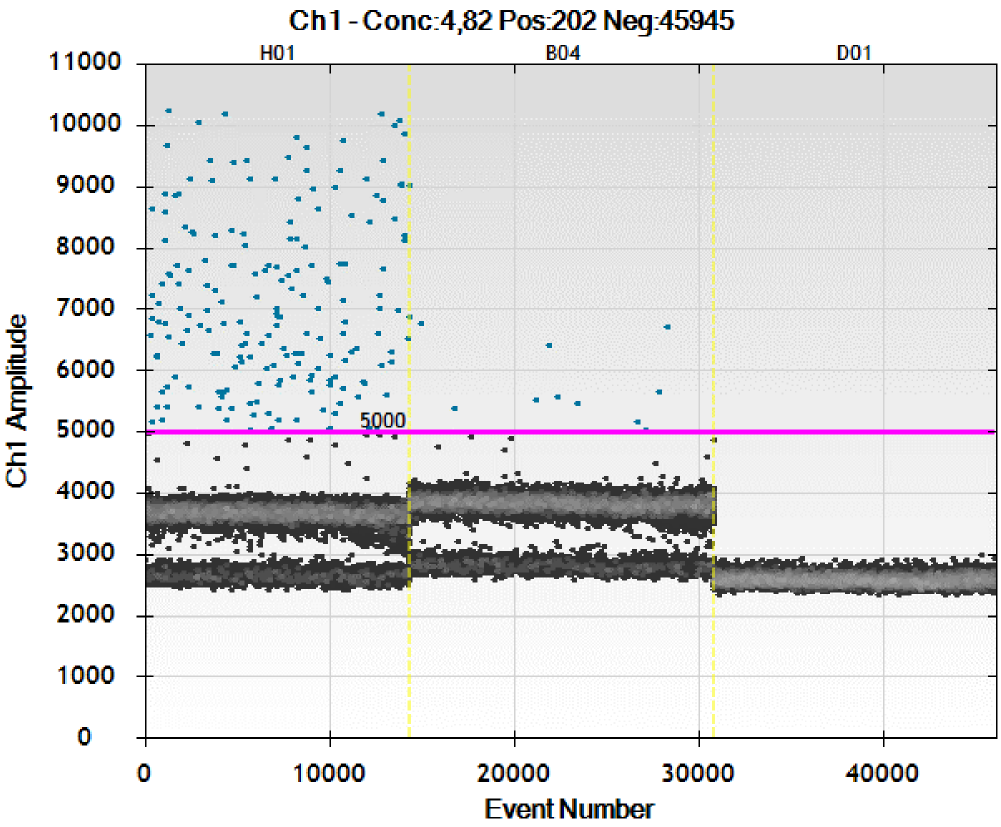

Ch1 - Conc:4,82 Pos:202 Neg:45945
H01
B04
D01
Ch1 Amplitude
Event Number
5000
0
1000
2000
3000
4000
5000
6000
7000
8000
9000
10000
11000
10000
20000
30000
40000

b

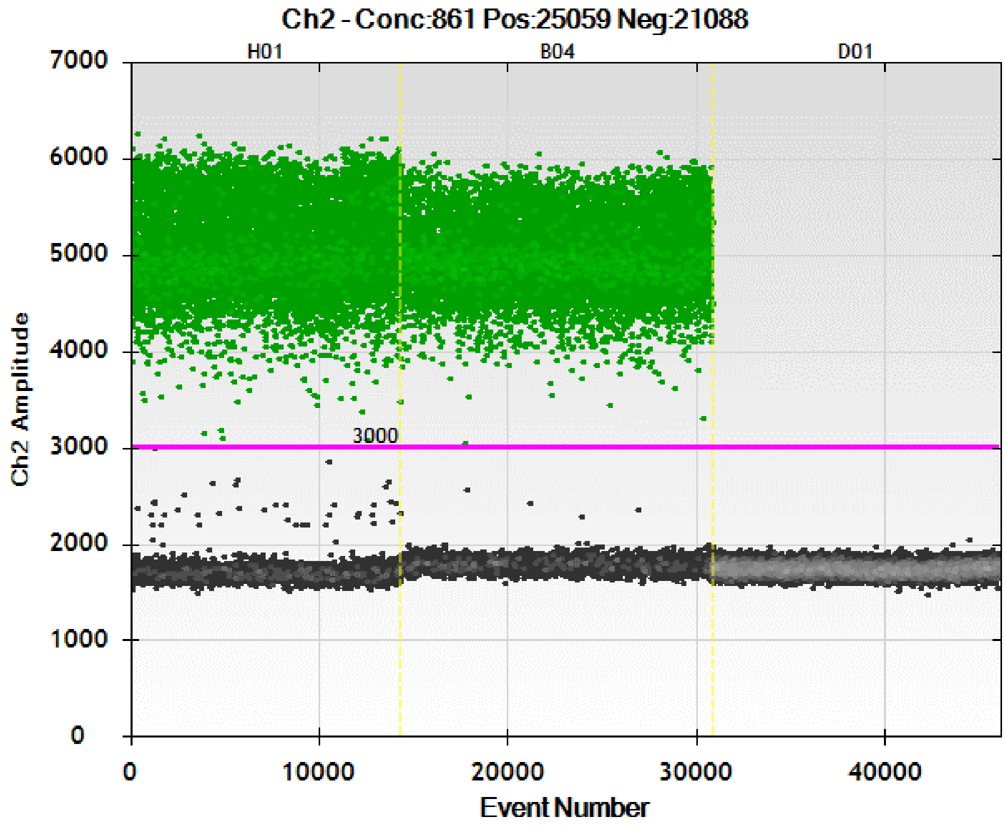
Ch2 - Conc:861 Pos:25059 Neg:21088
H01
B04
D01
7000
6000
5000
4000
3000
2000
1000
0
3000
Ch2 Amplitude
0
10000
20000
30000
40000
Event Number

c

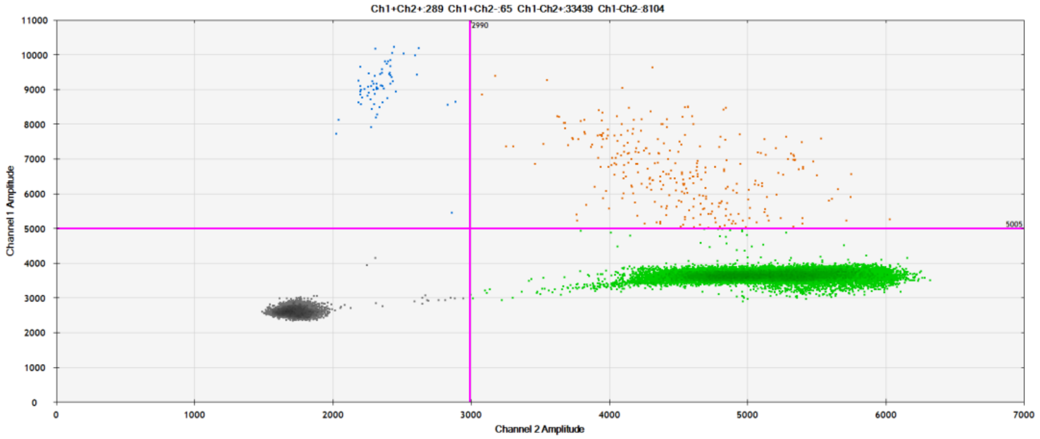
Ch1+Ch2+:289 Ch1+Ch2-:65 Ch1-Ch2+:33439 Ch1-Ch2-:8104
2990
5005
11000
10000
9000
8000
7000
6000
5000
4000
3000
2000
1000
0
Channel 1 Amplitude
0
1000
2000
3000
4000
5000
6000
7000
Channel 2 Amplitude

Supplemental Figure S2 (a-c): 1D amplitude & 2D amplitude dot plots for the ADNP c.2188C>T mutation (positive sample gBlock) in a constant WT background.

a and b: 1D amplitude plots. A mixture containing mutant control DNA gBlock (p.Arg730* c.2188C>T, **1%**) in control subject 7 was subjected to ddPCR. Black droplets are empty droplets without PCR product.

a. The blue histogram indicates the number of droplets considered as positive for the mutant c.2188C>T detected by FAM channel. The first column (H1) represents 0.8% gblock control calculated in the WT background, the second column (A02) shows gblock concentration in 0.04%, the last column (D1) is NTC.
b. The green histogram corresponds to the number of positive WT droplets, detected in channel for HEX for the same samples as at figure A.
c. 2D amplitude plot. A mixture containing mutant control DNA gBlock (p.Arg730* c.2188C>T, **0.8%**) in control subject 7. Black dots are empty droplets without PCR product (Ch1- FAM Ch2- HEX). Green dots represent WT only, single positive droplets (Ch1- FAM Ch2+ HEX). Blue dots represent mutant only, single positive droplets (Ch1+ FAM Ch2- HEX). Orange dots represent doble positive droplets (Ch1+ FAM Ch2+ HEX).

a

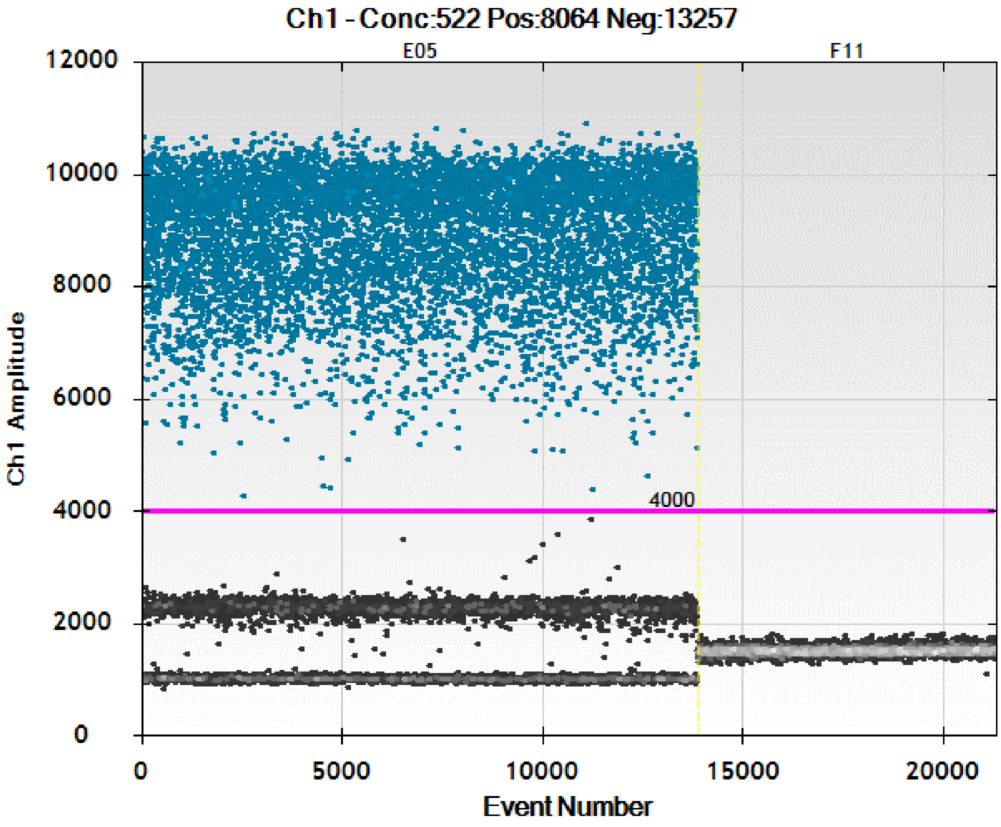
Ch1 - Conc:522 Pos:8064 Neg:13257
E05
F11
12000
10000
8000
6000
4000
2000
0
Ch1 Amplitude
4000
0
5000
10000
15000
20000
Event Number

b

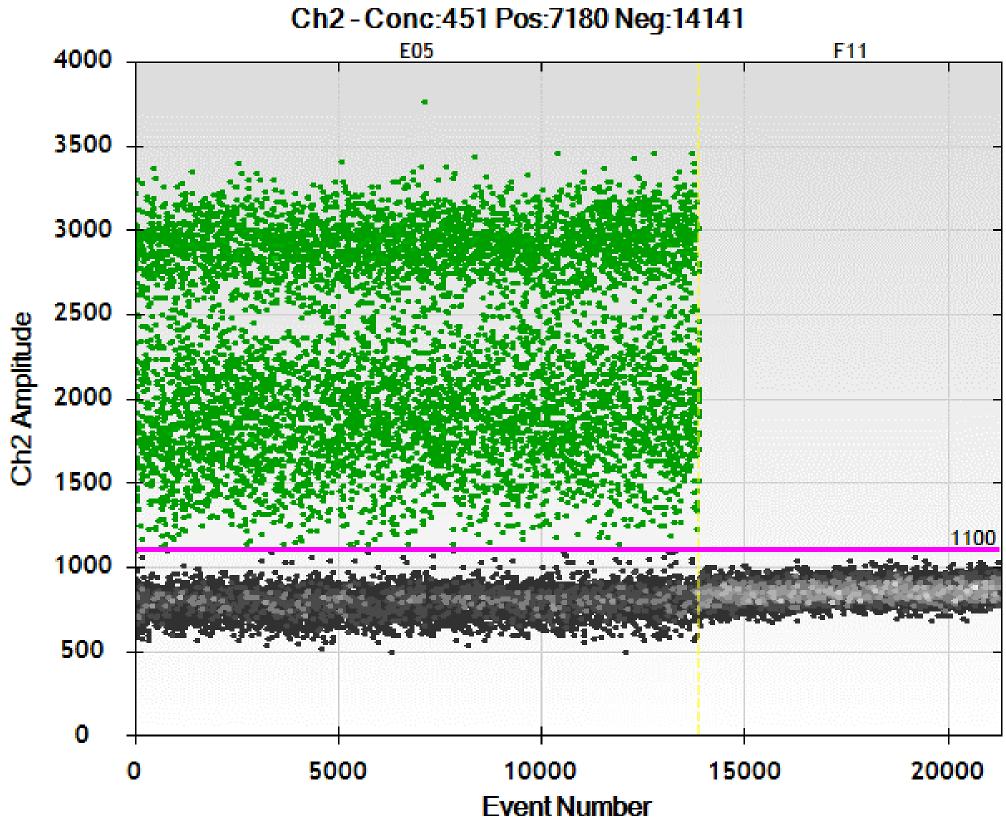
Ch2 - Conc:451 Pos:7180 Neg:14141
E05
F11
Ch2 Amplitude
4000
3500
3000
2500
2000
1500
1000
500
0
1100
0
5000
10000
15000
20000
Event Number

c

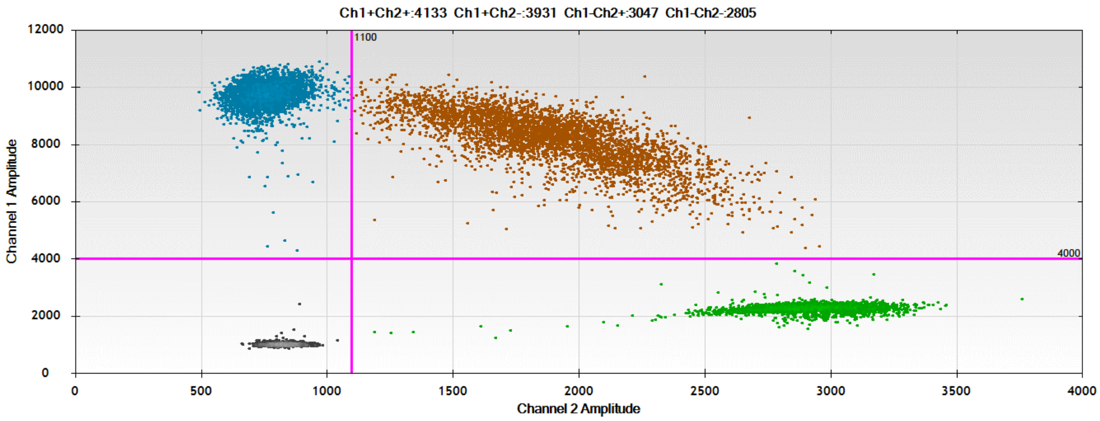
Ch1+Ch2+:4133 Ch1+Ch2-:3931 Ch1-Ch2+:3047 Ch1-Ch2-:2805
Channel 1 Amplitude
12000
10000
8000
6000
4000
2000
0
1100
4000
0
500
1000
1500
2000
2500
3000
3500
4000
Channel 2 Amplitude

Supplemental Figure S3 (a-c): 1D amplitude & 2D amplitude dot plots for the ADNP c.2157C>G mutation (positive sample gBlock) in a constant WT background.

A mixture containing mutant control DNA gBlock (p.Tyr719* c.2157C>G, **50%**) in control subject 7.

a. The blue histogram indicates the number of droplets considered as positive for the mutant c.2157C>G detected by FAM channel. The first column (E05) represents 50% gblock control in the WT background, the second column (F11) is NTC.
b. The green histogram corresponds to the number of positive WT droplets, detected in channel for HEX for the same samples as at figure A.
c. 2D amplitude plot. A mixture containing mutant control DNA gBlock (c.2157C>G mutation, **50%**) in control subject 7. Black dots are empty droplets without PCR product (Ch1- FAM Ch2- HEX). Green dots represent WT only, single positive droplets (Ch1- FAM Ch2+ HEX). Blue dots represent mutant only, single positive droplets (Ch1+ FAM Ch2- HEX). Orange dots represent doble positive droplets (Ch1+ FAM Ch2+ HEX).

Supplemental Table S2 (excel file)

Raw data for the ddPCR studies.

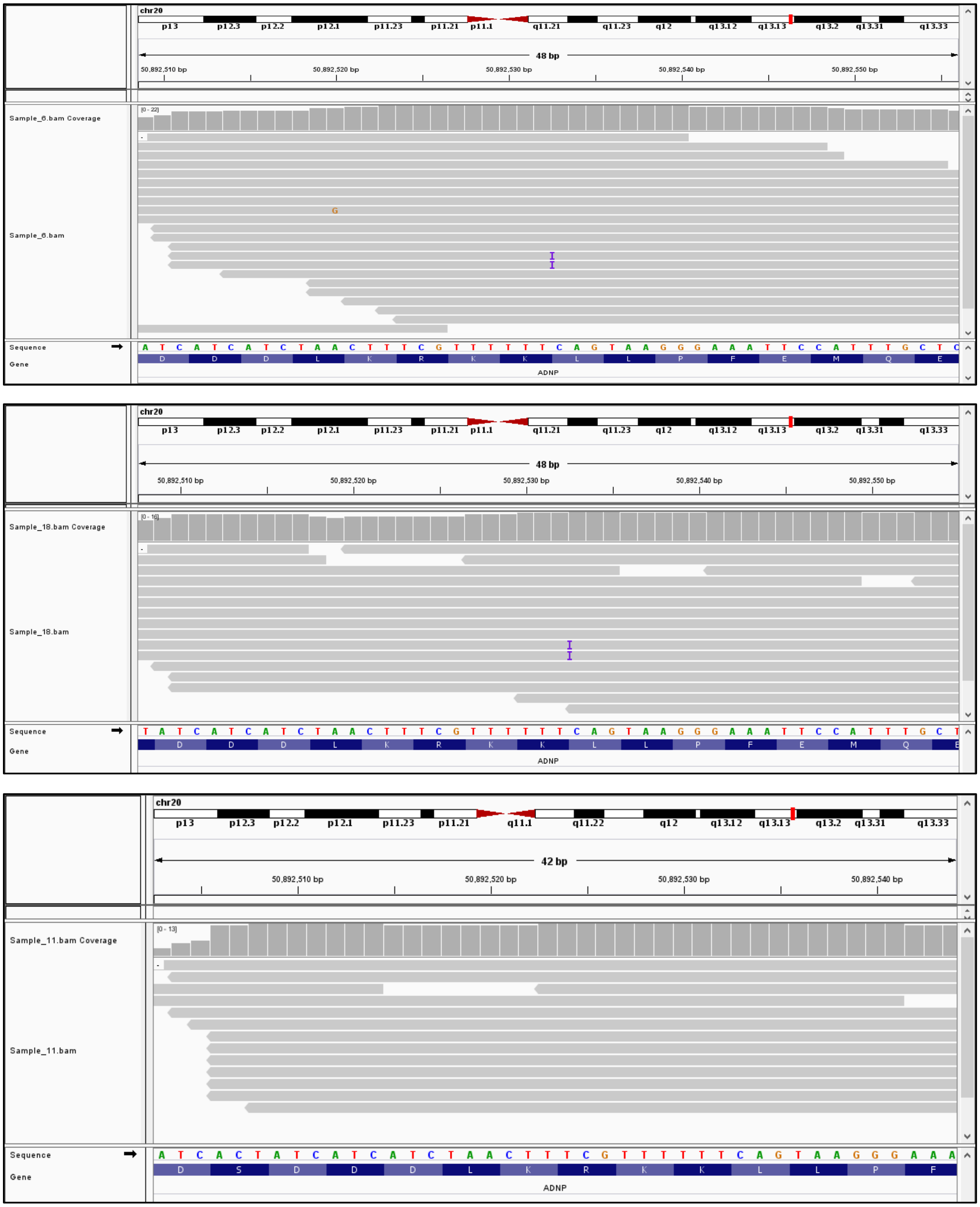

Supplemental Figure S4: <u>RNA-seq results for p.Arg730Thrfs*4 ADNP mutation visualized in Integrative Genomics Viewer (IGV)</u> The grey lines display the reads align to the reference genome. The purple marks are the insertion of T. Sample 6 (Control) and Sample 18 (AD) are positive for the mutation. Sample 11 (AD) is negative for the mutation.

Supplemental Table S3: Raw data for the RNA-seq, olfactory bulb, experimental cohort **(excel file).**

Supplemental Table S4: Hippocampus RNA-seq mutations GSE67333 **(excel file).**

Supplemental Table S5: Dorsolateral prefrontal cortex (DLPFC) RNA-seq mutations GSE53697 **(excel file).**

Supplemental Table S6a: Fusiform gyrus tissue section I RNA-seq mutations GSE95587 and Fusiform gyrus tissue section II GSE125583 **(excel file).**

Supplemental Table S6b: Statistical analysis of mutation frequencies and number per subject in the different brain areas.

(a) Olfactory bulb

ANOVA

**Olfactory bulb**		Sum of Squares	df	Mean Square	F	Sig.
**Mutation frequency per subject**	Between Groups	.017	2	.008	98.064	1.2E-21
	Within Groups	.006	75	.000		
	Total	.023	77			
**Mutation Number per subject**	Between Groups	6908.728	2	3454.364	31.598	1.1E-10
	Within Groups	8199.220	75	109.323		
	Total	15107.949	77			

(b) Hippocampus

Kruskal-Wallis Test

Ranks

Hippocampus		N	Mean Rank
**Mutation frequency per subject**	Control	4	6.75
	AD	4	4.50
	AD+Control	8	11.38
	Total	16	
**Mutation Number per subject**	Control	4	5.50
	AD	4	4.75
	AD+Control	8	11.88
	Total	16	

Test Statistics

	Mutation frequency per subject	Mutation Number per subject
**Chi-Square**	6.281	8.090
**df**	2	2
**Asymp. Sig.**	.043	.018

Mutation frequency per subject

Sample1-Sample2	Test Statistic	Std. Error	Std. Test Statistic	Sig.	Adj.Sig.
AD-Control	2.250	3.367	.668	.504	1.000
AD-AD+Control	-6.875	2.915	-2.358	.018	.055
Control-AD+Control	-4.625	2.915	-1.586	.113	.338

Mutation Number per subject

Sample1-Sample2	Test Statistic	Std. Error	Std. Test Statistic	Sig.	Adj.Sig.
AD-Control	.750	3.367	.223	.824	1.000
AD-AD+Control	-7.125	2.915	-2.444	.015	.044
Control-AD+Control	-6.375	2.915	-2.187	.029	.086

(c) DLPFC
Kruskal-Wallis Test

Ranks

DLPFC		N	Mean Rank
**Mutation frequency per subject**	Control	8	9.63
	AD	9	9.00
	AD+Control	17	25.71
	Total	34	
**Mutation Number per subject**	Control	8	9.00
	AD	9	9.11
	AD+Control	17	25.94
	Total	34	

	Mutation frequency per subject	Mutation Number per subject
**Chi-Square**	23.103	24.441
**df**	2	2
**Asymp. Sig.**	9.6E-06	4.9E-06

Mutation frequency per subject

Sample1-Sample2	Test Statistic	Std. Error	Std. Test Statistic	Sig.	Adj.Sig.
AD-Control	.625	4.839	.129	.897	1.000
AD-AD+Control	-16.706	4.105	-4.070	.000	.000
Control-AD+Control	-16.081	4.270	-3.766	.000	.000

Mutation Number per subject

Sample1-Sample2	Test Statistic	Std. Error	Std. Test Statistic	Sig.	Adj.Sig.
Control-AD	-.111	4.838	-.023	.982	1.000
Control-AD+Control	-16.941	4.269	-3.969	.000	.000
AD-AD+Control	-16.830	4.104	-4.101	.000	.000

(d) Fusiform gyrus tissue section I

ANOVA

**Fusiform gyrus**		Sum of Squares	df	Mean Square	F	Sig.
**Mutation frequency per subject**	Between Groups	.288	2	.1438	3349.429	2.4E-171
	Within Groups	.010	231	.0000		
	Total	.297	233			
**Mutation Number per subject**	Between Groups	46948272	2	23474136	576.657	1.5E-90
	Within Groups	9403382	231	40707		
	Total	56351654	233			

Multiple Comparisons

Bonferroni

**Dependent Variable**			Mean Difference (I-J)	Std. Error	Sig.	95% Confidence Interval	
						Lower Bound	Upper Bound
**Mutation frequency per subject**	Control	AD	.0068719*	.0013461	2E-06	.003626	.010118
		AD +Control	-.0650413*	.0012914	4E-126	-.068156	-.061927
	AD	Control	-.0068719*	.0013461	2E-06	-.010118	-.003626
		AD +Control	-.0719132*	.0009370	7E-166	-.074173	-.069654
	AD +Control	Control	.0650413*	.0012914	4E-126	.061927	.068156
		AD	.0719132*	.0009370	7E-166	.069654	.074173
**Mutation Number per subject**	Control	AD	-9.960	41.451	1	-109.92	90.00
		AD +Control	-902.971*	39.768	3E-60	-998.87	-807.07
	AD	Control	9.960	41.451	1	-90.00	109.92
		AD +Control	-893.011*	28.854	1E-83	-962.59	-823.43
	AD +Control	Control	902.971*	39.768	3E-60	807.07	998.87
		AD	893.011*	28.854	1E-83	823.43	962.59

(e) Fusiform gyrus tissue section II

ANOVA

		Sum of Squares	df	Mean Square	F	Sig.
Mutations frequency per subject	Between Groups	.029	2	.014	838.777	.000
	Within Groups	.010	575	.000		
	Total	.038	577			
Mutations number per subject	Between Groups	102388.340	2	51194.170	187.795	.000
	Within Groups	156749.162	575	272.607		
	Total	259137.502	577			

Multiple Comparisons

Bonferroni

Dependent Variable			Mean Difference	Std. Error	Sig.	95% Confidence Interval	
						Lower Bound	Upper Bound
Mutations frequency per subject	Control	AD	.00400*	.00057	.000	.0026	.0054
		AD+Control	-.01084*	.00055	.000	-.0122	-.0095
	AD	Control	-.00400*	.00057	.000	-.0054	-.0026
		AD+Control	-.01484*	.00037	.000	-.0157	-.0140
Mutations frequency per subject	Control	AD	-1.85819	2.26697	1.000	-7.3012	3.5848
		AD+Control	-28.00326*	2.19947	.000	-33.2842	-22.7223
	AD	Control	1.85819	2.26697	1.000	-3.5848	7.3012
		AD+Control	-26.14508*	1.47921	.000	-29.6967	-22.5935

<u>Statistical analysis for the three groups of mutations (main text Fig. 1e-f):</u> *Control only, AD only and control+AD.* The average mutation and frequencies were calculated for every subject in the control AD groups (SPSS 23). P-values <0.05 were considered significant.

	Mutations Frequency Per subject				Mutations Number Per subject			
	**Control**	**AD**	**P-Value**	**Test**	**Control**	**AD**	**P-Value**	**Test**
**Olfactory bulb**	0.017±0.08	0.025±0.09	1.7E-09	T-test	32.0±13	51.5±19	0.0007	T-test
**Hippocampus**	0.05±0.12	0.07±0.11	2.9E-09	T-test	154.3±79	247.7±29	0.043	Mann-Whitney
**DLPFC**	0.06±0.17	0.08±0.19	2.3E-16	T-test	214.1±33.6	314.6±21	5.3E-04	Mann-Whitney
**Fusiform gyrus tissue section I**	0.030±0.1	0.034±0.1	1.0E-32	T-test	962±285	1032±259	0.208	T-test
**Fusiform gyrus tissue section II**	0.0027±0.03	0.0036±0.03	2.2E-36	T-test	50.35±17.9	71.9±21.3	3.2E-13	T-test

Statistical analysis for AD associated mutations (*AD only* + *control*+*AD*, Fig 1e-f): The average frequencies were calculated for all subjects in the control and the AD groups. The average mutation number were calculated per subject.

Chr20:50903932 c.64_65insA (p.Ile22Asnfs*4)

c.64_65insAA (p.Ile22Lysfs*14)

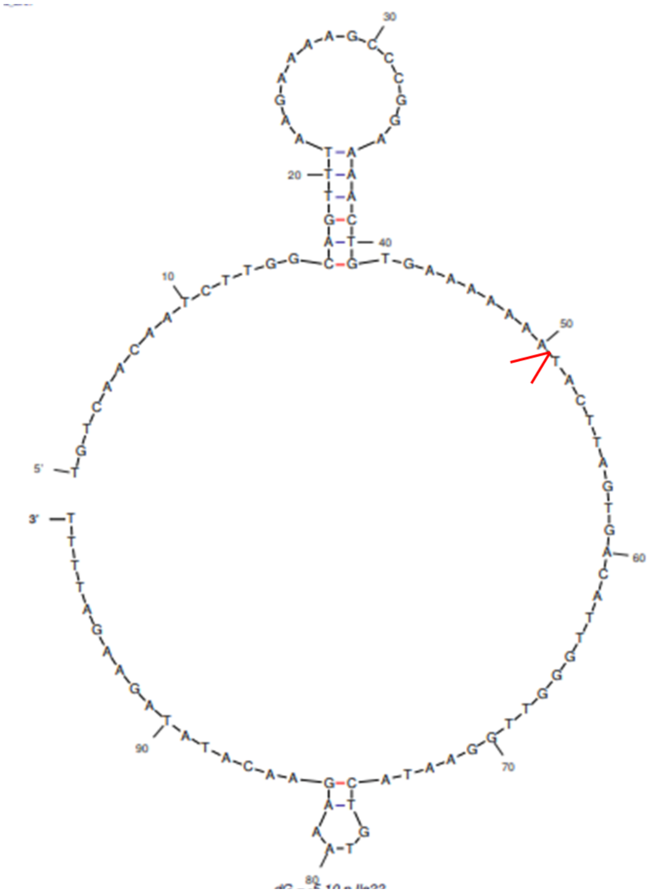

Chr20: 50893379 c.1333_1334delAA (p.Lys445Valfs*7)

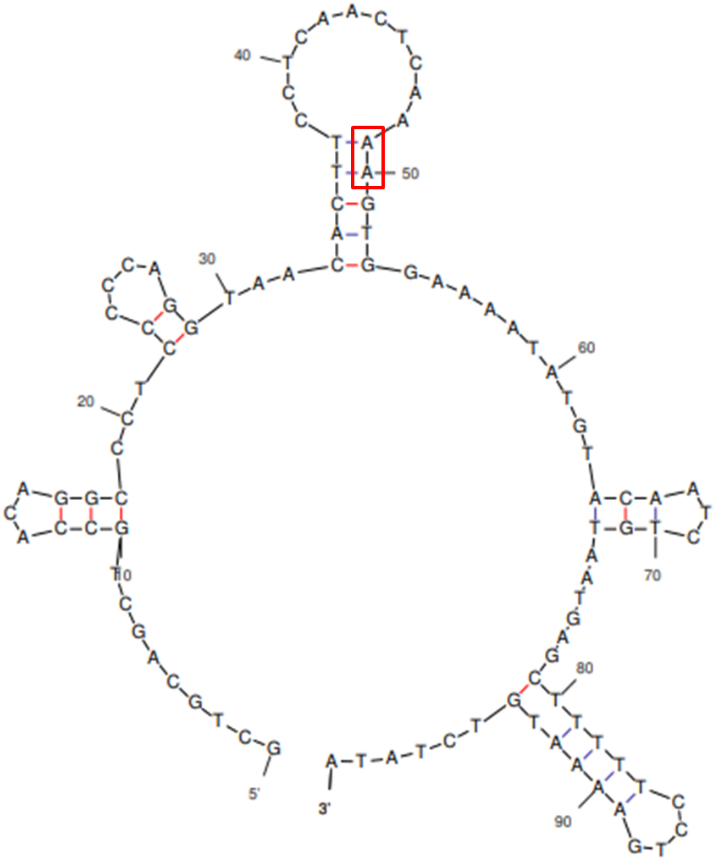

Chr20:50892526 c.2188C>T (p.Arg730*)
c.2187_2188insA (p.Arg730Thrfs*4)

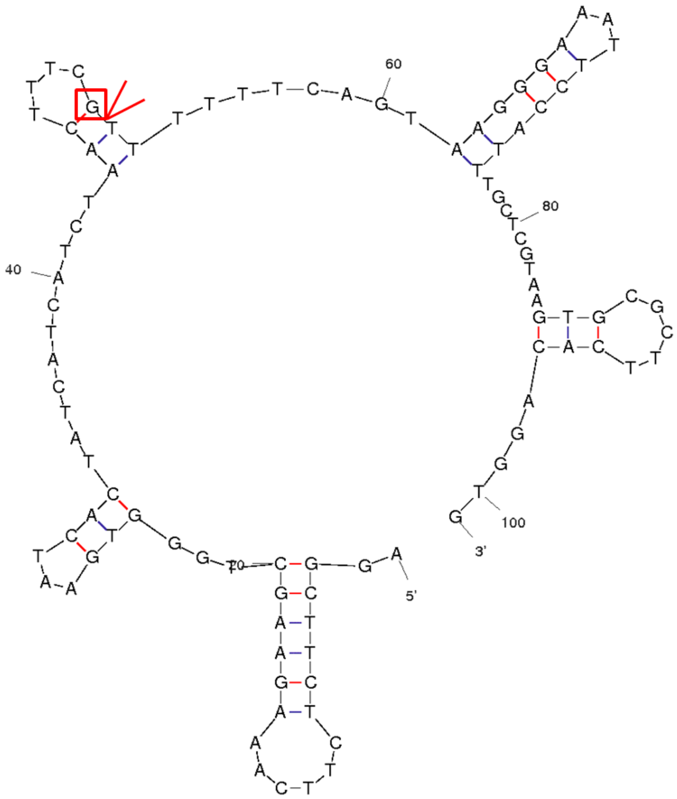

Chr20:50891666 c.3047_3048insA (p.Ala1017Glyfs*5)
c. 3047_3048insAA (p.Arg730Thrfs*4)

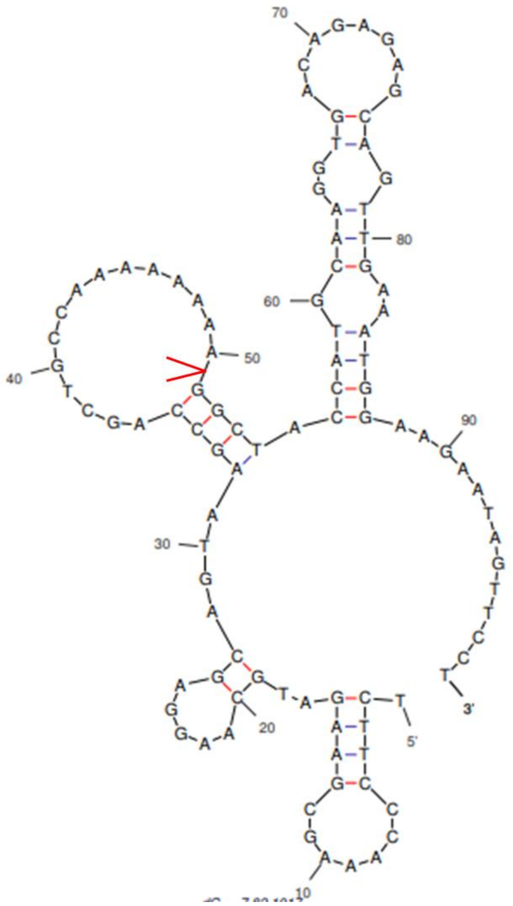

Supplemental Figure S5: <u>Predicted hairpin formation by 100bp genomic sequence surrounding ADNP mutations</u> (a) p.Ile22Asnfs*4, p.Ile22Lysfs*14 (b) p.Lys445Valfs*7 (c) p.Arg730*, p.Arg730Thrfs*4 (d) p.Ala1017Glyfs*5, p.Ala1017Glyfs*10 (performed with Mfold web server http://www.bioinfo.rpi.edu/applications/mfold). Hairpin formation for p.Tyr719* was previously described[1]. Mutated positions are indicated by red structures.

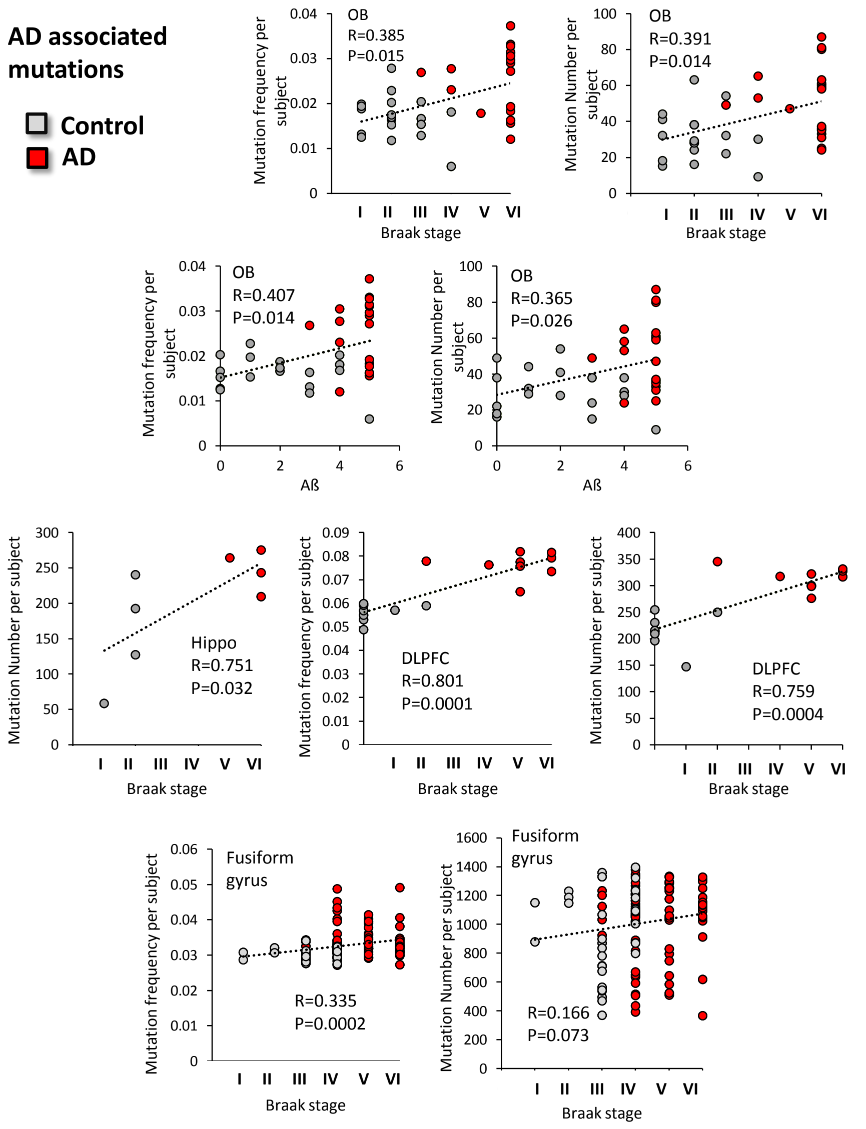

Supplemental Figure S6: <u>Spearman correlations of mutations number per subject and mutations frequency per subject with Braak stage or amyloid beta load in control and AD subjects.</u>

Table S7: Autism (ASD), ID, cytoskeleton, disease-driving genes are mutated in the olfactory bulb of elderly tauopathy controls and AD subjects **(excel file)**.

Supplemental Figure S7: String analysis of interacting mutated proteins **(attached PDF file with hyperlinks).**

Table S8a: Shared disease-driving genes mutations with cytoskeleton genes (GO:020954 and GO:020801) in all tested brain areas, including olfactory bulb, hippocampus, dorsolateral prefrontal cortex (DLPFC) and fusiform gyrus (for details see Fig. 3a) **(excel file)**.

Table S8b: All shared gene mutations with cytoskeleton genes (GO:020954 and GO:020801) in all tested brain areas, including olfactory bulb, hippocampus, dorsolateral prefrontal cortex (DLPFC) and fusiform gyrus **(excel file)**.

Table S9: Shared disease-driving genes mutations with Autism (ASD) and ID genes in all tested brain areas, including olfactory bulb, hippocampus, dorsolateral prefrontal cortex (DLPFC) and fusiform gyrus (for details see Fig. 3b). **(excel file)**.

Table S10: In depth mutation description of shared disease-driving genes with Autism (ASD) and ID genes in all tested brain areas, including olfactory bulb, hippocampus, dorsolateral prefrontal cortex (DLPFC) and fusiform gyrus (for details see Fig. 2b, 3b) **(excel file)**.

Gene name	Protein	Function	Phenotype MIM number	Phenotype
**ADNP**	Activity dependent neuroprotective protein	Homeodomain-containing zinc finger protein with transcription factor activity that is essential for brain formation (Gozes, 2007; Mandel et al., 2007)	615873 ADNP-syndrome	Intellectual disability, autism spectrum disorder, and dysmorphic facial features
**AHI1**	Abelson helper integration site 1	A component of a protein complex in the basal body, a ring-like structure that functions in the transition zone at the base of cilia (Chih et al., 2012)	608629 Joubert syndrome 3	Multiple central nervous system anomalies, including polymicrogyria, malformations of the corpus callosum, seizures, and spasticity
**ANK3**	Ankyrin 3	Located at the nodes of Ranvier and the axon initial segment (responsible for the generation of action potentials in the neuron), and associated with the voltage-dependent sodium channel (Iqbal et al., 2013).	615493 Mental retardation (MRT37)	Moderate intellectual disability, speech delay, severe behavioral abnormalities
**ATRX**	ATR-X	Chromatin-remodeling factor, a putative NTP-binding nuclear protein homologous to several members of the helicase II superfamily (Stayton et al., 1994)	301040 Alpha-thalassemia myelodysplasia syndrome	Mental retardation and alpha-thalassemia without molecular abnormalities of the alpha-globin gene complex on chromosome 16p
**CEP290**	Centrosomal protein, 290kD	Involved in ciliary assembly and ciliary trafficking (Coppieters et al., 2010).	610188 Joubert syndrome 5	psychomotor delay, hypotonia, ataxia, oculomotor apraxia, and neonatal breathing abnormalities
**DYNC1H1**	Dynein, cytoplasmic 1, heavy chain 1	Crucial subunit of the cytoplasmic dynein complex, microtubule-associated motor	614563 Mental retardation13 (MRD13)	Mental retardation associated with variable neuronal migration defects

		protein (Poirier et al., 2013).		resulting in cortical malformations
**FMR1**	Fragile X mental retardation protein	RNA-binding protein, forms a messenger ribonucleoprotein complex that associates with polyribosomes (Jin et al., 2004).	300624 Fragile X syndrome	Moderate to severe mental retardation, macroorchidism, and distinct facial features
**KIF5C**	Kinesin family member 5C	A member of the kinesin superfamily of proteins, microtubule-associated motor proteins (Poirier et al., 2013)	615282 Cortical dysplasia complex, with other brain malformations 2 (CDCBM2)	Severe malformations of cortical development
**OFD1**	OFD	May contribute to the regulation of microtubule dynamics, either by mediating dimerization, or else by binding cytoplasmic dynein heavy chain or microtubules directly (Emes and Ponting, 2001) .	311200 Orofaciodigital syndrome 1 (OFD1)	Malformations of the face, oral cavity, and digits and is transmitted as an X-linked dominant condition with lethality in males
**PHIP**	Pleckstrin homology domain-interacting protein	Plays a role in pancreatic beta cell growth and survival (Farhang-Fallah et al., 2000)	617991 Developmental delay, intellectual disability, obesity, and dysmorphic features	Global developmental delay apparent from infancy, intellectual disability or learning difficulties, behavioral abnormalities, dysmorphic features, and obesity
**SMARCA2**	SWI/SNF-related, matrix-associated, actin-dependent regulator of chromatin, subfamily A, member 2	A part of SWI/SNF remodeling complex	601358 Nicolaides-baraitser syndrome	Severe mental retardation, early-onset seizures, short stature, dysmorphic facial features, and sparse hair

SYNE1	Spectrin repeat-containing nuclear envelope protein 1	A member of the spectrin family of structural proteins that link the plasma membrane to the actin cytoskeleton (Schuurs-Hoeijmakers et al., 2013).	610743 spinocerebellar ataxia, autosomal recessive 8	Late-onset cerebellar ataxia with slow progression accompanied by dysarthria

Supplemental Table S11: OMIM in depth description: Autism (ASD), ID, cytoskeleton, disease-driving genes are mutated in the olfactory bulb of elderly tauopathy controls and AD subjects (for details see Supplemental Table S3 and Fig. 2b), as well as genes shared with all tested brain areas, including hippocampus, dorsolateral prefrontal cortex (DLPFC) and fusiform gyrus (for details see Fig. 3a,b). In red, mutated genes specific to AD in the olfactory bulb.

Table short description: Regarding the olfactory bulb (Fig. 2c), some interesting proteins include ADNP and dynein cytoplasmic 1 heavy chain 1 (DYNC1H1), a crucial component of the MT motor system[14]. AD unique mutated proteins (Fig. 2c, red) were divided into two classes, DNA/chromatin remodeling including ATR-X, Fragile X mental retardation protein (FMR1), and SWI/SNF-related, matrix-associated, actin-dependent regulator of chromatin, subfamily A, member 2 (SMARCA2), and cytoplasmic cytoskeleton-linked proteins including ankirin3 (ANK3) and spectrin repeat-containing nuclear envelope protein 1 (SYNE1).

Regarding shared mutated genes (Fig. 3a,b), AD-related mutations of interest in ANK2, identified as a major gene impacting autism[15, 16] and aging[17] and human leukocyte antigen (HLA) forms, overlapped in all brain areas and autism spectrum disorders (ASD). Genome-wide association studies have identified HLA forms as risk genes for AD[18], participating in AD structural brain alteration and modulating AD susceptibility[19], and involved in ASD gut-brain axis dysregulation[20]. Pericentrin (PCNT), centrosome χtubulin interacting mutations, associated with microcephaly overlapped with ID in all brain regions. Lastly, all brain area shared two mutated genes calpain 2 (CAPN2) and protein-tyrosine phosphatase, nonreceptor type, substrate 1 (SIRPA) (not shared with either ASD or ID). However, CAPN2 was associated with chronic traumatic encephalopathy[21], and SIPRA was linked to authophagy[22].

Supplemental Table S12a: RNA-seq from different cell types in the superior frontal gyrus (see Fig. 3c-e, legend)[23] **(Excel file).**

Supplemental Table S12b: Statistical analysis of different mutation frequencies and number per subject in different cell types.

Astrocyte

ANOVA

Source of Variation	*SS*	*df*	*MS*	*F*	*P-value*	*F crit*
Between Groups	3.641627	2	1.820813	169.8802	<0.00001	2.995941
Within Groups	461.6659	43073	0.010718			
Total	465.3075	43075				

	Astrocyte Control	*Astrocyte AD*
Mean	0.024255	0.04589
Variance	0.008334	0.016987
Observations	26902	10103
Hypothesized Mean Difference	0	
df	13989	
t Stat	-15.3326	
P(T<=t) one-tail	6.2E-53	
t Critical one-tail	1.644963	
P(T<=t) two-tail	1.24E-52	
t Critical two-tail	1.960134	

	Astrocyte Control	*Astrocyte AD+C*
Mean	0.024255	0.036409
Variance	0.008334	0.010851
Observations	26902	6071
Hypothesized Mean Difference	0	
df	8300	
t Stat	-8.39301	
P(T<=t) one-tail	2.76E-17	
t Critical one-tail	1.645037	
P(T<=t) two-tail	5.52E-17	
t Critical two-tail	1.96025	

	Astrocyte AD	*Astrocyte AD+C*
Mean	0.04589	0.036409
Variance	0.016987	0.010851
Observations	10103	6071
Hypothesized Mean Difference	0	
df	14925	
t Stat	5.090681	
P(T<=t) one-tail	1.81E-07	
t Critical one-tail	1.644956	
P(T<=t) two-tail	3.61E-07	
t Critical two-tail	1.960123	

Neuron

ANOVA

Source of Variation	*SS*	*df*	*MS*	*F*	*P-value*	*F crit*
Between Groups	0.268096	2	0.134048	9.824918	5.42E-05	2.995948
Within Groups	566.7448	41539	0.013644			
Total	567.0129	41541				

	Neuron C	*Neuron AD*
Mean	0.026458	0.034411
Variance	0.013081	0.013729
Observations	17867	3326
Pooled Variance	0.013183	
Hypothesized Mean Difference	0	
df	21191	
t Stat	-3.66767	
P(T<=t) one-tail	0.000123	
t Critical one-tail	1.644926	
P(T<=t) two-tail	0.000245	
t Critical two-tail	1.960076	

	Neuron C	Neuron AD+Control
Mean	0.026458	0.033574
Variance	0.013081	0.015057
Observations	17867	6231
Pooled Variance	0.013592	
Hypothesized Mean Difference	0	
df	24096	
t Stat	-4.1486	
P(T<=t) one-tail	1.68E-05	
t Critical one-tail	1.644917	
P(T<=t) two-tail	3.36E-05	
t Critical two-tail	1.960062	

Endothelial

ANOVA

Source of Variation	SS	df	MS	F	P-value	F crit
Between Groups	5.419804	2	2.709902	121.2178	<0.00001	2.995856
Within Groups	1624.853	72682	0.022356			
Total	1630.272	72684				

	Control	AD
Mean	0.038298	0.058326
Variance	0.019788	0.029021
Observations	48665	18684
Hypothesized Mean Difference	0	
df	28983	
t Stat	-14.3065	
P(T<=t) one-tail	1.43E-46	
t Critical one-tail	1.644906	
P(T<=t) two-tail	2.86E-46	
t Critical two-tail	1.960046	

	Control	AD+Control
Mean	0.038299	0.044782
Variance	0.019789	0.022437
Observations	48664	5335
Pooled Variance	0.02005	
Hypothesized Mean Difference	0	
df	53997	
t Stat	-3.17458	
P(T<=t) one-tail	0.000751	
t Critical one-tail	1.644882	
P(T<=t) two-tail	0.001501	
t Critical two-tail	1.960008	

	Endothelial-AD	*Endothelial-AD+C*
Mean	0.058326	0.044773
Variance	0.029021	0.022433
Observations	18684	5336
Pooled Variance	0.027557	
Hypothesized Mean Difference	0	
df	24018	
t Stat	5.259908	
P(T<=t) one-tail	7.27E-08	
t Critical one-tail	1.644917	
P(T<=t) two-tail	1.45E-07	
t Critical two-tail	1.960063	

Myeloid

ANOVA

Source of Variation	*SS*	*df*	*MS*	*F*	*P-value*	*F crit*
Between Groups	1.873171	2	0.936586	61.37608	<0.00001	2.995924
Within Groups	712.5097	46692	0.01526			
Total	714.3829	46694				

	Myeloid-C	*Myeloid-AD*
Mean	0.030238	0.044441
Variance	0.013792	0.018438
Observations	29196	13379
Hypothesized Mean Difference	0	
df	22889	
t Stat	-10.4403	
P(T<=t) one-tail	9.26E-26	
t Critical one-tail	1.64492	
P(T<=t) two-tail	1.85E-25	
t Critical two-tail	1.960068	

	Myeloid-C	*Myeloid-AD+Control*
Mean	0.030238	0.037155
Variance	0.013792	0.015345
Observations	29196	4120
Hypothesized Mean Difference	0	
df	5218	
t Stat	-3.37633	
P(T<=t) one-tail	0.00037	
t Critical one-tail	1.645146	
P(T<=t) two-tail	0.00074	
t Critical two-tail	1.960419	

	Myeloid-AD	*Myeloid-AD+Control*
Mean	0.044441	0.037155
Variance	0.018438	0.015345
Observations	13379	4120
Hypothesized Mean Difference	0	
df	7418	
t Stat	3.225082	
P(T<=t) one-tail	0.000632	
t Critical one-tail	1.645059	
P(T<=t) two-tail	0.001265	
t Critical two-tail	1.960284	

Statistical analysis Superior frontal gyrus different cell types for - the three groups of mutations (Fig 3c): Control only, AD only and control+AD. Mutation frequencies

were calculated for every subject in the control AD groups (SPSS 23). P-values <0.05 were considered significant.

	Mutations Frequency Per subject				Mutations Number Per subject			
	**Control**	**AD**	**P-Value**	**Test**	**Control**	**AD**	**P-Value**	**Test**
Endothelial	0.007±0.006	0.08±0.05	0.002	T-test	28±20	346±289	0.0064	T-test
Astrocyte	0.006±0.002	0.046±0.02	0.003	T-test	66.4±39.4	314.5±119	0.001	T-test
Myeloid	0.004±0.0014	0.047±0.02	0.0001	T-test	25.5±10.2	223.8±139.8	0.002	T-test
Neuron	0.005±0.003	0.03±0.02	0.00003	T-test	21.3±15.9	99.9±162	0.038	T-test

Statistical analysis for AD associated mutations (*AD only* + *control*+*AD*, Fig 3c): The average frequencies were calculated for all subjects in the control and the AD groups. The average mutation number were calculated per subject.

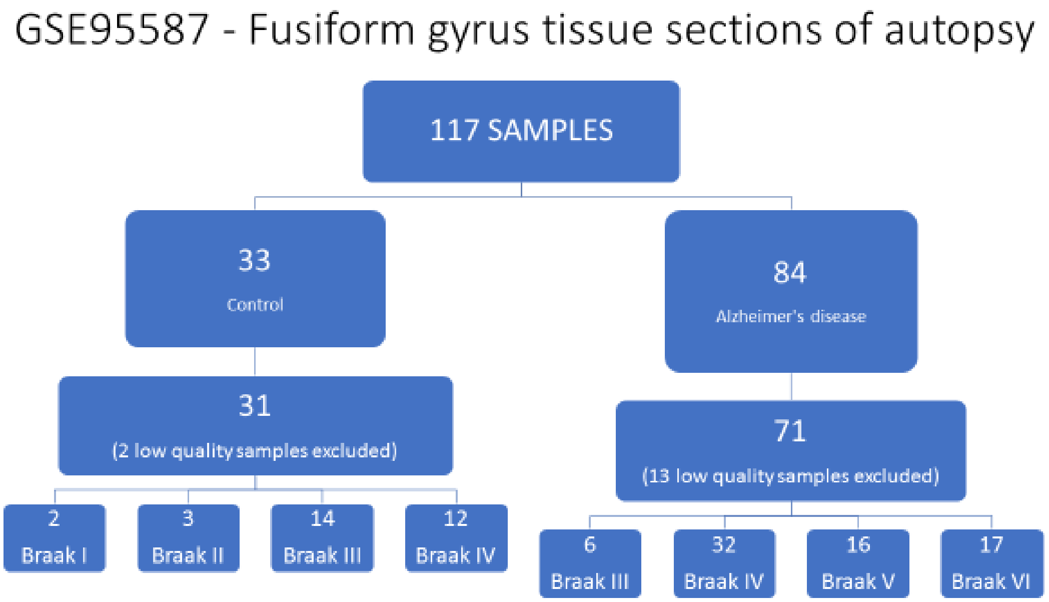

Supplemental Figure S8: Data mining of RNA-Seq results from postmortem fusiform gyrus tissue sections. Numbers of individual subjects are depicted.

Supplemental Table S13: <u>Specific cell type enriched ADNP mutations</u>

**Genome**	**cDNA**	**Protein**	**Cell type**
chr20:50903939	c.58A<T	p.Lys20*	Endothelial
chr20:50903936	c.61A<T	p.Lys21*	Endothelial
chr20:50894466	c.248C<A	p.Ser83*	Myleloid
chr20:50894336	c.376_377insT	p.Lys126Ilefs*12	Astrocyte
chr20:50894329	c.380_384delTATTT	p.Ile127Thrfs*9	Astrocyte
chr20:50894195	c.517_518delCG	p.Arg173synfs*4	Myleloid, endothel
chr20:50894193	c.520_521insTA	p.Asp174Valfs*6	Myleloid, endothel
chr20:50893958	c.754_755delAC	p.Thr252Cysfs*40	Neuron
chr20:50893888	c.826C<T	p.Gln276*	Endothelial
chr20:50893729	c.985C<T	p.Gln329*	Myleloid
chr20:50893702	c.1011_1012insACCCT	p.Gly338Thrfs*11	Astrocyte
chr20:50893639	c.1074_1075	p.Ile359Hisfs*39	Astrocyte
chr20:50893638	c.1075_1076insCC	p.Ile359Thrfs*8	Endothelial
chr20:50893635	c.1077_1078delTC	p.Pro360Serfs*10	Endothelial
chr20:50893597	c.1117G<T	p.Gly373*	Myleloid
chr20:50893384	c.1330C<T	p.Gln444*	Endothelial
chr20:50893267	c.1447A<T	p.Lys483*	Endothelial
chr20:50893233	c.1479_1480delCT	p.Tyr493*	Endothelial
chr20:50893229	c.1484_1485insAA	p.Asn495Lysfs*14	Endothelial
chr20:50891848	c.2866G<T	p.Glu956*	Astrocyte

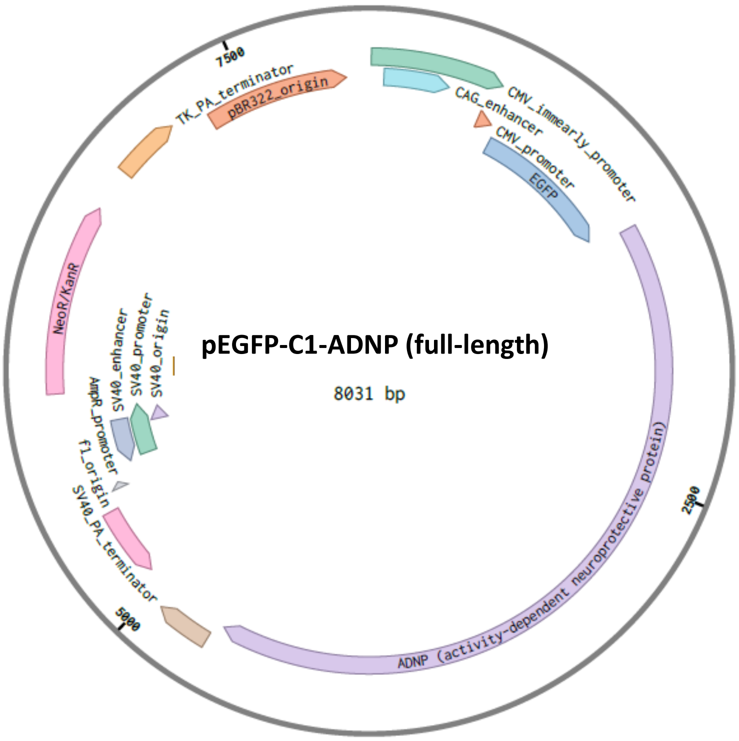
pEGFP-C1-ADNP (full-length)
8031 bp
7500
2500
5000
CMV_immearly_promoter
CAG_enhancer
CMV_promoter
EGFP
ADNP (activity-dependent neuroprotective protein)
SV40_PA_terminator
f1_origin
AmpR_promoter
SV40_enhancer
SV40_promoter
SV40_origin
NeoR/KanR
TK_PA_terminator
pBR322_origin

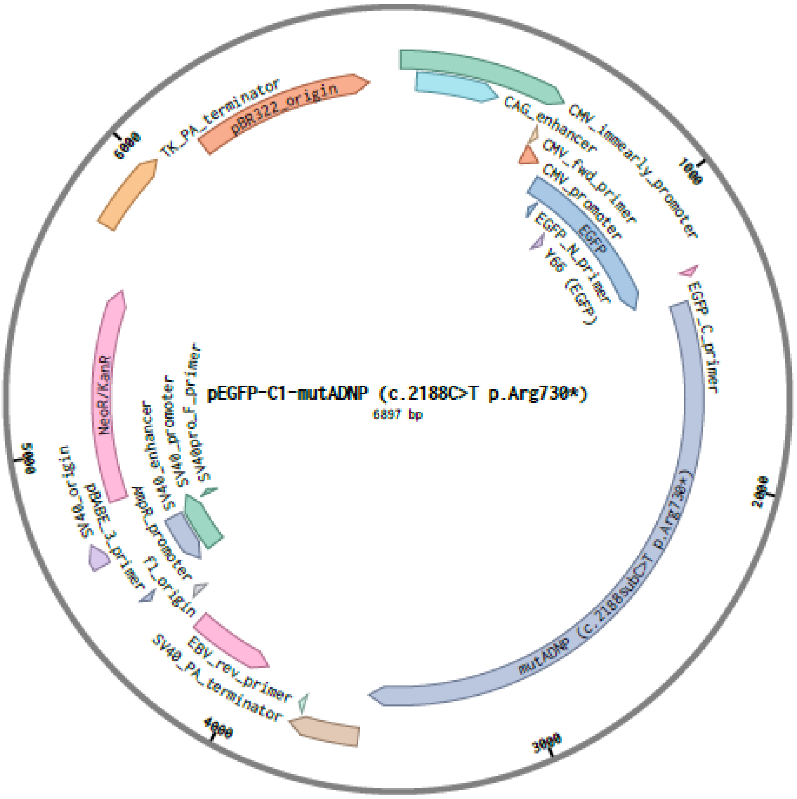
pEGFP-C1-mutADNP (c.2188C>T p.Arg730*)
6897 bp
1000
2000
3000
4000
5000
6000
CMV_immearly_promoter
CAG_enhancer
CMV_fwd_primer
CMV_promoter
EGFP
EGFP_N_primer
Y66 (EGFP)
EGFP_C_primer
mutADNP (c.2188subC>T p.Arg730*)
EBV_rev_primer
SV40_PA_terminator
f1_origin
AmpR_promoter
SV40_enhancer
SV40_promoter
SV40pro_F_primer
SV40_origin
pBABE_3_primer
NeoR/KanR
TK_PA_terminator
pBR322_origin

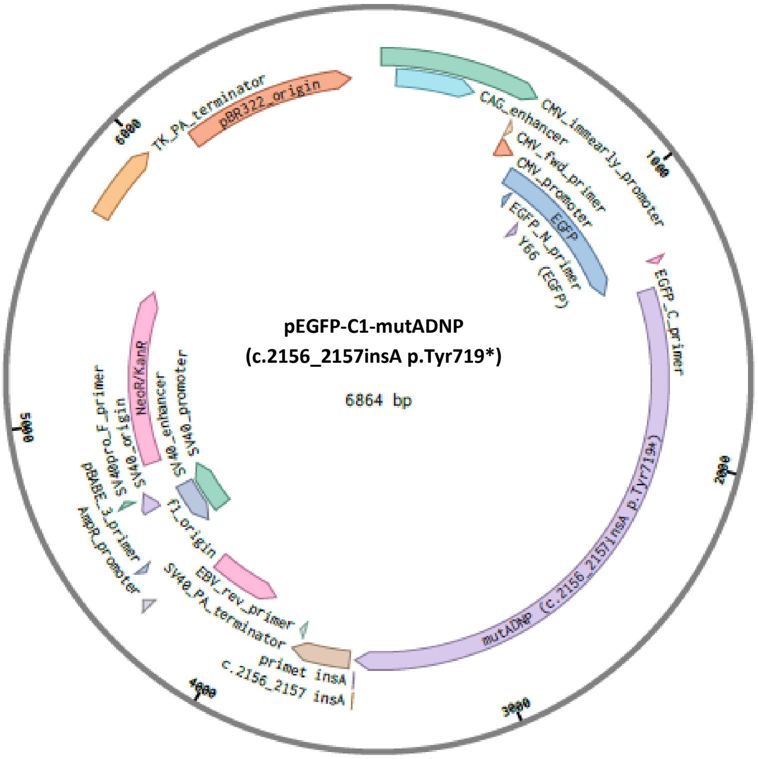
pEGFP-C1-mutADNP
(c.2156_2157insA p.Tyr719*)
6864 bp
1000
2000
3000
4000
5000
6000
CMV_immearly_promoter
CAG_enhancer
CMV_fwd_primer
CMV_promoter
EGFP
EGFP_N_primer
Y66 (EGFP)
EGFP_C_primer
mutADNP (c.2156_2157insA p.Tyr719*)
primet insA
c.2156_2157 insA
EBV_rev_primer
SV40_PA_terminator
f1_origin
AmpR_promoter
pBABE_3_primer
SV40pro_F_primer
SV40_origin
SV40_enhancer
SV40_promoter
NeoR/KanR
TK_PA_terminator
pBR322_origin

Supplemental Figure S9: Plasmid maps. Protein expressing plasmids were based on pEGFP-C1 vector and express full-length ADNP or the following truncated ADNP proteins: mutADNP-p.Arg730*, mutADNP-p.Tyr719*. The plasmid maps were constructed with Benchling platform (www.benchling.com).

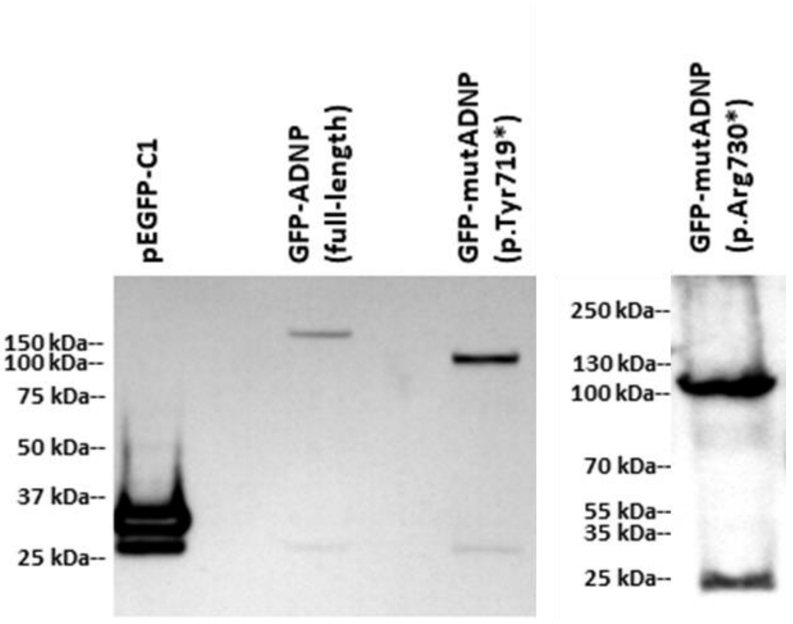

Supplemental Figure S10: Verification of ADNP truncated protein expression by Western blotting. HEK293T cells were transfected with constructed plasmids (described in Fig. S9) and whole-cell lysate proteins were extracted 48hrs after transfection. Blots were exposed to GFP-antibody as before[24, 25]. Expected molecular weight of GFP-conjugated proteins:

GFP-full-length ADNP – 151.5kDa; GFP-mutADNP-p.Arg730* - 109.2kDa; GFP-mutADNP-p.Tyr719* - 107.8kDa, GFP 26.9kDa. Note, as obvious, the p.Arg730* protein was separated on an independent gel.

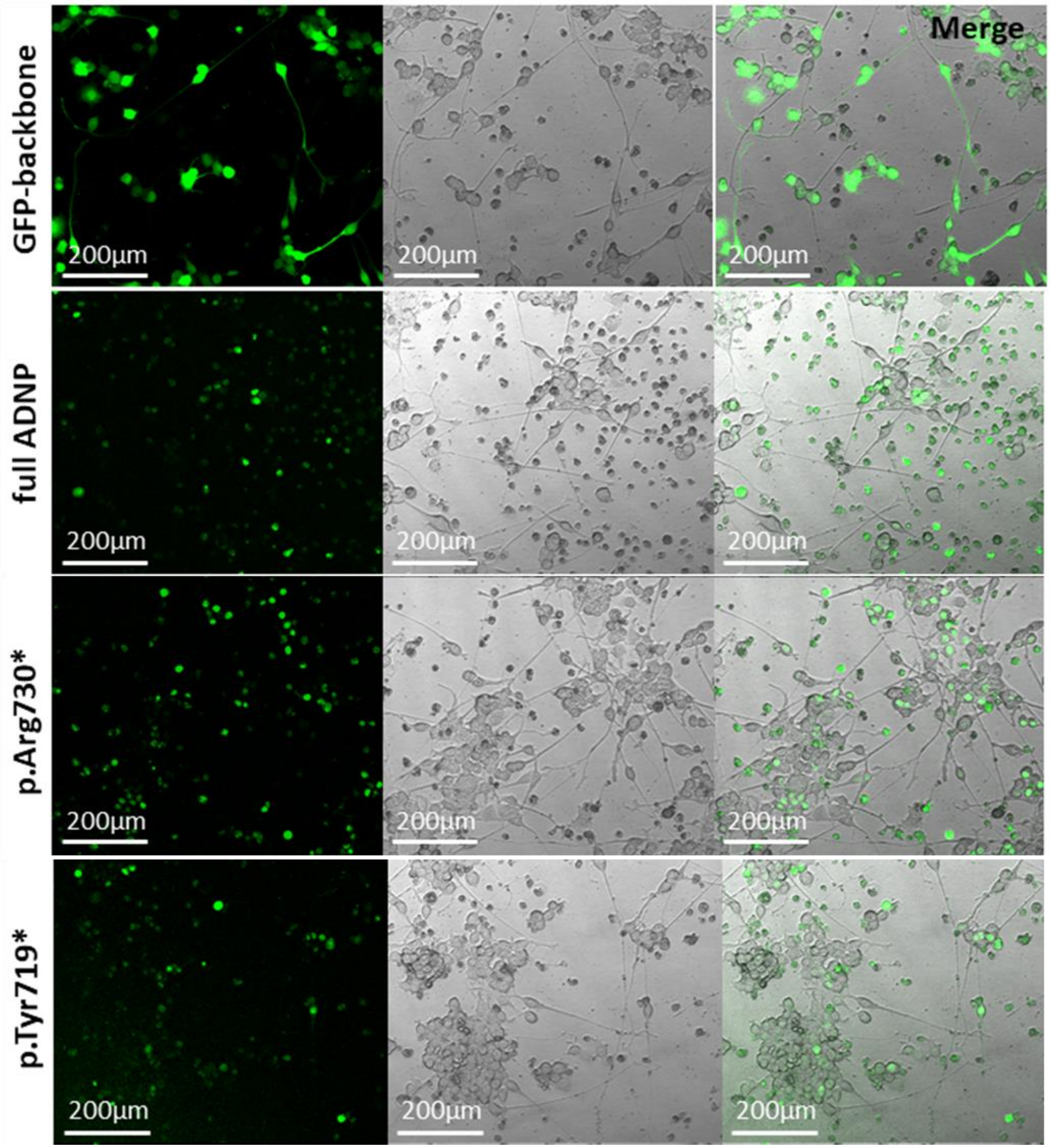

Supplemental Figure S11: <u>Verification of plasmid expression by fluorescent imaging.</u> Differentiated N1E-115 cells were transfected with constructed plasmids (described in Fig. S9). Pictures were taken by fluorescence microscopy 48hrs after transfection.

Table S14: Statistical analysis of cell culture results (main text Figures 4 and 5).

Statistical data for Fig. 4C (Track Length):

Source of Variation	DF	SS	MS	F	P
Plasmid	3	5.085	1.695	12.937	<0.001
treatment	1	0.63	0.63	4.809	0.03
plasmid x treatment	3	0.879	0.293	2.237	0.087
Residual	130	17.032	0.131		
Total	137	25.007	0.183		

All Pairwise Multiple Comparison Procedures (Fisher LSD Method):

Comparisons for factor: plasmid				
Comparison	Diff of Means	LSD (alpha=0.050)	P	Diff >= LSD
full ADNP vs. p.Arg730*	0.527	0.168	<0.001	Yes
full ADNP vs. Control	0.35	0.2	<0.001	Yes
full ADNP vs. p.Tyr719*	0.292	0.187	0.002	Yes
p.Tyr719* vs. p.Arg730*	0.235	0.163	0.005	Yes
p.Tyr719* vs. Control	0.0583	0.195	0.555	No
Control vs. p.Arg730*	0.177	0.178	0.051	No
Comparisons for factor: treatment				
NAP treatment vs. w/o treatment	0.143	0.129	0.03	Yes
Comparisons for factor: treatment within Control				
w/o treatment vs. NAP treatment	0.0822	0.293	0.58	No
Comparisons for factor: treatment within full ADNP				
NAP treatment vs. w/o treatment	0.035	0.271	0.798	No
Comparisons for factor: treatment within p.Arg730*				
NAP treatment vs. w/o treatment	0.274	0.201	0.008	Yes

Comparisons for factor: treatment within p.Tyr719*				
NAP treatment vs. w/o treatment	0.344	0.257	0.009	Yes
Comparisons for factor: plasmid within w/o treatment				
full ADNP vs. p.Arg730*	0.647	0.226	<0.001	Yes
full ADNP vs. p.Tyr719*	0.447	0.262	<0.001	Yes
full ADNP vs. Control	0.292	0.276	0.038	Yes
Control vs. p.Arg730*	0.355	0.233	0.003	Yes
Control vs. p.Tyr719*	0.155	0.267	0.254	No
p.Tyr719* vs. p.Arg730*	0.2	0.216	0.069	No
Comparisons for factor: plasmid within NAP treatment				
full ADNP vs. Control	0.409	0.289	0.006	Yes
full ADNP vs. p.Arg730*	0.408	0.25	0.002	Yes
full ADNP vs. p.Tyr719*	0.137	0.266	0.309	No
p.Tyr719* vs. Control	0.272	0.284	0.061	No
p.Tyr719* vs. p.Arg730*	0.27	0.245	0.031	Do Not Test
p.Arg730* vs. Control	0.00146	0.269	0.991	Do Not Test

Statistical data for Fig. 4C (Comet Speed):

Source of Variation	DF	SS	MS	F	P
Plasmid	3	0.00553	0.00184	4.043	0.009
treatment	1	0.00302	0.00302	6.613	0.011
plasmid x treatment	3	0.0052	0.00173	3.801	0.012
Residual	130	0.0593	0.000456		
Total	137	0.0755	0.000551		

All Pairwise Multiple Comparison Procedures (Fisher LSD Method):

Comparisons for factor: plasmid				
Comparison	Diff of Means	LSD(alpha=0.050)	P	Diff >= LSD
full ADNP vs. p.Tyr719*	0.017	0.011	0.003	Yes
full ADNP vs. p.Arg730*	0.0157	0.00994	0.002	Yes
full ADNP vs. Control	0.0135	0.0118	0.025	Yes
Control vs. p.Tyr719*	0.0035	0.0115	0.548	No
Control vs. p.Arg730*	0.00223	0.0105	0.675	Do Not Test
p.Arg730* vs. p.Tyr719*	0.00127	0.00963	0.794	Do Not Test
Comparisons for factor: treatment				
NAP treatment vs. w/o treatment	0.00988	0.0076	0.011	Yes
Comparisons for factor: treatment within Control				
w/o treatment vs. NAP treatment	0.0128	0.0173	0.145	No
Comparisons for factor: treatment within full ADNP				
NAP treatment vs. w/o treatment	0.0128	0.016	0.116	No
Comparisons for factor: treatment within p.Arg730*				
NAP treatment vs. w/o treatment	0.0137	0.0118	0.023	Yes
Comparisons for factor: treatment within p.Tyr719*				
NAP treatment vs. w/o treatment	0.0259	0.0152	<0.001	Yes
Comparisons for factor: plasmid within w/o treatment				
full ADNP vs. p.Tyr719*	0.0235	0.0155	0.003	Yes
full ADNP vs. p.Arg730*	0.0162	0.0134	0.018	Yes

full ADNP vs. Control	0.00067	0.0163	0.935	No
Control vs. p.Tyr719*	0.0229	0.0158	0.005	Yes
Control vs. p.Arg730*	0.0155	0.0137	0.027	Yes
p.Arg730* vs. p.Tyr719*	0.00734	0.0127	0.257	No
Comparisons for factor: plasmid within NAP treatment				
full ADNP vs. Control	0.0263	0.017	0.003	Yes
full ADNP vs. p.Arg730*	0.0152	0.0147	0.043	Yes
full ADNP vs. p.Tyr719*	0.0104	0.0157	0.192	No
p.Tyr719* vs. Control	0.0158	0.0168	0.064	No
p.Tyr719* vs. p.Arg730*	0.00479	0.0144	0.512	Do Not Test
p.Arg730* vs. Control	0.0111	0.0159	0.17	Do Not Test

Statistical data for Fig. 5C:

Source of Variation	DF	SS	MS	F	P
Plasmid	3	0.0848	0.0283	2.127	0.097
treatment	1	0.0609	0.0609	4.585	0.033
plasmid x treatment	3	0.171	0.057	4.294	0.006
Residual	261	3.467	0.0133		
Total	268	3.945	0.0147		

All Pairwise Multiple Comparison Procedures (Fisher LSD Method):

Comparisons for factor: plasmid				
Comparison	Diff of Means	LSD(alpha=0.050)	P	Diff >= LSD
full ADNP vs. p.Tyr719*	0.0455	0.0401	0.026	Yes
full ADNP vs. p.Arg730*	0.0346	0.0382	0.075	No

full ADNP vs. Control	0.00716	0.0491	0.774	Do Not Test
Control vs. p.Tyr719*	0.0383	0.0484	0.12	No
Control vs. p.Arg730*	0.0275	0.0469	0.25	Do Not Test
p.Arg730* vs. p.Tyr719*	0.0109	0.0373	0.566	Do Not Test
Comparisons for factor: treatment				
NAP treatment vs. w/o treatment	0.0335	0.0308	0.033	Yes
Comparisons for factor: treatment within Control				
w/o treatment vs. NAP treatment	0.0626	0.0793	0.122	No
Comparisons for factor: treatment within full ADNP				
NAP treatment vs. w/o treatment	0.0256	0.0578	0.385	No
Comparisons for factor: treatment within p.Arg730*				
NAP treatment vs. w/o treatment	0.0642	0.0499	0.012	Yes
Comparisons for factor: treatment within p.Tyr719*				
NAP treatment vs. w/o treatment	0.107	0.0555	<0.001	Yes
Comparisons for factor: plasmid within w/o treatment				
Control vs. p.Tyr719*	0.123	0.0512	<0.001	Yes
Control vs. p.Arg730*	0.0908	0.0473	<0.001	Yes
Control vs. full ADNP	0.0369	0.0507	0.154	No
full ADNP vs. p.Tyr719*	0.0862	0.0539	0.002	Yes
full ADNP vs. p.Arg730*	0.0539	0.0503	0.036	Yes
p.Arg730* vs. p.Tyr719*	0.0323	0.0507	0.211	No
Comparisons for factor: plasmid within NAP treatment				

full ADNP vs. Control	0.0512	0.084	0.231	No
full ADNP vs. p.Arg730*	0.0153	0.0574	0.6	Do Not Test
full ADNP vs. p.Tyr719*	0.00483	0.0593	0.873	Do Not Test
p.Tyr719* vs. Control	0.0464	0.0822	0.268	Do Not Test
p.Tyr719* vs. p.Arg730*	0.0105	0.0548	0.706	Do Not Test
p.Arg730* vs. Control	0.0359	0.0809	0.383	Do Not Test

"DF" - degrees of freedom, "SS" - sum of squares, "MS" - mean squares, "F" - F ratio, "P" - P value, "Do Not Test" –a comparison where no significant difference was found between the two means of the comparitors.

NAP increases Tau-MT interactions and protects MTs against degradation in the human neuroblastoma cells.

The following set of experiments were performed to confirm the effect of NAP on Tau-MT interaction in a human neuroblastoma SH-SY5Y cell line (clean of mycoplasma). SH-SY5Y cells were differentiated with retinoic acid before each experiment. Polymerized vs. soluble tubulin assay was performed to quantify tubulin polymerization and Tau-tubulin association under toxic condition of extracellular zinc (MT disrupter), and assess the recovering effect of NAP. After four hours with zinc (400µM) or NAP (10^{-12}M) or both together, the cellular-tubulin pool was separated into polymerized and soluble fractions (see "Supplemental Materials and Methods") and then analyzed by immunoblotting (Figure S12A). Exposure to extracellular zinc decreased Tau association with MTs by 2.5 fold and this reduction was fully prevented by NAP treatment (Fig. S12B). Zinc treatment resulted in a significant decrease in the polymerized tubulin, in comparison to non-treated control cells, indicating MT disassembly. NAP, added together with zinc, protected MTs against degradation (Fig. S12C). No effect was observed on the actin-microfilament pool

(Fig. S12D). NAP treatment alone had neither an effect on the MT pool (tubulin) nor on the Tau-MT association, similar to our previously published results[8, 9].

Further, we examined the effect of NAP on Tau-tubulin interaction by immunoprecipitation (IP) assay, using tubulin antibodies linked to agarose-beads. Protein lysate of differentiated SH-SY5Y cells were incubated with tubulin antibodies with and without NAP (see "Supplemental Materials and Methods"). The flow-through (F), first and third washes (W1 and W3), and elution (E) fractions were collected and analyzed by immunoblotting with Tau (IB: Tau) and tubulin (IB: tubulin) antibodies (Fig. S13A). Results showed that incubation with NAP increased Tau-tubulin association by ~ 5-fold (Fig. S13B).

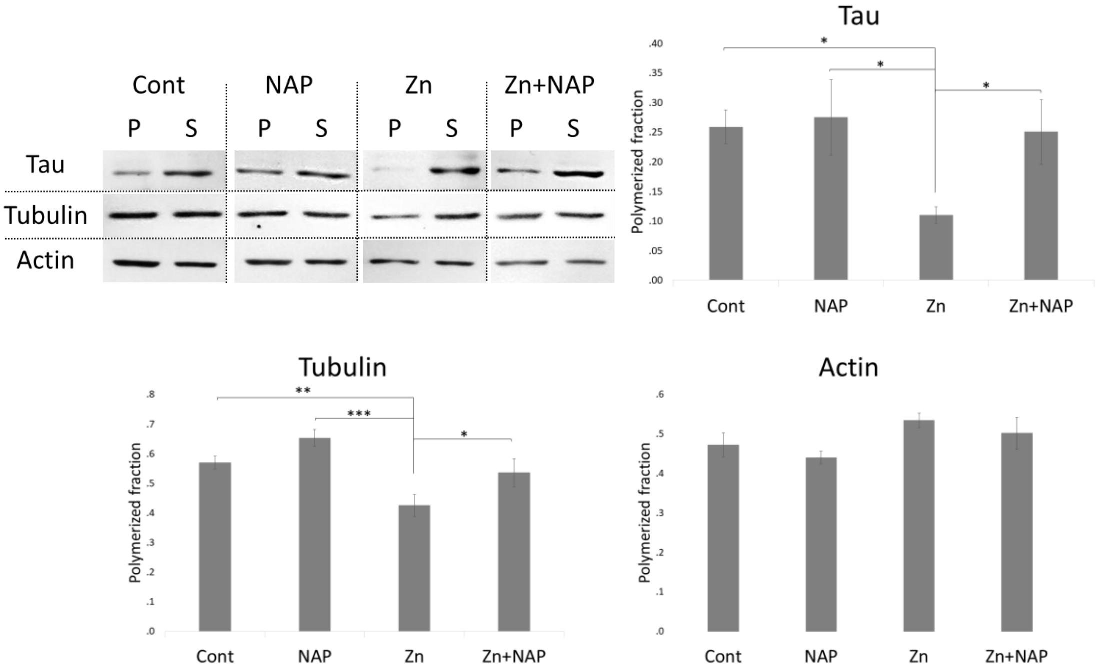

Supplemental Figure S12: NAP increases Tau-MT interactions and MT polymerization upon zinc intoxication in the human neuroblastoma cells. (A) Cell lysates of differentiated SH-SY5Y cells were separated into polymerized (P) and soluble (S) protein fractions after 4hrs of different cell treatments: without treatment (Cont), cells treated with NAP at 10^{-12}M (NAP) or zinc at 400µM (Zn) or both together (Zn+NAP). Aliquots of equal volumes for each pair (P and S) were resolved on adjacent lanes by SDS polyacrylamide gel electrophoresis as before[9]. The blots were probed with Tau, tubulin and actin antibodies. The intensity of each band was quantified by densitometry (C-D). The polymerized fraction was calculated by dividing the densitometric value of polymerized proteins by the total protein content (the sum of P plus S). Data were statistically analyzed by one-way ANOVA (n=9/group). * P<0.05, ** P<0.01, *** P<0.001.

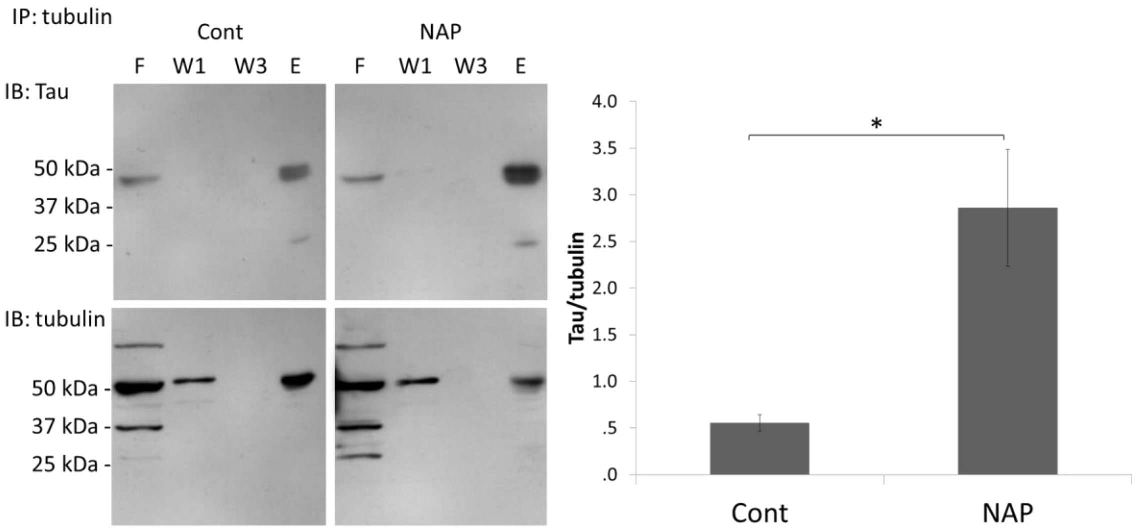

Supplemental Figure S13: NAP enhances Tau-tubulin association in human neuroblastoma cell model. (A) An immunoprecipitation (IP) assay was performed with tubulin antibodies, linked to agarose-beads. 2.3 µg of NAP (NAP), diluted in the lysis buffer or the equal volume of lysis buffer without NAP (Cont) were added to cell lysate of differentiated SH-SY5Y cells, and incubated with tubulin antibodies during 4 hrs.

Sequential IP flow-through (F), first (W1) and third (W3) washes, and elution (E) fractions were collected and further analyzed by immunoblotting with Tau (IB: Tau) and tubulin (IB: tubulin) antibodies. (B) Densitometric quantification of immunoreactive bands. The bar graph shows the ratio of immunoreactive bands of Tau compared to those of tubulin. Experiments were performed in duplicates and independently repeated 3 times. Data were statistically analyzed by Student's T-test, *$P<0.05$.

Statistical analysis

Polymerized vs. soluble tubulin assays were performed in triplicates and independently repeated three times. Statistical analysis of the data was performed by one-way ANOVA test (followed by the LSD post hoc test) by the IBM SPSS Statistics software version 23 (IBM, Armonk, NY, USA).

ANOVA						
		Sum of Squares	Df	Mean Square	F	Sig.
Tau	Between Groups	.144	3	.048	2.723	.062
	Within Groups	.530	30	.018		
	Total	.674	33			
tubulin	Between Groups	.225	3	.075	6.922	.001
	Within Groups	.336	31	.011		
	Total	.560	34			
actin	Between Groups	.044	3	.015	2.048	.127
	Within Groups	.229	32	.007		
	Total	.273	35			

Multiple Comparisons

LSD

Dependent Variable			Mean Difference (I-J)	Std. Error	Sig.	95% Confidence Interval	
						Lower Bound	Upper Bound
Tau	Cont	NAP	-.01604	.06263	.800	-.1439	.1119
		Zn	.14915*	.06456	.028	.0173	.2810
		Zn+NAP	.00850	.06456	.896	-.1233	.1403
	NAP	Cont	.01604	.06263	.800	-.1119	.1439
		Zn	.16519*	.06456	.016	.0333	.2970
		Zn+NAP	.02454	.06456	.707	-.1073	.1564
	Zn	Cont	-.14915*	.06456	.028	-.2810	-.0173
		NAP	-.16519*	.06456	.016	-.2970	-.0333
		Zn+NAP	-.14065*	.06643	.043	-.2763	-.0050
	Zn+NAP	Cont	-.00850	.06456	.896	-.1403	.1233
		NAP	-.02454	.06456	.707	-.1564	.1073
		Zn	.14065*	.06643	.043	.0050	.2763
tubulin	Cont	NAP	-.08312	.04905	.100	-.1832	.0169
		Zn	.14441*	.05056	.008	.0413	.2475
		Zn+NAP	.03374	.04905	.497	-.0663	.1338
	NAP	Cont	.08312	.04905	.100	-.0169	.1832
		Zn	.22753*	.05056	.000	.1244	.3307
		Zn+NAP	.11686*	.04905	.024	.0168	.2169
	Zn	Cont	-.14441*	.05056	.008	-.2475	-.0413

		NAP	-.22753*	.05056	.000	-.3307	-.1244
		Zn+NAP	-.11067*	.05056	.036	-.2138	-.0076
	Zn+NAP	Cont	-.03374	.04905	.497	-.1338	.0663
		NAP	-.11686*	.04905	.024	-.2169	-.0168
		Zn	.11067*	.05056	.036	.0076	.2138
actin	Cont	NAP	.03224	.03991	.425	-.0491	.1135
		Zn	-.06205	.03991	.130	-.1434	.0192
		Zn+NAP	-.02989	.03991	.459	-.1112	.0514
	NAP	Cont	-.03224	.03991	.425	-.1135	.0491
		Zn	-.09430*	.03991	.204	-.1756	-.0130
		Zn+NAP	-.06213	.03991	.129	-.1434	.0192
	Zn	Cont	.06205	.03991	.130	-.0192	.1434
		NAP	.09430*	.03991	.204	.0130	.1756
		Zn+NAP	.03216	.03991	.426	-.0491	.1135
	Zn+NAP	Cont	.02989	.03991	.459	-.0514	.1112
		NAP	.06213	.03991	.129	-.0192	.1434
		Zn	-.03216	.03991	.426	-.1135	.0491

*. The mean difference is significant at the 0.05 level.

Immunoprecipitation assay was performed in duplicates and independently repeated three times. Statistical analysis of the data was performed by independent samples T-test (equal variances not assumed) by the IBM SPSS Statistics software version 23 (IBM, Armonk, NY, USA).

Group Statistics					
Dif		N	Mean	Std. Deviation	Std. Error Mean
IP	Cont	5	.5567	.19805	.08857
	NAP	5	2.8617	1.39302	.62298

Independent Samples Test (IP results)

	Levene's Test for Equality of Variances		t-test for Equality of Means						
								95% Confidence Interval of the Difference	
	F	Sig.	t	df	Sig. (2-tailed)	Mean Difference	Std. Error Difference	Lower	Upper
Equal variances assumed	13.235	.007	-3.663	8	.006	-2.30505	.62924	-3.75609	-.85402
Equal variances not assumed			-3.663	4.162	.020	-2.30505	.62924	-4.02567	-.58444

Discussion

Accumulating mutations in aging and AD could be a results of abnormal DNA repair mechanism **(Supplemental Table S15 – excel file -** Shared potential disease-driving genes mutations with DNA repair genes: GO:0006281**).** For example**,** Mutations in the adult progeria gene (Warner syndrome) ATP-dependent helicase (WRN) were also discovered in fusiform gyrus subjects (Table S15). Notably, base excision repair (the primary DNA repair pathway for small base modifications such as alkylation, deamination and oxidation)

was found to be defective in AD brains[26]. Finally, mitochondrial DNA damage has been implicated in AD[27] and our findings here of a major impact on the cytoskeletal system are directly associated with mitochondrial transport[28].

Supplemental Movies: Expression of truncated forms of ADNP decreased speed and track length of end-binding protein 3 (EB3) and NAP treatment restored EB3 action in the living cells. Live imaging of differentiated N1E-115 cells co-transfected with plasmids expressing EB3-RFP (red) and one of the truncated forms of ADNP conjugated to GFP (not shown in the movies, Supplemental Figure S9) with or without NAP treatment (10^{-12}M, 4hrs). Time-lapse images were automatically captured every 3 sec during a 1min period using the Leica LAS AF software.

Movie S1: N1E-115 cells transfected with EB3-RFP and GFP-control plasmid (pEGFP-C1).

Movies S2: N1E-115 cells transfected with EB3-RFP and GFP-C1-ADNP (full-length) w/o NAP treatment (**Movie S2A**) or with NAP treatment (**Movie S2B**).

Movies S3: N1E-115 cells transfected with EB3-RFP and GFP-C1-mutADNP-p.Arg730* w/o NAP treatment (**Movie S3A**) or with NAP treatment (**Movie S3B**).

Movies S4: N1E-115 cells transfected with EB3-RFP and GFP-C1-mutADNP-p.Tyr719* w/o NAP treatment (**Movie S4A**) or with NAP treatment (**Movie S4B**).

References:

1. Helsmoortel C, Vulto-van Silfhout AT, Coe BP, Vandeweyer G, Rooms L, van den Ende J, et al. A SWI/SNF-related autism syndrome caused by de novo mutations in ADNP. *Nat Genet*. 2014 Apr;46(4):380-4.

2. Felix-Urquidez D, Perez-Urquiza M, Valdez Torres JB, Leon-Felix J, Garcia-Estrada R, Acatzi-Silva A. Development, Optimization, and Evaluation of a Duplex Droplet Digital PCR Assay To Quantify the T-nos/hmg Copy Number Ratio in Genetically Modified Maize. *Analytical chemistry*. 2016 Jan 5;88(1):812-9.

3. Afgan E, Baker D, Van den Beek M, Blankenberg D, Bouvier D, Čech M, et al. The Galaxy platform for accessible, reproducible and collaborative biomedical analyses: 2016 update. *Nucleic acids research*. 2016;44(W1):W3-W10.

4. Leinonen R, Sugawara H, Shumway M, Collaboration INSD. The sequence read archive. *Nucleic acids research*. 2010;39(suppl_1):D19-D21.

5. Dobin A, Davis CA, Schlesinger F, Drenkow J, Zaleski C, Jha S, et al. STAR: ultrafast universal RNA-seq aligner. *Bioinformatics*. 2013;29(1):15-21.
6. Szklarczyk D, Morris JH, Cook H, Kuhn M, Wyder S, Simonovic M, et al. The STRING database in 2017: quality-controlled protein–protein association networks, made broadly accessible. *Nucleic acids research*. 2016:gkw937.
7. Gozes I, Hoglinger G, Quinn JP, Hooper NM, Hoglund K. Tau Diagnostics and Clinical Studies. *Journal of molecular neuroscience : MN*. 2017 Oct;63(2):123-30.
8. Oz S, Ivashko-Pachima Y, Gozes I. The ADNP derived peptide, NAP modulates the tubulin pool: implication for neurotrophic and neuroprotective activities. *PloS one*. 2012;7(12):e51458.
9. Ivashko-Pachima Y, Sayas CL, Malishkevich A, Gozes I. ADNP/NAP dramatically increase microtubule end-binding protein-Tau interaction: a novel avenue for protection against tauopathy. *Molecular psychiatry*. 2017 Sep;22(9):1335-44.
10. Ivashko-Pachima Y, Gozes I. NAP Protects against Tau Hyperphosphorylation Through GSK3. *Current pharmaceutical design*. 2018;24(33):3868-77.
11. Gozes I, Barnstable CJ. Monoclonal antibodies that recognize discrete forms of tubulin. *Proceedings of the National Academy of Sciences of the United States of America*. 1982 Apr;79(8):2579-83.
12. Thal DR, Rub U, Orantes M, Braak H. Phases of A beta-deposition in the human brain and its relevance for the development of AD. *Neurology*. 2002 Jun 25;58(12):1791-800.
13. Bustin SA, Benes V, Garson JA, Hellemans J, Huggett J, Kubista M, et al. Primer sequence disclosure: a clarification of the MIQE guidelines. *Clinical chemistry*. 2011 Jun;57(6):919-21.
14. Schiavo G, Greensmith L, Hafezparast M, Fisher EM. Cytoplasmic dynein heavy chain: the servant of many masters. *Trends in neurosciences*. 2013 Nov;36(11):641-51.
15. Iossifov I, O'Roak BJ, Sanders SJ, Ronemus M, Krumm N, Levy D, et al. The contribution of de novo coding mutations to autism spectrum disorder. *Nature*. 2014 Nov 13;515(7526):216-21.
16. De Rubeis S, He X, Goldberg AP, Poultney CS, Samocha K, Cicek AE, et al. Synaptic, transcriptional and chromatin genes disrupted in autism. *Nature*. 2014 Nov 13;515(7526):209-15.
17. Pereira AC, Gray JD, Kogan JF, Davidson RL, Rubin TG, Okamoto M, et al. Age and Alzheimer's disease gene expression profiles reversed by the glutamate modulator riluzole. *Molecular psychiatry*. 2017 Feb;22(2):296-305.
18. Kim JH. Genetics of Alzheimer's Disease. *Dementia and neurocognitive disorders*. 2018 Dec;17(4):131-6.
19. Wang ZX, Wan Y, Tan L, Liu J, Wang HF, Sun FR, et al. Genetic Association of HLA Gene Variants with MRI Brain Structure in Alzheimer's Disease. *Molecular neurobiology*. 2017 Jul;54(5):3195-204.
20. Bennabi M, Gaman A, Delorme R, Boukouaci W, Manier C, Scheid I, et al. HLA-class II haplotypes and Autism Spectrum Disorders. *Scientific reports*. 2018 May 16;8(1):7639.
21. Mufson EJ, He B, Ginsberg SD, Carper BA, Bieler GS, Crawford F, et al. Gene Profiling of Nucleus Basalis Tau Containing Neurons in Chronic Traumatic Encephalopathy: A Chronic Effects of Neurotrauma Consortium Study. *Journal of neurotrauma*. 2018 Jun 1;35(11):1260-71.
22. Li L, Liu Y, Li S, Yang Y, Zeng C, Rong W, et al. Signal regulatory protein alpha protects podocytes through promoting autophagic activity. *JCI insight*. 2019 Mar 19;5.
23. Srinivasan K, Friedman BA, Etxeberria A, Huntley MA, Van Der Brug MP, Foreman O, et al. Alzheimer's patient brain myeloid cells exhibit enhanced aging and unique transcriptional activation. *BioRxiv*. 2019:610345.

24. Vaisburd S, Shemer Z, Yeheskel A, Giladi E, Gozes I. Risperidone and NAP protect cognition and normalize gene expression in a schizophrenia mouse model. *Scientific reports*. 2015 Nov 10;5:16300.
25. Mandel S, Gozes I. Activity-dependent neuroprotective protein constitutes a novel element in the SWI/SNF chromatin remodeling complex. *The Journal of biological chemistry*. 2007 Nov 23;282(47):34448-56.
26. Weissman L, Jo DG, Sorensen MM, de Souza-Pinto NC, Markesbery WR, Mattson MP, et al. Defective DNA base excision repair in brain from individuals with Alzheimer's disease and amnestic mild cognitive impairment. *Nucleic Acids Res*. 2007;35(16):5545-55.
27. Wang J, Xiong S, Xie C, Markesbery WR, Lovell MA. Increased oxidative damage in nuclear and mitochondrial DNA in Alzheimer's disease. *Journal of neurochemistry*. 2005 May;93(4):953-62.
28. Shprung T, Gozes I. A novel method for analyzing mitochondrial movement: inhibition by paclitaxel in a pheochromocytoma cell model. *Journal of molecular neuroscience : MN*. 2009 Mar;37(3):254-62.
