# Supplementary material for: Discovery of autism/intellectual disability somatic mutations in Alzheimer's brains: mutated ADNP cytoskeletal impairments and repair as a case study
Source: Mol Psychiatry. 2019 Oct 30;26(5):1619–33. doi: 10.1038/s41380-019-0563-5 (PMC8159740; doi:10.1038/s41380-019-0563-5)
Supplement: Supplementary file 2 — Supplemental Figure S7 [file 41380_2019_563_MOESM2_ESM.pdf]

Supplemental Figure S7: String analysis of interacting mutated proteins

Olfactory bulb – AD only genes - OMIM

https://string-db.org/cgi/network.pl?taskId=PWIm8F5H6sqP

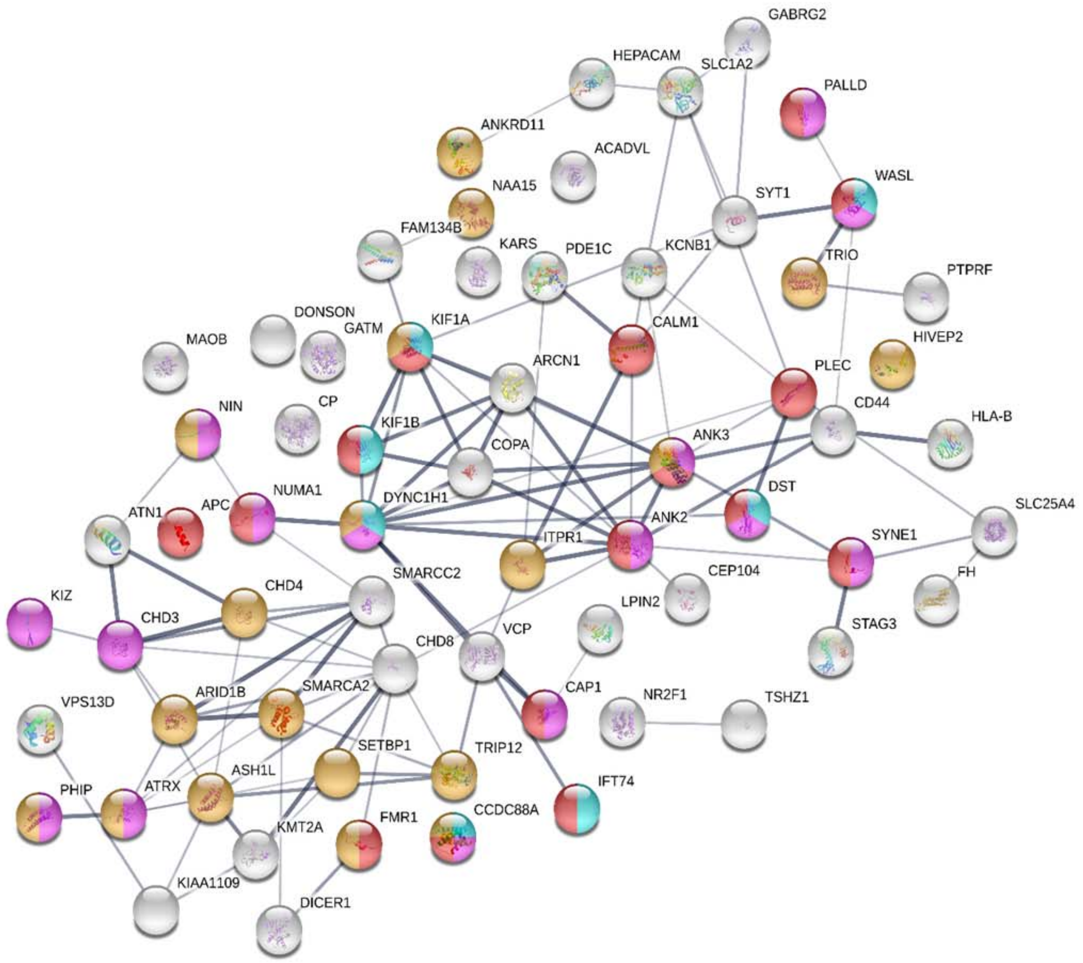

Biological Process (GO)

cytoskeleton-dependent intracellular transport

cytoskeleton organization

Molecular Function (GO)

cytoskeletal protein binding

Intellectual disability (ID) (mental retardation)

Biological Process (GO)					
GO-term	GO:0030705	GO:0007010	GO:0006996	GO:0016043	GO:0007017
description	cytoskeleton-dependent intracellular transport	cytoskeleton organization	organelle organization	cellular component organization	microtubule-based process
count in gene set	7 of 151	15 of 953	27 of 3131	35 of 5163	11 of 605
false discovery rate	0.0012	0.0012	0.0012	0.0022	0.0026
Molecular Function (GO)					
GO-term	GO:0008092	GO:0008094	GO:0140030	GO:0016887	GO:0004386
description	cytoskeletal protein binding	DNA-dependent ATPase activity	modification-dependent protein binding	ATPase activity	helicase activity
count in gene s	16 of 882	5 of 66	6 of 131	9 of 392	6 of 147
false discovery	0.0000134	0.0008	0.0009	0.0009	0.001
UniProt Keywords					
keyword	KW-9995	KW-0225	KW-0991	KW-0597	KW-0206
description	Disease	Disease mutation	Mental retardation	Phosphoprotein	Cytoskeleton
count in gene s	54 of 3799	41 of 2951	19 of 415	58 of 8066	19 of 1198
false discovery	3.62E-25	5E-16	1E-14	2E-13	4E-07

Olfactory bulb – Control only genes - OMIM

https://string-db.org/cgi/network.pl?ta skId=6UMaBVm4UBzf

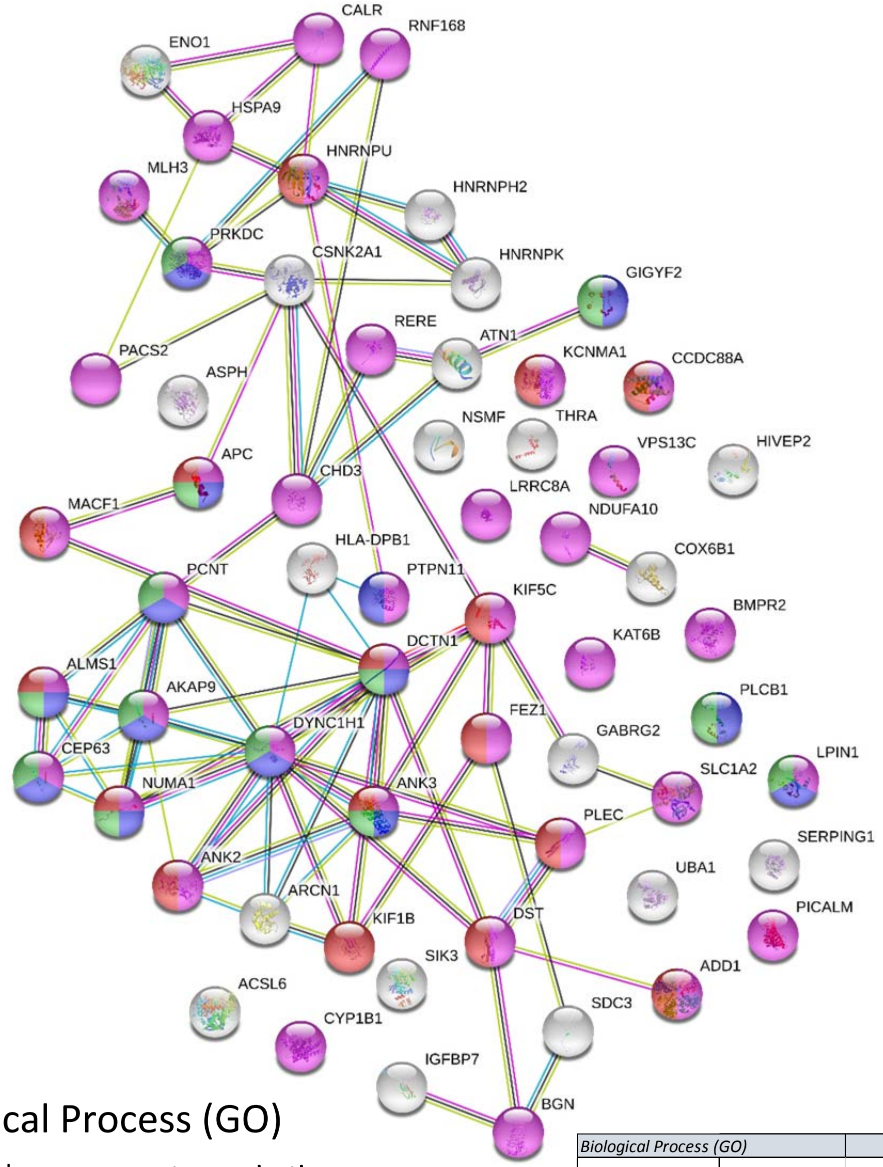

Biological Process (GO)

- cellular component organization
- Mitotic cell cycle
- Mitotic cell cycle process

Molecular Function (GO)

- cytoskeletal protein binding

Biological Process (GO)					
GO-term	GO:0016043	GO:0000278	GO:1903047	GO:0051179	GO:0051128
description	cellular component organization	mitotic cell cycle	mitotic cell cycle process	localization	regulation of cellular component organization
count in gene set	38 of 5163	14 of 628	13 of 564	37 of 5233	24 of 2306
false discovery rate	0.00000556	0.00000728	0.00000941	0.00000941	0.00000941
Molecular Function (GO)					
GO-term	GO:0008092	GO:0005515	GO:0015631	GO:0008017	GO:0051010
description	cytoskeletal protein binding	protein binding	tubulin binding	microtubule binding	microtubule plus-end binding
count in gene s	16 of 882	42 of 6605	9 of 344	8 of 253	3 of 13
false discovery	0.00000281	0.00000281	0.00015	0.00015	0.0011
UniProt Keywords					
keyword	KW-9995	KW-0206	KW-0225	KW-0597	KW-0493
description	Disease	Cytoskeleton	Disease mutation	Phosphoprotein	Microtubule
count in gene s	44 of 3799	21 of 1198	31 of 2951	49 of 8066	10 of 275
false discovery	5.59E-17	2.82E-09	2.83E-09	8.21E-09	0.000000538

Hippocampus – AD, AD+Control genes – OMIM 106 genes

https://string-db.org/cgi/network.pl?taskId=ubCYgdBAAUK2

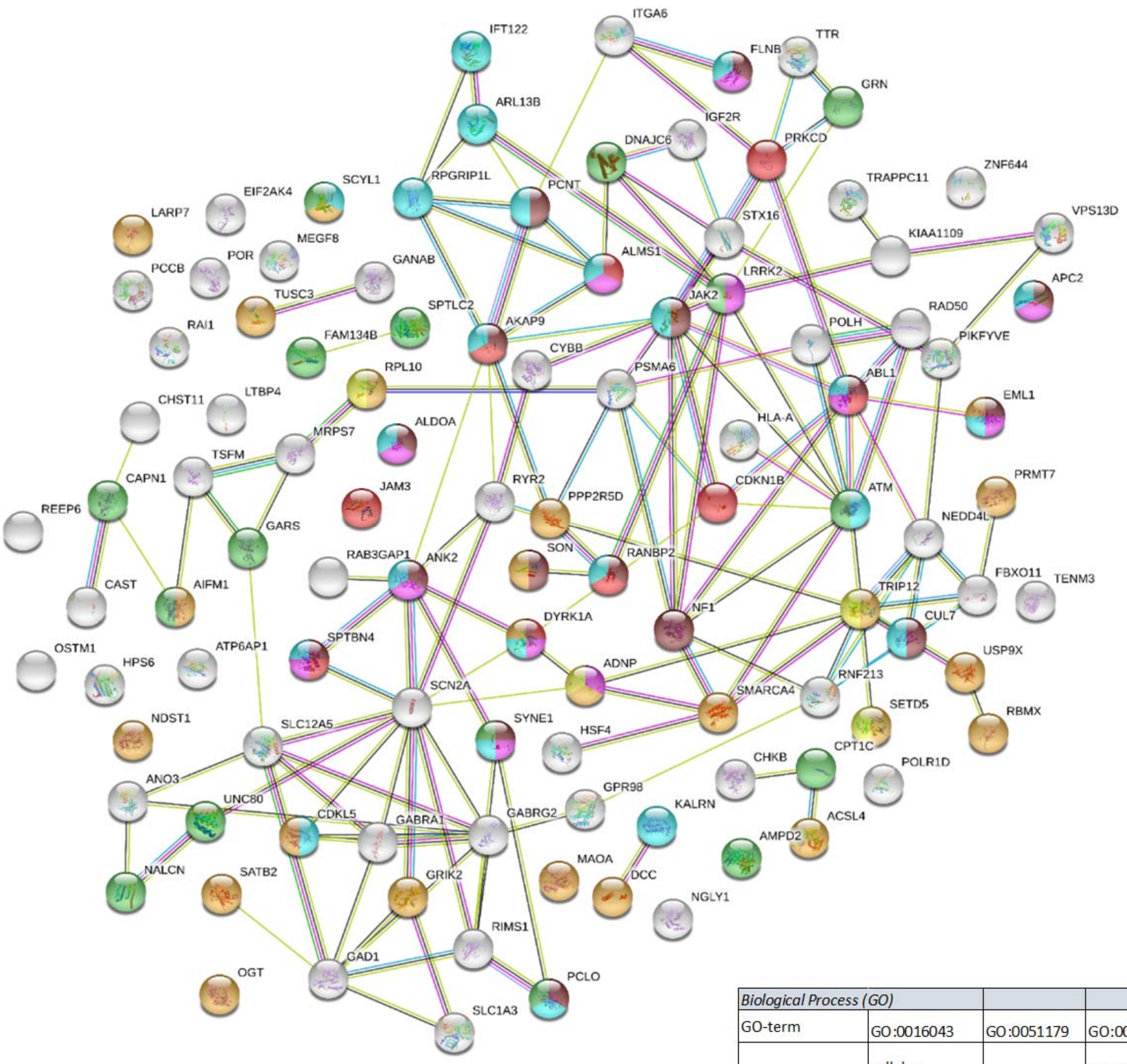

Molecular Function
Biological Process
Cellular Component
} Cytoskeleton
Neurodegeneration
Autism spectrum disorder
Intellectual disability (ID) (mental retardation)

Biological Process (GO)					
GO-term	GO:0016043	GO:0051179	GO:0007399	GO:0007010	GO:0051493
description	cellular component organization	localization	nervous system development	cytoskeleton organization	regulation of cytoskeleton organization
count in gene set	61 of 5163	59 of 5233	36 of 2206	16 of 953	9 of 477
false discovery rate	5.11E-08	0.0000004	0.000000566	0.0031	0.019
Molecular Function (GO)					*Cellular Component (GO)*
GO-term	*GO:0043168*	*GO:0017016*	*GO:0016773*	*GO:0008092*	*GO:0005856*
description	*anion binding*	*Ras GTPase binding*	*phosphotransferase activity, alcohol group as acceptor*	*cytoskeletal protein binding*	*cytoskeleton*
count in gene set	*32 of 2696*	*12 of 510*	*15 of 767*	*12 of 882*	*23 of 2068*
false discovery rate	*0.0018*	*0.0018*	*0.0018*	*0.0452*	*0.0073*
UniProt Keywords					
keyword	KW-9995	KW-0991	KW-0523	KW-0622	KW-1268
description	Disease	Mental retardation	Neurodegeneration	Neuropathy	Autism spectrum disorder
count in gene set	90 of 3799	24 of 415	16 of 334	6 of 103	4 of 43
false discovery rate	6.23E-45	8.1E-16	4.18E-09	0.0011	0.0036

Hippocampus – AD only genes – OMIM 40 genes
https://string-db.org/cgi/network.pl?taskId=Oiy2cVbkL6Ya

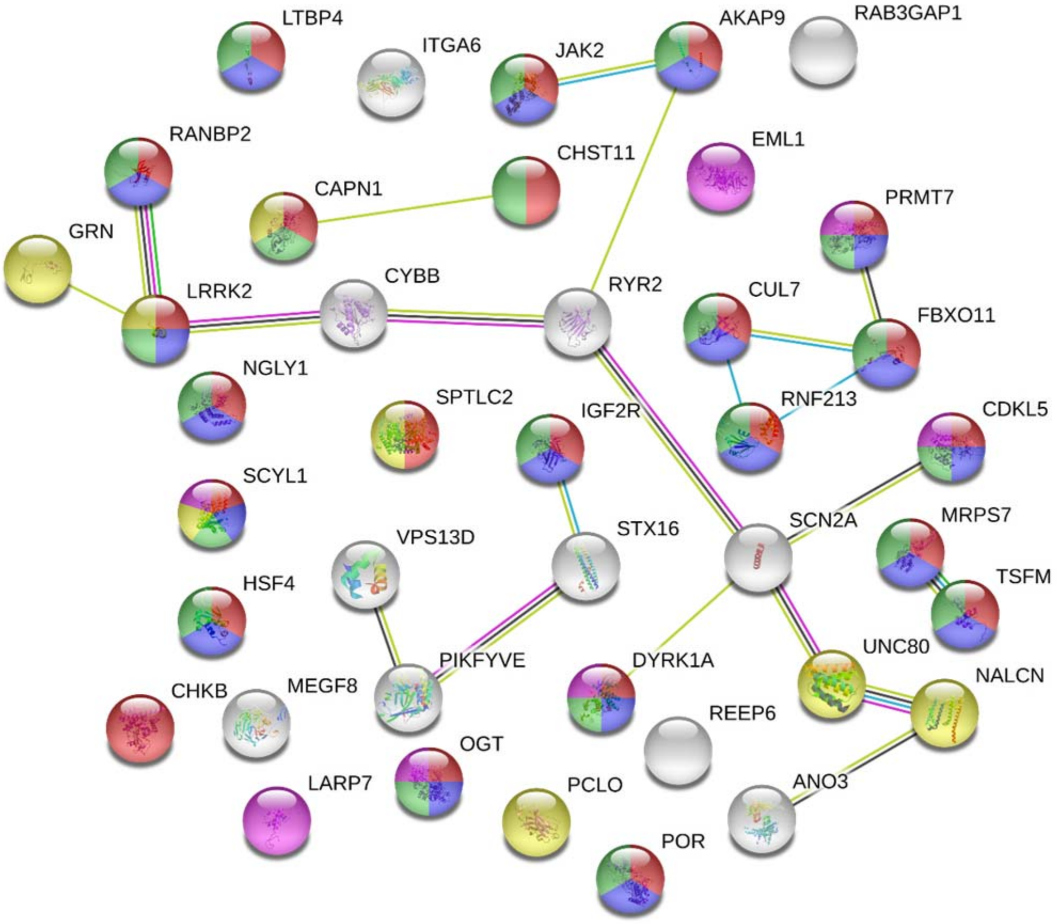

- cellular protein metabolic process
- protein metabolic process
- organonitrogen compound metabolic process
- Neurodegeneration
- Intellectual disability (ID) (mental retardation)

Biological Process (GO)					
GO-term	GO:0018193	GO:1901564	GO:0051179	GO:0044267	GO:0019538
description	peptidyl-amino acid modification	organonitrogen compound metabolic process	localization	cellular protein metabolic process	protein metabolic process
count in gene set	10 of 842	23 of 5281	23 of 5233	19 of 3603	21 of 4194
false discovery rate	0.0109	0.0149	0.0149	0.0149	0.0149
UniProt Keywords					
keyword	KW-9995	KW-0225	KW-0523	KW-0991	KW-0597
description	Disease	Disease mutation	Neurodegeneration	Mental retardation	Phosphoprotein
count in gene set	31 of 3799	25 of 2951	8 of 334	7 of 415	29 of 8066
false discovery rate	6.11E-13	9.32E-10	0.0000193	0.0008	0.0019

Hippocampus – Control only genes – OMIM 50 genes

https://string-db.org/cgi/network.pl?taskId=HuorAJRhjQpQ

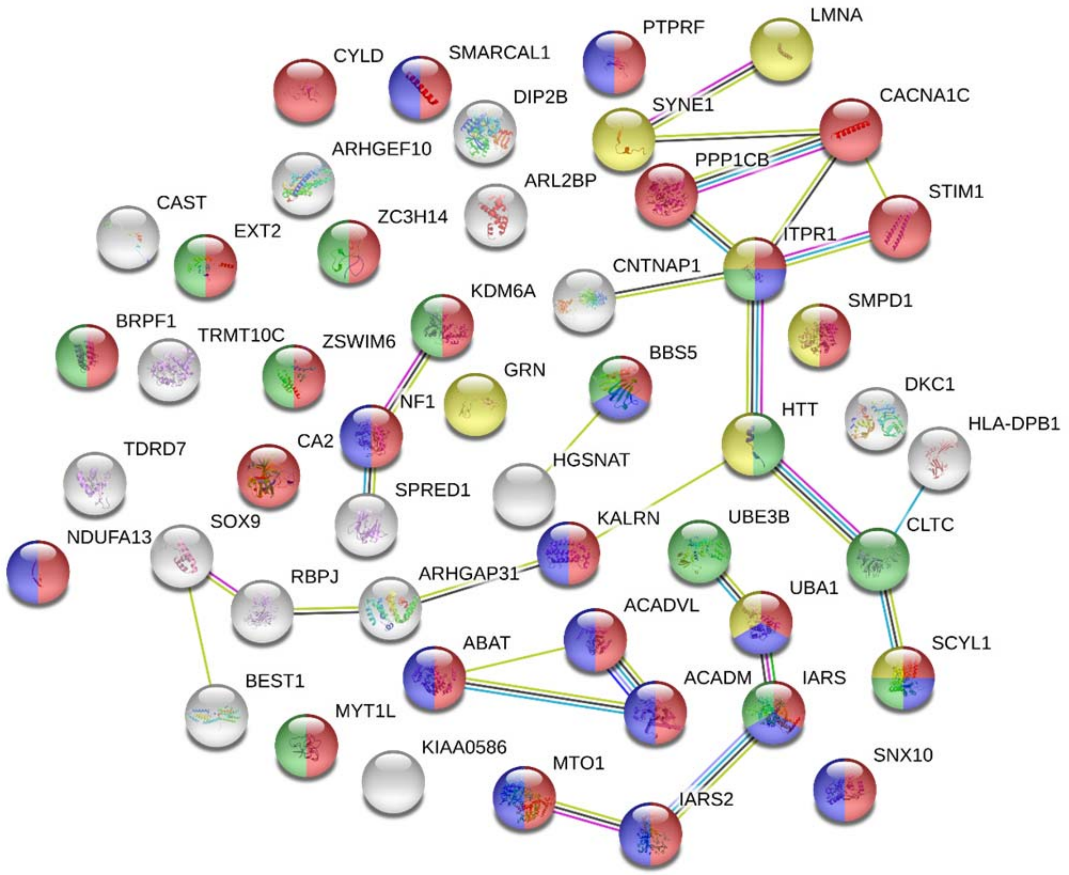

Anion binding

Ion binding

Neurodegeneration

Intellectual disability (ID) (mental retardation)

Molecular Function (GO)					
GO-term	GO:0004822	GO:0043167	GO:0002161	GO:0000049	GO:0043168
description	isoleucine-tRNA ligase activity	ion binding	aminoacyl-tRNA editing activity	tRNA binding	anion binding
count in gene set	2 of 2	28 of 6066	2 of 12	3 of 56	16 of 2696
false discovery rate	0.0073	0.0248	0.0386	0.0386	0.0446
UniProt Keywords					
keyword	KW-9995	KW-0225	KW-0991	KW-0523	KW-0597
description	Disease	Disease mutation	Mental retardation	Neurodegeneration	Phosphoprotein
count in gene set	44 of 3799	38 of 2951	13 of 415	8 of 334	36 of 8066
false discovery rate	4.68E-23	1.18E-19	2.16E-09	0.0000999	0.00038

Dorsolateral prefrontal cortex (DLPFC) –AD, AD+Control OMIM- 256 genes

https://string-db.org/cgi/network.pl?taskId=p2PV9wDzcXvG

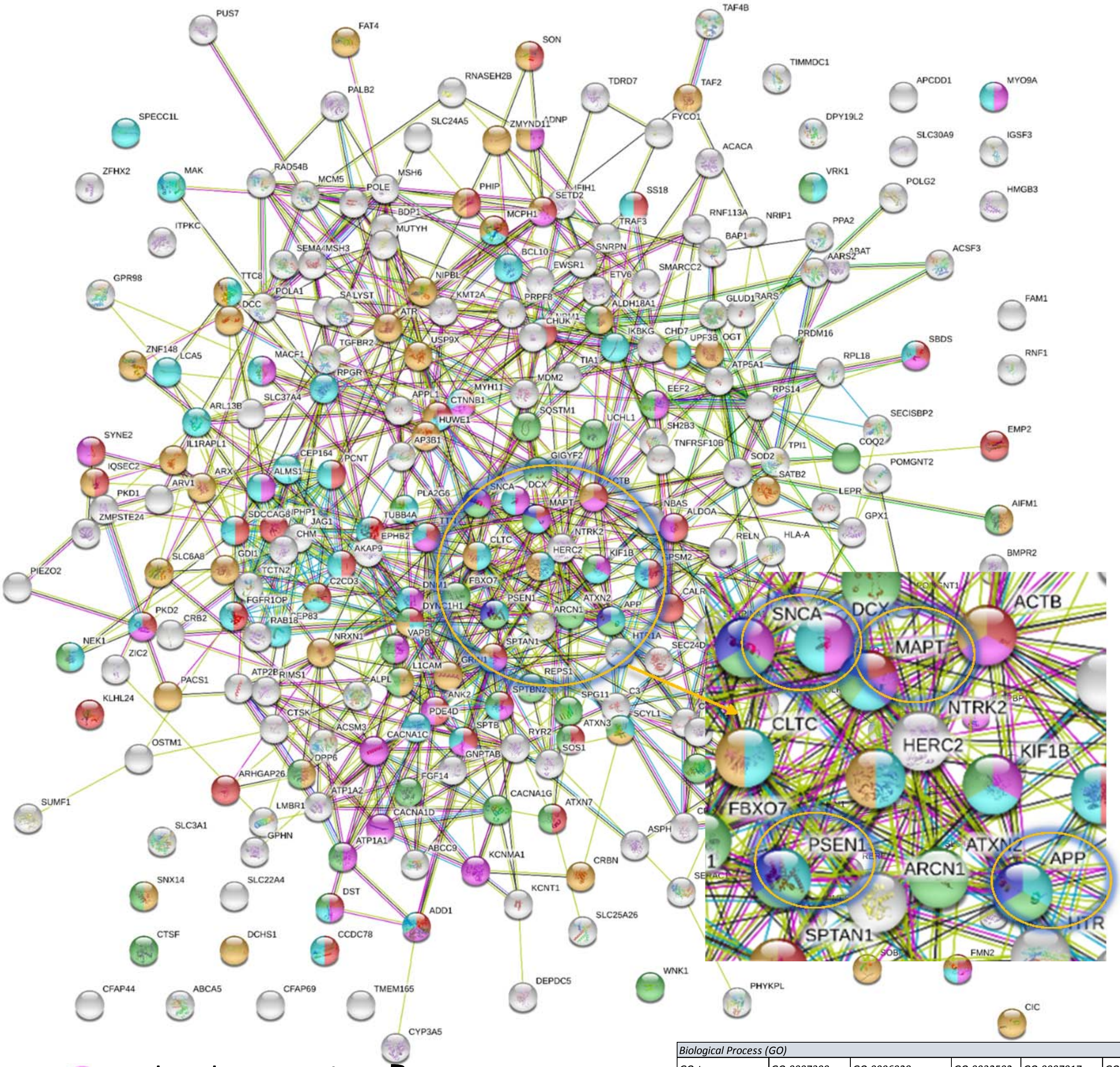

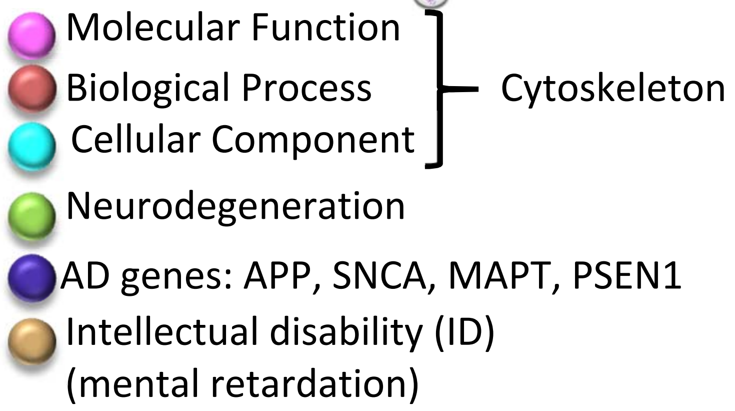

Biological Process (GO)					
GO-term	*GO:0007399*	*GO:0006928*	*GO:0032502*	*GO:0007017*	*GO:0007010*
description	*nervous system development*	*movement of cell or subcellular component*	*developmental process*	*microtubule-based process*	*cytoskeleton organization*
count in gene set	*73 of 2206*	*55 of 1355*	*126 of 5401*	*33 of 605*	*40 of 953*
false discovery rate	*4.46E-11*	*4.86E-11*	*1.36E-10*	*0.000000004*	*3.44E-08*
Molecular Function (GO)					
GO-term	GO:0043167	GO:0019899	GO:0019901	GO:0019900	GO:0008092
description	*ion binding*	*enzyme binding*	*protein kinase binding*	*kinase binding*	*cytoskeletal protein binding*
count in gene s	*125 of 6066*	*61 of 2197*	*27 of 599*	*28 of 678*	*32 of 882*
false discovery	*0.000000704*	*0.00000272*	*0.00000744*	*0.0000197*	*0.0000347*
UniProt Keywords					
keyword	KW-0991	KW-0523	KW-0206	KW-0907	KW-0026
description	Mental retardation	Neurodegeneration	Cytoskeleton	Parkinson disease	Alzheimer disease
count in gene s	45 of 415	35 of 334	46 of 1198	5 of 23	4 of 17
false discovery	1.48E-24	1.99E-18	3.76E-09	0.00039	0.0016

Dorsolateral prefrontal cortex (DLPFC) – AD only genes OMIM- 159 genes

https://string-db.org/cgi/network.pl?taskId=IQej399UKSiY

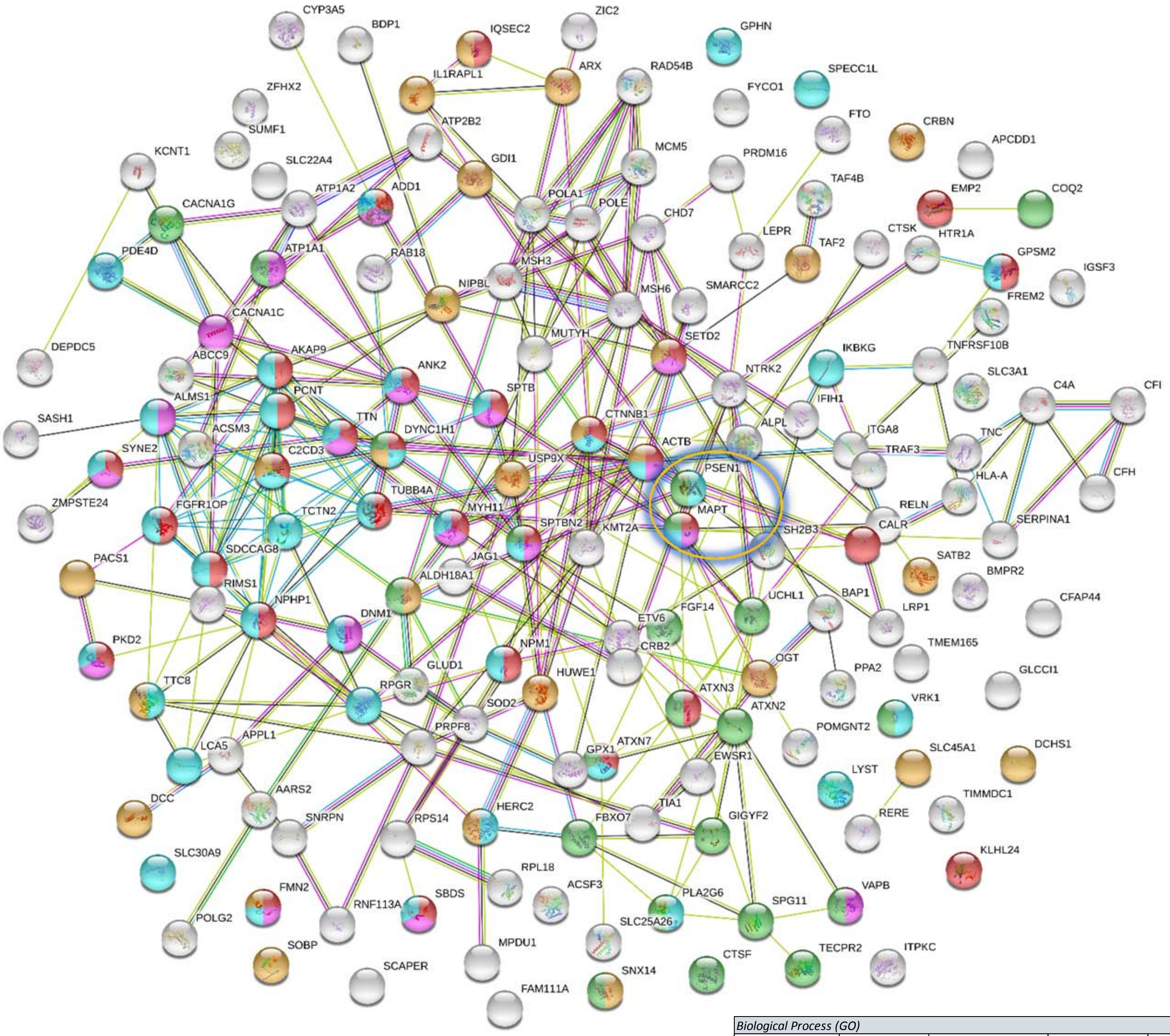

- Molecular Function
- Biological Process
- Cellular Component

} Cytoskeleton

- Neurodegeneration
- Intellectual disability (ID) (mental retardation)

Biological Process (GO)					
GO-term	GO:0007010	GO:0051641	GO:0051640	GO:0007017	GO:0007399
description	cytoskeleton organization	cellular localization	organelle localization	microtubule-based process	nervous system development
count in gene set	30 of 953	47 of 2180	23 of 574	23 of 605	45 of 2206
false discovery rate	0.000000136	0.000000138	0.000000211	0.000000503	0.00000154
Molecular Function (GO)				*Cellular Component (GO)*	
GO-term	GO:0044325	GO:0003682	GO:0008092	GO:0015630	GO:0005856
description	ion channel binding	chromatin binding	cytoskeletal protein binding	microtubule cytoskeleton	cytoskeleton
count in gene set	8 of 120	15 of 501	18 of 882	28 of 1118	40 of 2068
false discovery rate	0.0019	0.0019	0.0133	0.0000105	0.0000105
UniProt Keywords					
keyword	KW-0991	KW-0523	KW-0597	KW-0206	KW-0908
description	Mental retardation	Neurodegeneration	Phosphoprotein	Cytoskeleton	Parkinsonism
count in gene set	26 of 415	22 of 334	109 of 8066	33 of 1198	7 of 37
false discovery rate	1.77E-13	9.02E-12	2.21E-10	3.72E-08	0.00000234

Dorsolateral prefrontal cortex (DLPFC) – Control only genes OMIM- 159 genes https://string-db.org/cgi/network.pl?taskId=5szFpyvFTsld

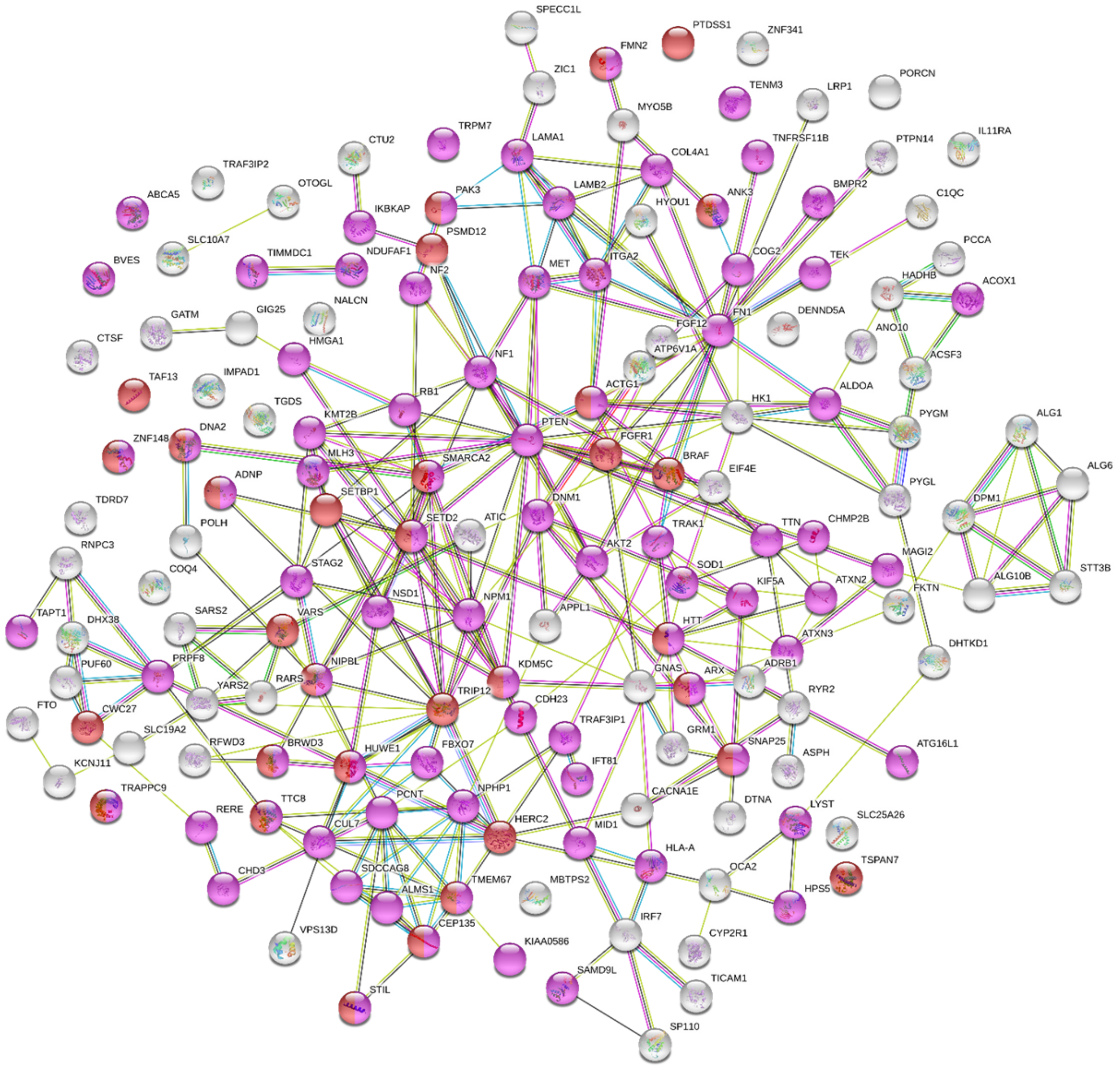

Cellular component organization

Intellectual disability (ID) (mental retardation)

Biological Process (GO)				
GO-term	*GO:0016043*	*GO:0051179*	*GO:0051641*	*GO:0006996*
description	*cellular component organization*	*localization*	*cellular localization*	*organelle organization*
count in gene set	*80 of 5163*	*80 of 5233*	*44 of 2180*	*55 of 3131*
false discovery rate	*0.000000254*	*0.00000034*	*0.00000696*	*0.00000696*
UniProt Keywords				
keyword	KW-9995	KW-0225	KW-0991	KW-0597
description	*Disease*	*Disease mutation*	*Mental retardation*	*Phosphoprotein*
count in gene set	*131 of 3799*	*113 of 2951*	*32 of 415*	*108 of 8066*
false discovery rate	*2.31E-62*	*1.4E-53*	*1.95E-19*	*8.43E-10*

AD-specific cytoskeleton –
https://string-db.org/cgi/network.pl?taskId=4JDIDlcn4186
(Table S8b)

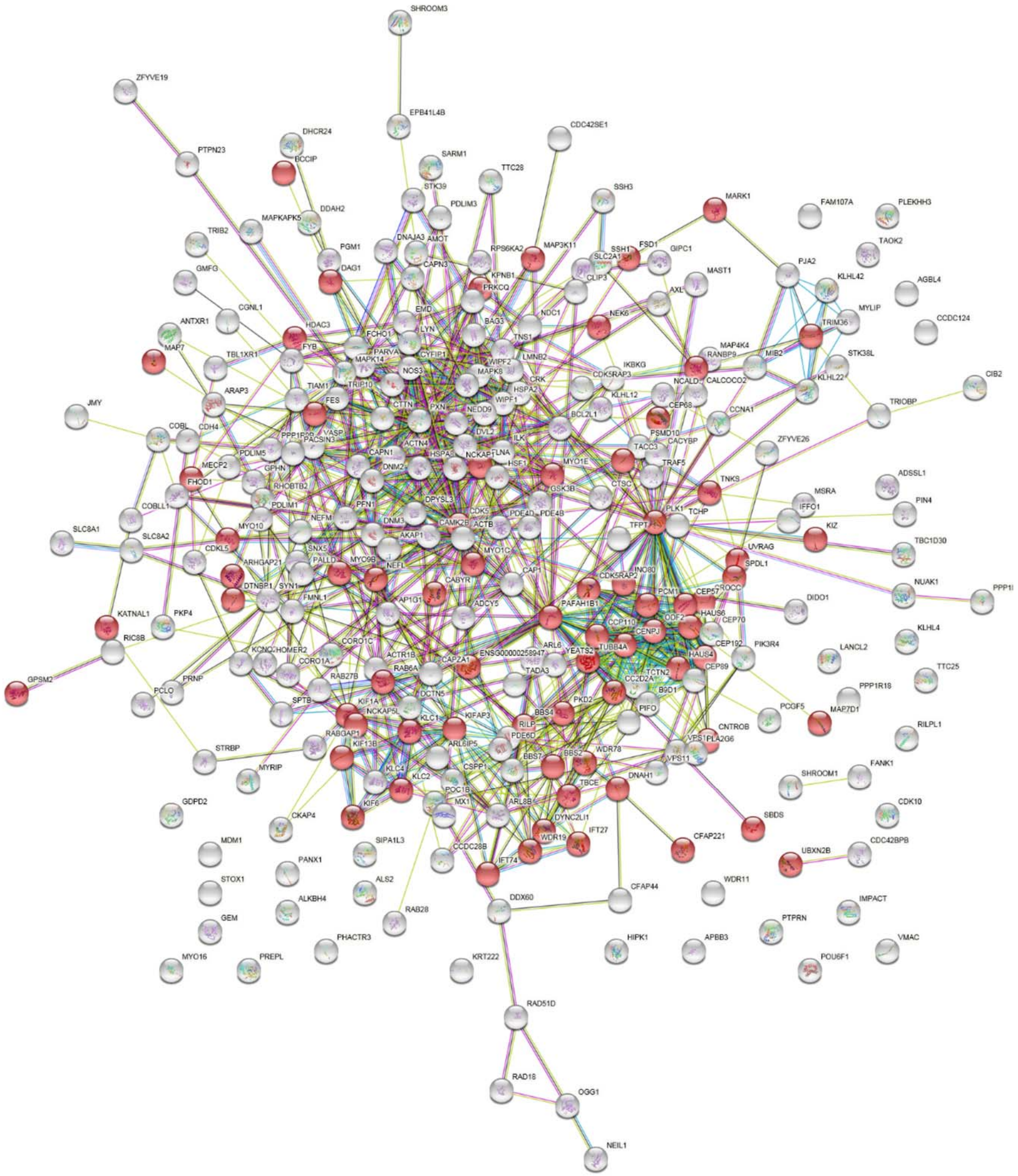

Network Stats

number of nodes:	274	expected number of edges:	385
number of edges:	912	PPI enrichment p-value:	< 1.0e-16
average node degree:	6.66		
avg. local clustering coefficient:	0.384		

your network has significantly more interactions than expected (what does that mean?)

Functional enrichments in your network

Biological Process (GO)

GO-term	*description*	*count in gene set*	*false discovery rate*
GO:0007010	cytoskeleton organization	86 of 953	4.98e-40
GO:0007017	microtubule-based process	70 of 605	4.05e-38
GO:0006996	organelle organization	133 of 3131	2.93e-32
GO:0016043	cellular component organization	171 of 5163	3.09e-32
GO:0071840	cellular component organization or biogenesis	173 of 5342	1.11e-31

[illegible]

Control-specific cytoskeleton – https://string-db.org/cgi/network.pl?taskId=dQGYgLlZAZUl (Table S8b)

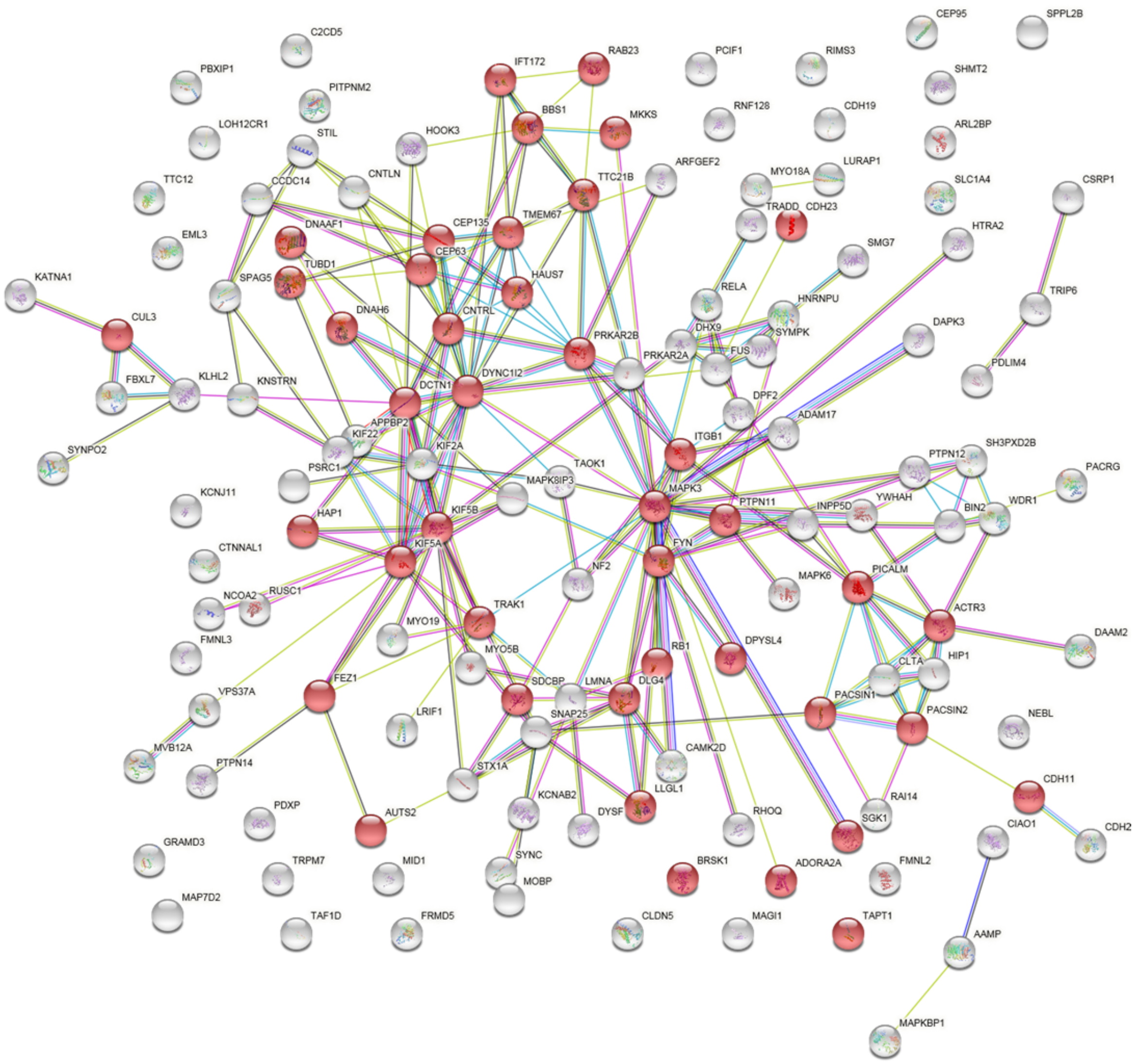

Network Stats

number of nodes:	139	expected number of edges:	117
number of edges:	230	PPI enrichment p-value:	< 1.0e-16
average node degree:	3.31	*your network has significantly more interactions than expected (what does that mean?)*	
avg. local clustering coefficient:	0.469		

Functional enrichments in your network

Biological Process (GO)			
GO-term	*description*	*count in gene set*	*false discovery rate*
GO:0016043	cellular component organization	95 of 5163	3.04e-21
GO:0071840	cellular component organization or biogenesis	96 of 5342	3.80e-21
GO:0006996	organelle organization	73 of 3131	6.43e-20
GO:0007010	cytoskeleton organization	42 of 953	7.34e-19
GO:0030030	cell projection organization	42 of 1067	3.44e-17
			(more ...)
