# Supplementary figures and images for: Discovery of autism/intellectual disability somatic mutations in Alzheimer's brains: mutated ADNP cytoskeletal impairments and repair as a case study
Source: Mol Psychiatry. 2019 Oct 30;26(5):1619–33. doi: 10.1038/s41380-019-0563-5 (PMC8159740; doi:10.1038/s41380-019-0563-5)

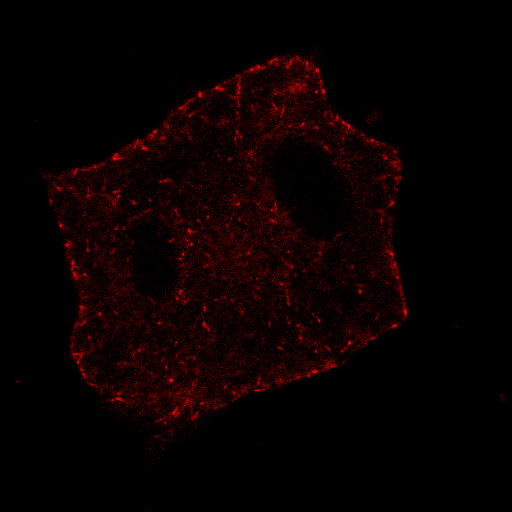

Supplement: Supplementary file 3 — Movie S1 [file 41380_2019_563_MOESM3_ESM.gif]

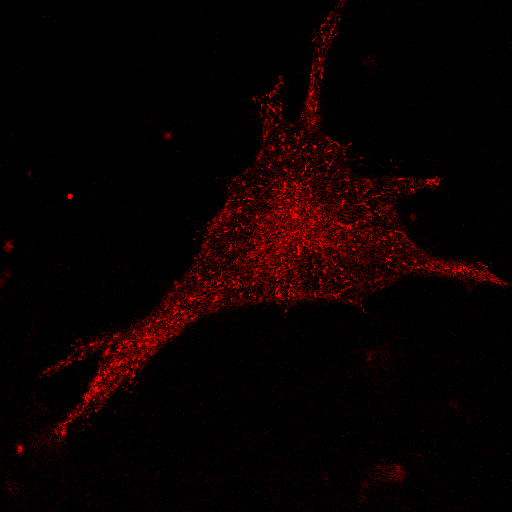

Supplement: Supplementary file 4 — Movie S2A [file 41380_2019_563_MOESM4_ESM.gif]

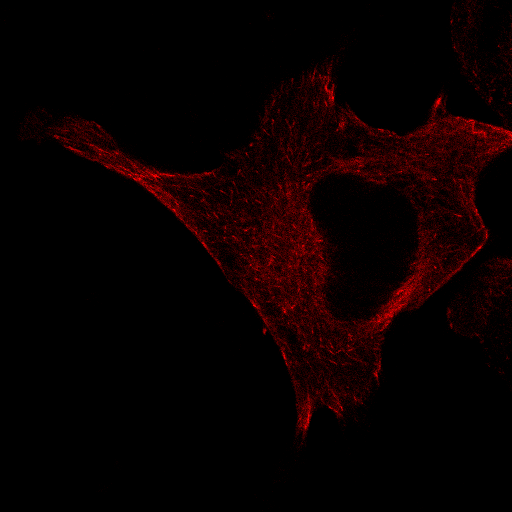

Supplement: Supplementary file 5 — Movie S2B [file 41380_2019_563_MOESM5_ESM.gif]

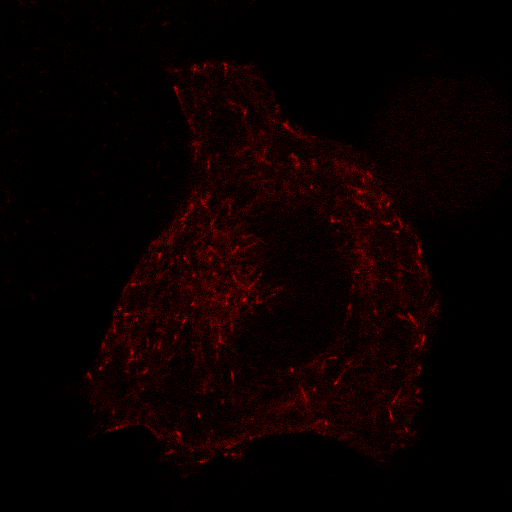

Supplement: Supplementary file 6 — Movie S3A [file 41380_2019_563_MOESM6_ESM.gif]

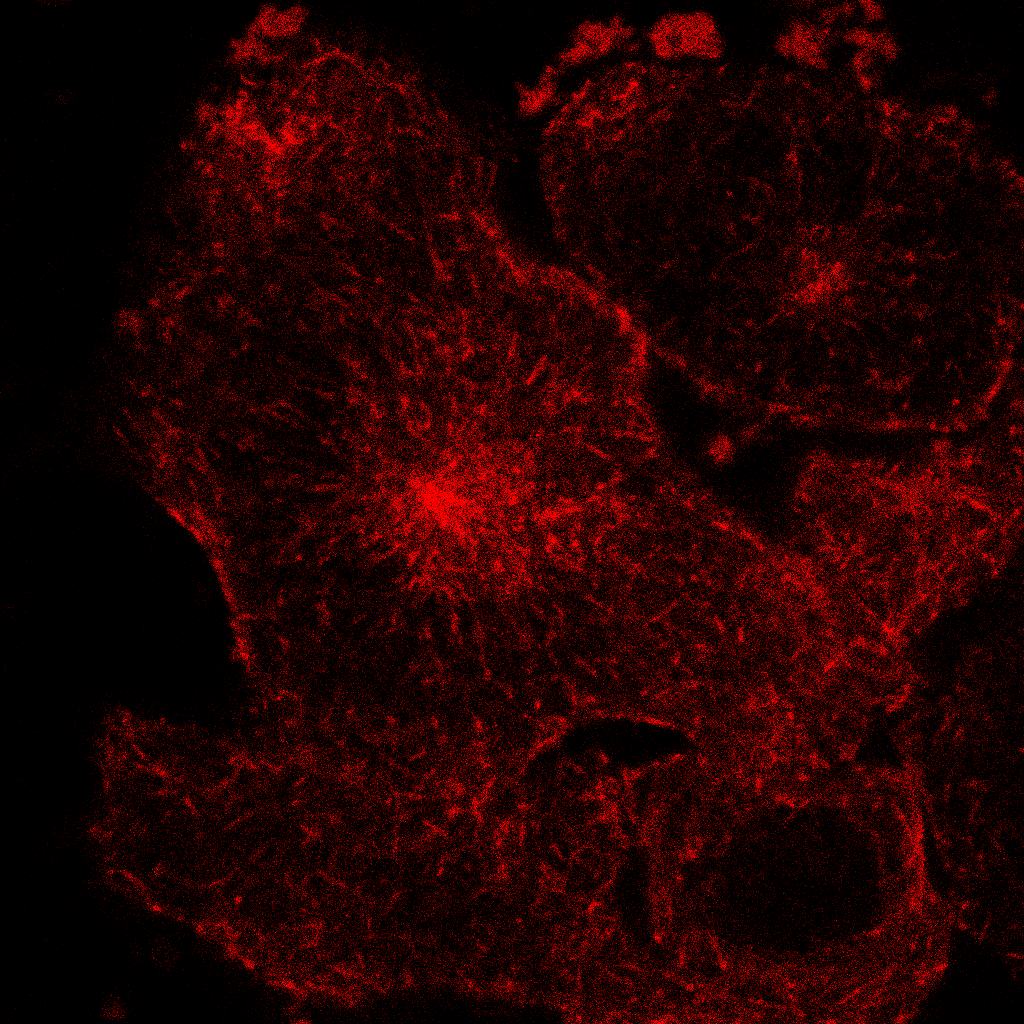

Supplement: Supplementary file 7 — Movie S3B [file 41380_2019_563_MOESM7_ESM.gif]

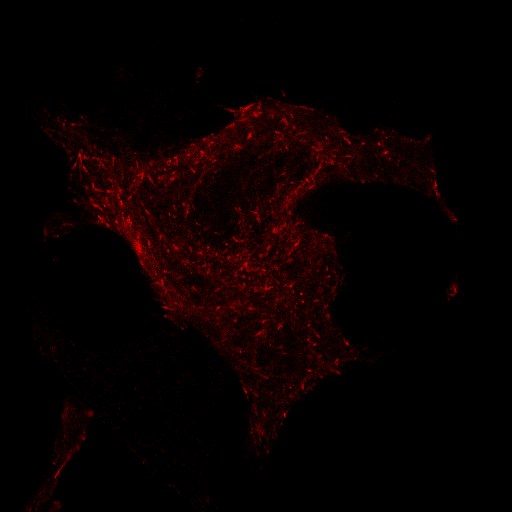

Supplement: Supplementary file 8 — Movie S4A [file 41380_2019_563_MOESM8_ESM.gif]

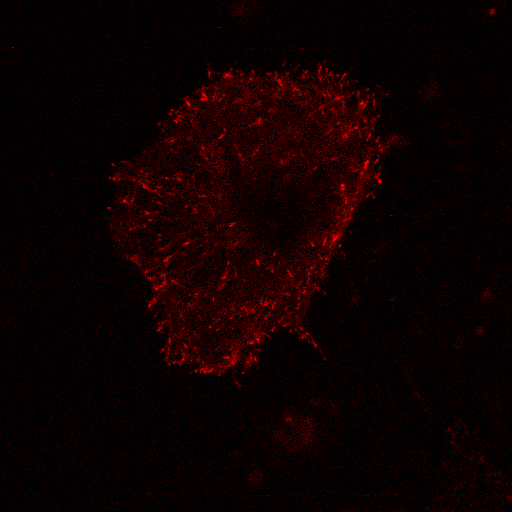

Supplement: Supplementary file 9 — Movie S4B [file 41380_2019_563_MOESM9_ESM.gif]
